# Targeting Cancer with New Morpholine-Benzimidazole-Oxadiazole Derivatives: Synthesis, Biological Evaluation, and Computational Insights

**DOI:** 10.1021/acsomega.5c03795

**Published:** 2025-07-30

**Authors:** Gresa Halimi Syla, Derya Osmaniye, Büşra Korkut Çelikateş, Yusuf Özkay, Zafer Asım Kaplancıklı

**Affiliations:** † Department of Pharmaceutical Chemistry, Faculty of Pharmacy, 52944 Anadolu University, Eskişehir 26470, Turkey; ‡ Institute of Graduate Education, Anadolu University, Eskişehir 26470, Turkey; § Central Analysis Laboratory, Faculty of Pharmacy, Anadolu University, Eskişehir 26470, Turkey; ∥ Department of Pharmaceutical Toxicology, Faculty of Pharmacy, Anadolu University, Eskişehir 26470, Turkey; ⊥ Department of Pharmacy Services, Vocational School of Health Services, Bilecik Seyh Edebali University, Bilecik 11230, Turkey

## Abstract

Cancer remains one of the leading causes of mortality worldwide, characterized by uncontrolled cell proliferation, invasion of surrounding tissues, and metastasis to distant organs. Among various malignancies, colon cancer is particularly aggressive and often associated with poor prognosis in advanced stages. This study presents the design, synthesis, and biological evaluation of a new series of morpholine-benzimidazole-oxadiazole derivatives as potential anticancer agents. The anticancer potential of the synthesized derivatives was assessed through MTT assays against the human colon cancer cell line (HT-29) and normal fibroblast cells (NIH3T3) to evaluate their selectivity. To further investigate their mechanism of action, VEGFR-2 enzyme inhibition assays were conducted, as VEGFR-2 plays a crucial role in angiogenesis and tumor progression. Compound **5h** exhibited potent VEGFR-2 inhibition (IC_50_ = 0.049 ± 0.002 μM), comparable to the reference drug sorafenib (IC_50_ = 0.037 ± 0.001 μM), while compounds **5j** (IC_50_ = 0.098 ± 0.011 μM) and **5c** (IC_50_ = 0.915 ± 0.027 μM) also showed notable inhibitory effects. Structural analysis suggested that the presence of chlorine atoms at both the third and fourth positions in the phenyl ring of compound **5h** enhanced its binding affinity within the ATP-binding pocket of VEGFR-2, contributing to its potent inhibition. Moreover, in silico studies (molecular docking and molecular dynamics simulations) confirmed that compounds **5c**, **5h**, and **5j** effectively interact with the VEGFR-2 active site and exhibit stability throughout the simulation period, reinforcing their potential as sustained VEGFR-2 inhibitors. These results highlight the promising therapeutic potential of morpholine-benzimidazole-oxadiazole derivatives as selective VEGFR-2 inhibitors for the treatment of colon cancer.

## Graphical abstract

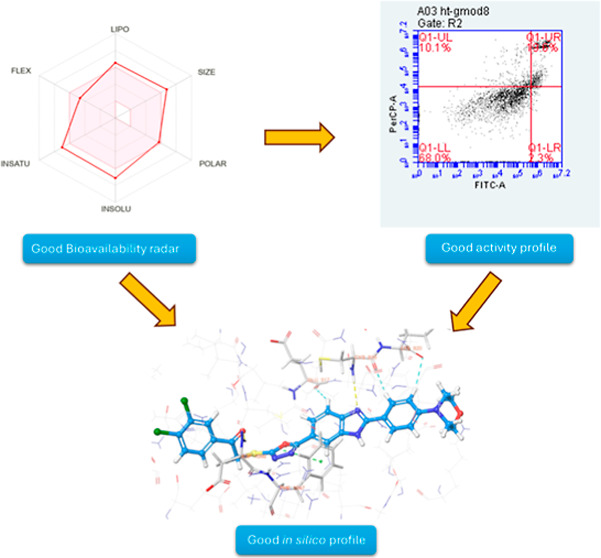

## 1 Introduction

Cancer remains a leading cause of mortality worldwide, involving unregulated cell proliferation, invasion of surrounding tissues, and metastasis to distant sites.^1,2^ Among various malignancies, colon cancer is one of the most prevalent and aggressive types, often associated with a poor prognosis in advanced stages due to late diagnosis and limited treatment options.^3^ Despite advancements in cancer therapy, the development of drug resistance and tumor recurrence necessitates the search for novel and more effective therapeutic agents.

One of the key hallmarks of cancer progression is angiogenesis, which involves the development of new blood vessels supplying tumors with oxygen and nutrients, thereby promoting growth and metastasis of the tumor. VEGFR-2 (Vascular endothelial growth factor receptor-2) is a critical regulator of angiogenesis and plays a crucial role in endothelial differentiation in colon cancer cells. Given its essential function in tumor progression, VEGFR-2 has been identified as a promising therapeutic target for antiangiogenic cancer therapy, and its inhibition has been explored in various anticancer drug development strategies.^4–9^

The development of VEGFR-2 inhibitors has been guided by key pharmacophoric features essential for effective binding and inhibition of the kinase domain. Typically, these inhibitors contain a hinge-binding motif, which establishes hydrogen bonding interactions involved in binding with the ATP-binding pocket of VEGFR-2, ensuring strong affinity and selectivity. Hydrophobic interactions, facilitated by lipophilic groups such as aromatic or heterocyclic rings, enhance binding to the hydrophobic pocket of VEGFR-2. An essential hydrogen bond donor/acceptor region further stabilizes the inhibitor within the active site, while a flexible linker or core scaffold allows for optimal interactions with the enzyme, improving inhibitory activity and selectivity. Additionally, electron-rich substituents such as morpholine, oxadiazole, and benzimidazole contribute to enhanced water solubility, metabolic stability, and bioavailability, making them crucial for the design of potent VEGFR-2 inhibitors.^10–14^

Benzimidazole is a bicyclical aromatic organic molecule composed by the fusion of a benzene ring with an imidazole ring at its 4 and 5 positions. The broad-spectrum pharmacological activities of benzimidazole and its derivatives have made them a major focus in the field of medicinal chemistry, including anticancer, antimicrobial, antiviral, antiulcer, antifungal, proton pump inhibitor, antihypertensive, anticonvulsant, antimalarial and anti-inflammatory properties.^15–33^ The most significant sites for substitution on the benzimidazole ring to develop biologically active derivatives are the 1, 2, 5, and 6. Benzimidazole-based compounds have demonstrated promising anticancer potential by targeting key molecular pathways, including EGFR, VEGFR, and PI_3_K, which are crucial in tumor progression. Owing to their therapeutic versatility, several benzimidazole derivatives have received clinical approval for various therapeutic applications. For instance, albendazole, thiabendazole, and mebendazole are commonly employed as anthelmintic agents, while as proton pump inhibitors drugs in use are pantoprazole, omeprazole, and lansoprazole. Additionally, astemizole is an antihistamine drug, enviradene exhibits antiviral properties, and candesartan cilexetil and telmisartan are employed as antihypertensive drugs. Furthermore, benzimidazole scaffolds serve as key frameworks for drug development in various therapeutic areas.^15,16,32–38^

Figures 1–3 presents a selection of marketed drugs containing a benzimidazole core, along with their therapeutic indications and generic names.

**Figure 1:**
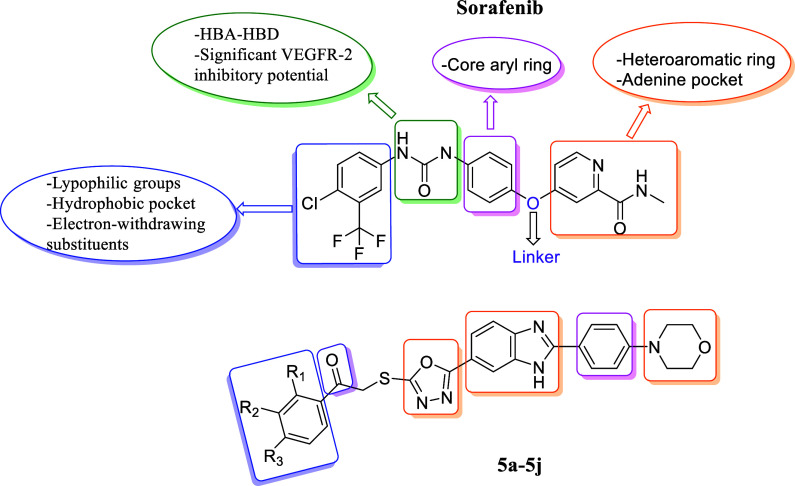
Rationale, design strategy, and structure–activity relationship (SAR) considerations for targeted VEGFR-2 inhibitors.

**2:**
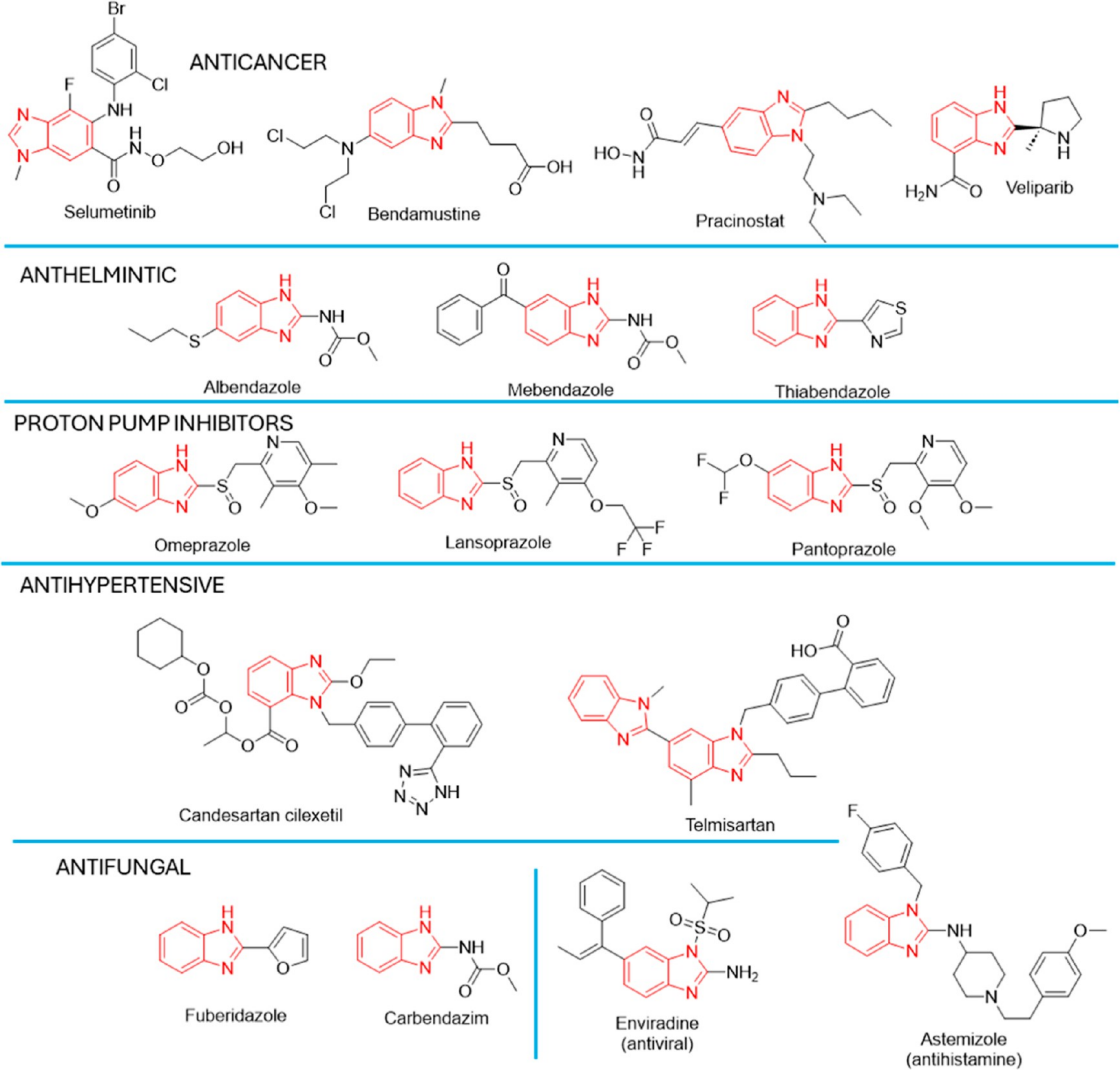
Clinically approved drugs featuring a benzimidazole scaffold.

**Figure 3:**
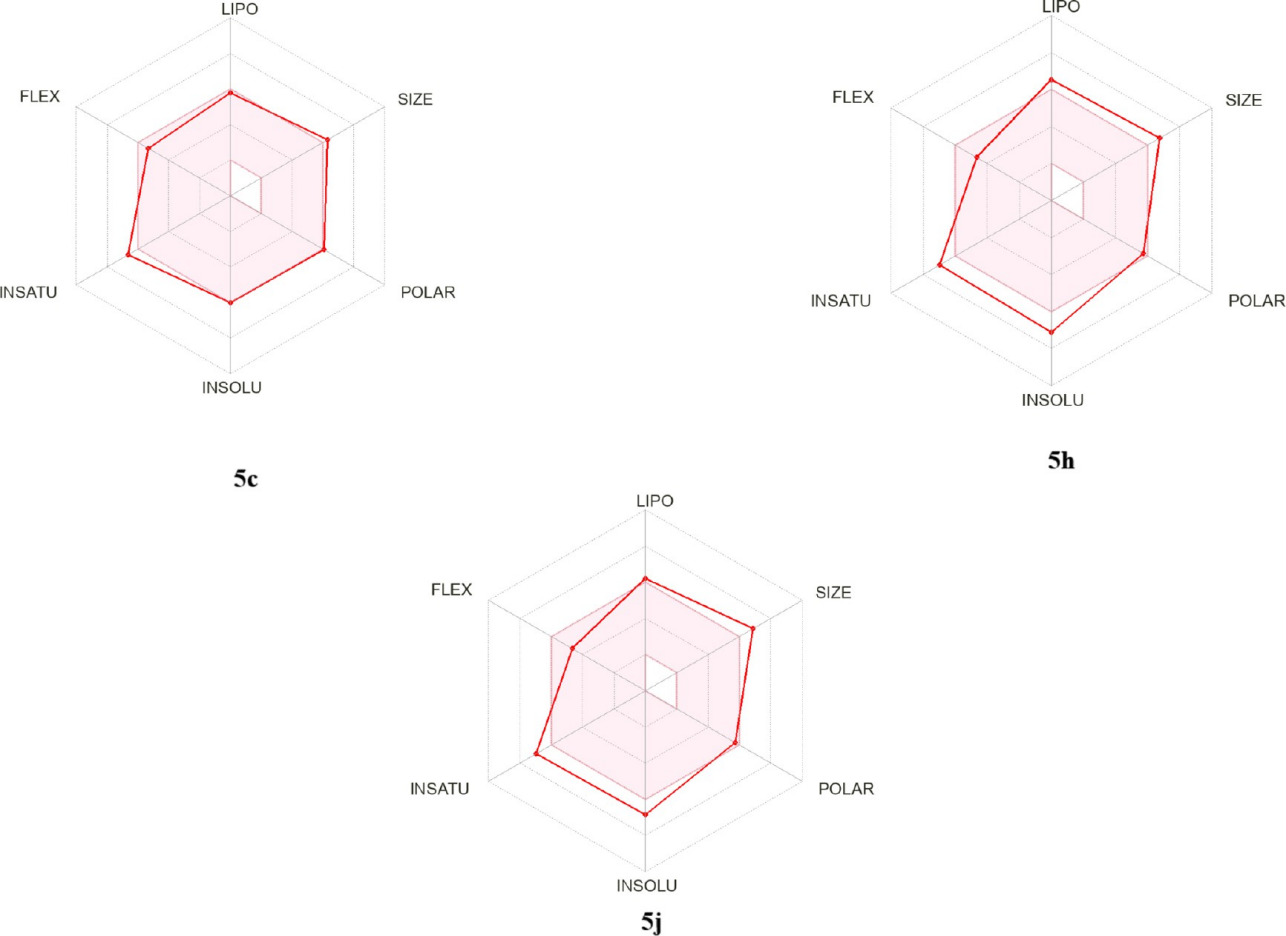
Bioavailability radars of the most active compounds **5c**, **5h** and **5j**.

Oxadiazole is a heterocyclic ring made up of five atoms: two carbons, two nitrogens, one oxygen atom, and characterized by two double bonds. Oxadiazoles represent a significant class of compounds which have garnered significant interest for their diverse biological and pharmacological activities. Among the different isomers, the 1,3,4-oxadiazole scaffold exhibits a broad spectrum of therapeutic effects. It has been recognized for its antibacterial, anticonvulsant, antitumor, antipyretic, antitubercular, antiviral, immunosuppressive, antioxidant, anti-inflammatory, and antihypertensive activities. These diverse pharmacological effects underscore the significant therapeutic potential of 1,3,4-oxadiazole derivatives in various medical applications, as extensively reported in the literature.^39–46^ Additionally, 1,3,4-oxadiazole heterocycles act as excellent bioisosteres for amides and esters, significantly enhancing pharmacological activity through their ability to form hydrogen bonds with target receptors.^47^ The morpholine ring has become increasingly popular in the development of bioactive compounds due to its unique structural properties. The presence of an electronegative oxygen atom enhances receptor binding through donor–acceptor interactions while simultaneously lowering the nitrogen’s basicity. Interestingly, ongoing research focuses on designing morpholine-containing anticancer agents with minimal or no adverse effects.^48,49^

Colon cancer, like other solid tumors, is dependent on new vessel formation (angiogenesis) for rapid growth and metastasis. One of the most important mediators of angiogenesis is the VEGF family, secreted by tumor cells and the tumor microenvironment. In colon cancer tissues, overexpression of VEGFR-2 triggers angiogenesis, supports the provision of nutrients and oxygen required for the tumor, and accelerates tumor growth. Therefore, targeting VEGFR-2 in colon cancer treatment is an important strategy for reducing tumor vascularization and inhibiting tumor growth.^50–56^ As mentioned in Figure 1, the compounds were designed based on sorafenib, a VEGFR-2 inhibitor. For this reason, colon cancer, where VEGFR-2 inhibitors are included in the clinical treatment procedure, was preferred in cancer cell line selection. In this study, we present the design and development, chemical synthesis, and biological assessment of a new set of morpholine-benzimidazole-oxadiazole derivatives as potential anticancer agents. The anticancer potential of the synthesized derivatives was assessed through MTT assays against the HT-29 cell line (human colon cancer cell line) and NIH3T3 (healthy normal fibroblast cells) to evaluate their selectivity. Among the tested compounds, **5h** exhibited the highest cytotoxic activity against HT-29 cells, with an IC_50_ of 3.103 ± 0.979 μM, while showing a significantly higher IC_50_ (15.158 ± 0.987 μM) against NIH3T3 cells, suggesting selectivity for cancer cells. Notably, compound **5h** demonstrated approximately 33-fold higher activity than compound **5e**, highlighting the crucial role of the additional chlorine atom at the phenyl ring’s 4-position in enhancing cytotoxicity. Additionally, compounds **5j** and **5c** demonstrated notable cytotoxicity against HT-29 cells, with IC_50_ values of 9.657 ± 0.149 μM and 17.750 ± 1.768 μM, respectively. Additionally, in silico studies (molecular docking and molecular dynamics simulation) were carried out to investigate the potential interactions of the compounds with VEGFR-2, aiming to elucidate the molecular mechanism underlying their anticancer activity.

By targeting VEGFR-2, these newly synthesized benzimidazole derivatives hold promise as potential antiangiogenic agents for colon cancer treatment. The findings from this study contribute to the ongoing search for selective and potent VEGFR-2 inhibitors with improved efficacy and therapeutic potential.

## 2 Material and Methods

### 2.1 Chemistry

#### 2.1.1 Synthesis of 4-Morpholinobenzaldehyde (1)

A solution of morpholine (10 mL, 0.1149 mol) was dissolved in 20 mL DMF, and 4-fluorobenzaldehyde (12.29 mL, 0.0990 mol) was added, then the reaction mixture was put in the microwave-assisted synthesis reactor (Anton-Paar Monowave 300). The reaction mixture was maintained for approximately 1 h in the microwave. The progress of the reaction was tracked by thin layer chromatography (TLC). After completion, the reaction mixture was poured into a beaker with 10 mL iced water to induce precipitation. The formed precipitate was collected by filtration and purified through crystallization with the use of ethanol and active charcoal.

#### 2.1.2 Synthesis of Methyl 2-(4-morpholinophenyl)-1*H*-benzo­[*d*]­imidazole-6-carboxylate (2)

A solution of methyl 3,4-diaminobenzoate (8.69 g, 0.0523 mol) and Na_2_S_2_O_5_ (9.94 g, 0.0523 mol) were dissolved in DMF (20 mL) and placed in the microwave-assisted synthesis reactor (Anton-Paar Monowave 300) for 10 min. It was then cooled and 4-morpholinobenzaldehyde (10 g, 0.0523 mol) was added. The reaction proceeded in the microwave for 1 h. After the reaction was completed, the reaction mixture was poured into a beaker with 10 mL ice-cold water to induce product precipitation. The precipitate was filtered and purified by crystallization using ethanol active charcoal.

#### 2.1.3 Synthesis of 2-(4-Morpholinophenyl)-1*H*-benzo­[*d*]­imidazole-6-carbohydrazide (3)

Methyl-2-(4-morpholinophenyl)-1*H*-benzo­[*d*]­imidazole-6-carboxylate (10 g, 0.0296 mol) was prepared by dissolving in ethanol (20 mL) followed by the addition of an excessive amount of hydrazine hydrate in ethanol in portions. The reaction was continued stirring for 3 h in reflux. After confirming the reaction complete, the solid product was collected by filtration, dried and purified through recrystallization from ethanol using actived charcoal.

[M + H]^+^ calcd for C_18_H_19_N_5_O_2_, 338.1612; found, 338.1598.

#### 2.1.4 Synthesis of 5-(2-(4-Morpholinophenyl)-1*H*-benzo­[*d*]­imidazol-6-yl)-1,3,4-oxadiazole-2-thiol (4)

2-(4-Morpholinophenyl)-1*H*-benzo­[*d*]­imidazole-6-carbohydrazide (5g, 0.014 mol) was introduced in an ethanolic NaOH solution (1.18 g, 0.0296 mol), then carbon disulfide was added (1.79 mL, 0.0235 mol). The mixture was subsequently refluxed for 8 h. After completion, the reaction mixture was poured into a beaker with 10 mL ice-cold water and then acidified with 21% HCl to pH 4–5. The precipitated solid was then filtered, rinsed with water, dried, and then purified through recrystallization using ethanol.

#### 2.1.5 Synthesis of 1-(Substitutedphenyl)-2-((5-(2-(4-morpholinophenyl)-1*H*-benzo­[*d*]­imidazol-6-yl)-1,3,4-oxadiazol-2-yl) Thio) Ethan-1-One (**5a**–**5j**)

A solution of 5-(2-(4-morpholinophenyl)-1*H*-benzo­[*d*]­imidazol-6-yl)-1,3,4-oxadiazole-2-thiol (0.3 g, 0.0007 mol) in acetone was mixed with the corresponding 4-substituted phenacyl bromide (0.0007 mol) and potassium carbonate (0.10 g, 0.0007 mol) and stirred at room temperature for 8 h. After completion, acetone was removed under reduced pressure, and the obtained product was rinsed with water, dried, and recrystallized using ethanol.

##### 2.1.5.1 4-Morpholinobenzaldehyde (**1**)

ESI-MS [M + H]^+^: 192.05.

##### 2.1.5.2 Methyl 2-(4-morpholinophenyl)-1*H*-benzo­[*d*]­imidazole-6-carboxylate (**2**)

ESI-MS [M + H]^+^: 338.10.

##### 2.1.5.3 2-(4-Morpholinophenyl)-1*H*-benzo­[*d*]­imidazole-6-carbohydrazide (**3**)

HRMS (*m*/*z*): [M + H]^+^ calcd for C_23_H_22_N_6_O_4_S_2_, 338.1612; found, 338.1598.

##### 2.1.5.4 5-(2-(4-Morpholinophenyl)-1*H*-benzo­[*d*]­imidazol-6-yl)-1,3,4-oxadiazole-2-thiol (**4**)

ESI-MS [M + H]^+^: 380.10.

##### 2.1.5.5 2-(5-(2-(4-Morpholinophenyl)-1*H*-benzo­[*d*]­imidazol-6-yl)-1,3,4-oxadiazol-2-yl thio)-1-phenylethan-1-One (**5a**)

Yield: 85%, mp = 227 °C, ^1^H NMR (300 MHz, DMSO-*d*_6_): δ = 3.26 (4H, s, morpholine), 3.76 (4H, s, morpholine), 5.20 (2H, s, -S-CH_2_), 7.11 (2H, d, *J* = 8.16 Hz, Ar–H), 7.61 (2H, t, *J* = 7.52 Hz, Ar–H), 7.73 (3H, t, *J* = 7.52 Hz, Ar–H), 8.05–8.10 (5H, m, Ar–H), 13.03 (1H, s-br., benzimidazole-NH). ^13^C NMR (75 MHz, DMSO-*d*_6_): δ = 40.98, 47.82, 66.42, 112.38, 114.82, 116.47, 119.75, 128.34, 128.98, 129.40, 130.35, 131.71, 134.49, 135.54, 138.19, 143.31, 147.44, 149.87, 152.81, 164.58, 193.28. ESI-MS [M + H]^+^: 498.10.

##### 2.1.5.6 2-(5-(2-(4-Morpholinophenyl)-1*H*-benzo­[*d*]­imidazol-6-yl)-1,3,4-oxadiazol-2-yl thio)-1-(*p*-tolyl) Ethan-1-One (**5b**)

Yield: 80%, mp = 243 °C, ^1^H NMR (300 MHz, DMSO-*d*_6_): δ = 2.41 (3H, s, –CH_3_), 3.26 (4H, s, morpholine), 3.76 (4H, s, morpholine), 5.16 (2H, s, -S-CH_2_), 7.11 (2H, d, *J* = 8.02 Hz, Ar–H), 7.40 (2H, d, *J* = 7.50 Hz, Ar–H), 7.62–7.78 (2H, m, Ar–H), 7.99 (3H, d, *J* = 6.50 Hz, Ar–H), 8.06 (2H, m, Ar–H), 13.02 (1H, s-br., benzimidazole-NH). ^13^C NMR (75 MHz, DMSO-*d*_6_): δ = 21.73, 40.89, 47.82, 66.41, 114.81, 120.50, 121.00, 124.04, 128.29, 129.09, 129.93, 133.04, 141.13, 143.49, 145.07, 147.11, 147.53, 152.00, 153.17, 154.96, 166.70, 192.77. ESI-MS [M + H]^+^: 512.10.

##### 2.1.5.7 1-(4-Methoxyphenyl)-2-(5-(2-(4-morpholinophenyl)-1*H*-benzo­[*d*]­imidazol-6-yl-1,3,4-oxadiazol-2-yl) Thio) Ethan-1-One (**5c**)

Yield: 76%, mp = 240 °C, ^1^H NMR (300 MHz, DMSO-*d*_6_): δ = 3.26 (4H, s, morpholine), 3.76 (4H, s, morpholine), 3.87 (3H, s, –O–CH_3_), 5.13 (2H, s, -S-CH_2_), 7.11 (4H, d, *J* = 8.21 Hz, Ar–H), 7.75–7.77 (2H, m, Ar–H), 8.06 (5H, d, *J* = 8.10 Hz, Ar–H), 13.02 (1H, s-br., benzimidazole-NH). ^13^C NMR (75 MHz, DMSO-*d*_6_): δ = 40.74, 47.82, 56.14, 66.41, 114.62, 114.81, 116.67, 119.77, 120.68, 120.73, 128.34, 128.38, 131.41, 144.70, 146.76, 148.58, 152.82, 155.89, 163.03, 164.24, 166.54, 191.55. ESI-MS [M + H]^+^: 528.10.

##### 2.1.5.8 1-(4-Fluorophenyl)-2-((5-(2-(4-morpholinophenyl)-1*H*-benzo­[*d*]­imidazol-6-yl)-1,3,4-oxadiazol-2-yl) Thio) Ethan-1-One (**5d**)

Yield: 82%, mp = 239 °C, ^1^H NMR (300 MHz, DMSO-*d*_6_): δ = 3.26 (4H, s, morpholine), 3.76 (4H, s, morpholine), 5.19 (2H, s, -S-CH_2_), 7.11 (2H, d, *J* = 8.24 Hz, Ar–H), 7.44 (2H, t, *J* = 8.49 Hz, Ar–H), 7.69–7.77 (2H, m, Ar–H), 8.06 (3H, d, *J* = 8.21 Hz, Ar–H), 8.18 (2H, t, *J* = 6.16 Hz, Ar–H), 13.03 (1H, s-br., benzimidazole-NH). ^13^C NMR (75 MHz, DMSO-*d*_6_): δ = 40.84, 47.82, 66.41, 114.81, 116.38, 116.59, 119.74, 120.72, 128.34, 128.91 (d, *J*_*CF*‑3_ = 9.7 Hz), 132.19 (d, *J*_*CF*‑2_ = 21.7 Hz), 132.31, 132.34, 152.83, 162.87, 163.15 (1C, d, *J*_*CF*‑1_ = 245 Hz), 166.60, 167.19, 191.96. ESI-MS [M + H]^+^: 516.10.

##### 2.1.5.9 1-(4-Chlorophenyl)-2-((5-(2-(4-morpholinophenyl)-1*H*-benzo­[*d*]­imidazol-6-yl)-1,3,4-oxadiazol-2-yl) Thio) Ethan-1-One (**5e**)

Yield: 72%, mp = 242 °C, ^1^H NMR (300 MHz, DMSO-*d*_6_): δ = 3.26 (4H, s, morpholine), 3.76 (4H, s, morpholine), 5.24 (2H, s, -S-CH_2_), 7.11 (2H, d, *J* = 7.63 Hz, Ar–H), 7.75 (2H, s-br., Ar–H), 8.05 (3H, d, *J* = 7.88 Hz, Ar–H), 8.31 (2H, d, *J* = 7.70 Hz, Ar–H), 8.40 (2H, d, *J* = 7.80 Hz, Ar–H), 13.02 (1H, s-br., benzimidazole-NH). ^13^C NMR (75 MHz, DMSO-*d*_6_): δ = 40.83, 47.81, 66.41, 114.80, 116.57, 119.75, 120.68, 121.06, 122.10, 124.45, 128.34, 130.41, 132.37, 132.82, 136.25, 140.19, 150.77, 152.82, 166.62, 166.68, 192.77. ESI-MS [M + H]^+^: 532.05.

##### 2.1.5.10 4-(2-(5-(2-(4-Morpholinophenyl)-1*H*-benzo­[*d*]­imidazol-6-yl-1,3,4-oxadiazol-2-yl) Thio) Acetyl) Benzonitrile (**5f**)

Yield: 65%, mp = 230 °C, ^1^H NMR (300 MHz, DMSO-*d*_6_): δ = 3.26 (4H, s, morpholine), 3.76 (4H, s, morpholine), 5.22 (2H, s, -S-CH_2_), 7.11 (2H, d, *J* = 8.31 Hz, Ar–H), 7.75–7.77 (2H, m, Ar–H), 8.05–8.08 (5H, m, Ar–H), 8.23 (2H, d, *J* = 7.80 Hz, Ar–H), 13.03 (1H, s-br., benzimidazole-NH). ^13^C NMR (75 MHz, DMSO-*d*_6_): δ = 40.94, 47.81, 66.41, 114.81, 116.27, 116.59, 117.88, 118.54, 119.74, 120.70, 122.42, 128.34, 129.58, 133.41, 137.33, 138.76, 141.38, 152.83, 162.66, 166.67, 192.94. ESI-MS [M + H]^+^: 523.10.

##### 2.1.5.11 2-(5-(2-(4-Morpholinophenyl)-1*H*-benzo­[*d*]­imidazol-6-yl-1,3,4-oxadiazol-2-yl) thio)-1-(4-nitrophenyl) Ethan-1-One (**5g**)

Yield: 70%, mp = 233 °C, ^1^H NMR (300 MHz, DMSO-*d*_6_): δ = 3.26 (4H, s, morpholine), 3.76 (4H, s, morpholine), 5.24 (2H, s, -S-CH_2_), 7.11 (2H, d, *J* = 8.09 Hz, Ar–H), 7.75 (2H, s-br., Ar–H), 8.05 (3H, d, *J* = 7.75 Hz, Ar–H), 8.31 (2H, d, *J* = 7.76 Hz, Ar–H), 8.40 (2H, d, *J* = 7.86 Hz, Ar–H), 13.02 (1H, s-br., benzimidazole-NH). ^13^C NMR (75 MHz, DMSO-*d*_6_): δ = 41.08, 47.81, 66.41, 114.80, 116.57, 119.75, 120.68, 121.06, 122.10, 124.45, 125.63, 126.30, 128.34, 130.41, 132.37, 140.19, 150.77, 152.82, 162.64, 166.68, 192.77. ESI-MS [M + H]^+^: 543.05.

##### 2.1.5.12 1-(3,4-Dichlorophenyl)-2-((5-(2-(4-morpholinophenyl)-1*H*-benzo­[*d*]­imidazol-6-yl)-1,3,4-oxadiazol-2-yl) Thio) Ethan-1-One (**5h**)

Yield: 76%, mp = 240 °C, ^1^H NMR (300 MHz, DMSO-*d*_6_): δ = 3.26 (4H, s, morpholine), 3.76 (4H, s, morpholine), 5.18 (2H, s, -S-CH_2_), 7.11 (2H, d, *J* = 8.17 Hz, Ar–H), 7.68–7.77 (2H, m, Ar–H), 7.88 (1H, d, *J* = 8.42 Hz, Ar–H), 8.02–8.07 (4H, m, Ar–H), 8.31 (1H, s, Ar–H), 13.03 (1H, s-br., benzimidazole-NH). ^13^C NMR (75 MHz, DMSO-*d*_6_): δ = 40.73, 47.81, 66.41, 114.80, 116.59, 119.76, 120.67, 126.70, 127.48, 128.34, 128.93, 130.92, 131.74, 132.45, 135.70, 137.29, 152.82, 162.64, 166.69, 191.77. ESI-MS [M + H]^+^: 566.00.

##### 2.1.5.13 1-(2,4-Dimethylphenyl)-2-((5-(2-(4-morpholinophenyl)-1*H*-benzo­[*d*]­imidazol-6-yl)-1,3,4-oxadiazol-2-yl) Thio) Ethan-1-One (**5i**)

Yield: 81%, mp = 166 °C, ^1^H NMR (300 MHz, DMSO-*d*_6_): δ = 2.33–2.36 (3H, m, –CH_3_), 2.40 (3H, s, –CH_3_), 3.27 (4H, s, morpholine), 3.76 (4H, s, morpholine), 5.06 (2H, s, -S-CH_2_), 7.11–7.22 (4H, m, Ar–H), 7.69 (1H, d, *J* = 8.01 Hz, Ar–H), 7.77–7.85 (1H, m, Ar–H), 7.92 (1H, d, *J* = 7.50 Hz, Ar–H), 8.07 (3H, s-br., Ar–H). ^13^C NMR (75 MHz, DMSO-*d*_6_): δ = 21.38, 21.44, 42.82, 47.79, 66.41, 114.79, 126.90, 127.02, 128.42, 130.27, 130.40, 132.98, 133.06, 133.12, 138.72, 138.80, 142.84, 143.06, 152.90, 162.86, 166.49, 195.67. ESI-MS [M + H]^+^: 526.10.

##### 2.1.5.14 1-(4-Bromophenyl)-2-((5-(2-(4-morpholinophenyl)-1*H*-benzo­[*d*]­imidazol-6-yl)-1,3,4-oxadiazol-2-yl) Thio) Ethan-1-One (**5j**)

Yield: 84%, mp = 232 °C, ^1^H NMR (300 MHz, DMSO-*d*_6_): δ = 3.26 (4H, s, morpholine), 3.76 (4H, s, morpholine), 5.17 (2H, s, -S-CH_2_), 7.11 (2H, d, *J* = 8.31 Hz, Ar–H), 7.74 (2H, s-br., Ar–H), 7.82 (2H, d, *J* = 7.86 Hz, Ar–H), 8.05–8.07 (5H, m, Ar–H), 13.03 (1H, s-br., benzimidazole-NH). ^13^C NMR (75 MHz, DMSO-*d*_6_): δ = 40.83, 47.82, 66.41, 114.81, 116.61, 119.77, 122.66, 123.45, 128.34, 128.66, 130.31, 130.96, 132.48, 134.57, 145.13, 145.72, 146.87, 152.85, 162.63, 166.62, 192.67. ESI-MS [M + H]^+^: 578.00.

### 2.2 Computational In Silico ADME Studies

The synthesized compounds **5a**–**5j** were assessed for their molecular properties using the SwissADME web platform. This analysis encompassed a range of factors, such as pharmacokinetic behavior, physicochemical characteristics, the drug likeness, lipophilicity, and medicinal chemistry aspects, with results presented in Table 1.^57^

**Table 1:** Target Compound’s Physicochemical Characteristics, Pharmacokinetic Profile, Drug Likeness Attributes and Synthetic Accessibility (**5a**–**5j**)­^a^

	physicochemical properties							pharmacokinetics		druglikness	Med.Chem
	*M*_W_	**RB**	**HBA**	**HBD**	**TPSA**	C Log *P*	Log *S*	**GIA**	**BBBp**	L-RoF (**V**)	**SA**
**5a**	497.57	7	6	1	122.44	4.02	–5.92	low	no	yes (0)	3.83
**5b**	511.59	7	6	1	122.44	4.29	–6.23	low	no	yes (1) *M*_W_ > 500	3.94
**5c**	527.59	8	7	1	131.67	4.05	–6.00	low	no	yes (1) *M*_W_ > 500	3.94
**5d**	515.56	7	7	1	122.44	4.37	–6.08	low	no	yes (1) *M*_W_ > 500	3.83
**5e**	532.01	7	6	1	122.44	4.59	–6.52	low	no	yes (1) *M*_W_ > 500	3.82
**5f**	522.58	7	7	1	146.23	3.83	–5.87	low	no	yes (1) *M*_W_ > 500	3.90
**5g**	542.57	8	8	1	168.26	3.35	–5.99	low	no	yes (2) *M*_W_ > 500 NorO >10	3.96
**5h**	566.46	7	6	1	122.44	5.10	–7.11	low	no	yes (1) *M*_W_ > 500	3.84
**5i**	525.62	7	6	1	122.44	4.72	–6.53	low	no	yes (1) *M*_W_ > 500	4.07
**5j**	576.46	7	6	1	122.44	4.68	–6.84	low	no	yes (1) *M*_W_ > 500	3.85

a RB; rotatable bonds, HBA ; hydrogen bond acceptors, HBD; hydrogen bond donors, TPSA; topological polar surface area, C Log *P*; calculated log *P*, Log *S*; logarithm of solubility, GIA; gastrointestinal absorption, BBBp; blood–brain barrier permeability, L-Rof (V); Lipinski rule of five, SA; synthetic accessibility.

### 2.3 Anticancer Activity Evaluation

#### 2.3.1 Cytotoxicity Studies

Compounds **5a**–**5j** were evaluated for their anticancer activity via the MTT assay against the HT-29 cell line. MIC values were determined using fluorometric methods, with sorafenib as reference drug. Both the synthesized compounds and standards were tested across concentrations from 1000 to 0.49 μg/mL. The results are presented in Table 2.^58–60^

**Table 2:** IC_50_ (μM) Values of Synthesized Compounds (**5a**–**5j**)­^a^

compounds	HT29	NIH3T3	compounds	HT29	NIH3T3
**5a**	135.465 ± 3.372	192.545 ± 7.555	**5f**	30.200 ± 1.161	14.682 ± 0.818
**5b**	22.721 ± 2.388	10.465 ± 2.172	**5g**	259.624 ± 8.684	>1000
**5c**	17.750 ± 1.768	10.416 ± 0.205	**5h**	3.103 ± 0.979	15.158 ± 0.987
**5d**	174.815 ± 5.915	163.147 ± 3.755	**5i**	72.148 ± 4.611	148.244 ± 3.987
**5e**	103.668 ± 4.526	530.678 ± 8.098	**5j**	9.657 ± 0.149	4.546 ± 0.763
**Sorafenib**	2.919 ± 0.214	30.134 ± 0.147			

a The test results were reported as the mean values of four independent assays.

#### 2.3.2 Flow Cytometry Analysis

Programmed cell death-apoptosis is a normal physiological process in healthy organisms that eliminates cells no longer needed. During apoptosis, phosphatidylserine (PS) in the cell membrane is translocated to the outer layer. The Annexin V protein included in the kit binds to this externalized PS, enabling its detection with FITC, a fluorescent marker. Annexin V can also bind to necrotic cells. In such cases, the cells are identified using “Ethidium Homodimer III (EthD-III) (or propidium iodide, PI)”. Flow cytometric assay kits use double-staining protocols to assess apoptosis by detecting differences in cell morphology through the binding efficiency of the Annexin–V complex to PS on the cell surface.^61,62^ Apoptotic, Necrotic, and Healthy Cells Detection Kit [Available from (Apoptic necrotic kit)] used during flow cytometer analysis.^63^

Briefly, HT-29 cells were plated in six-well plates following the manufacturer’s guidelines and exposed to the compounds at their IC_50_ concentrations for 24 h. Following treatment, cell suspensions were centrifuged at 1200 rpm for 5 min, and the resulting pellets were rinsed twice with 1 mL of cold phosphate-buffered saline. The assay kits were used according to the manufacturer’s instructions by transferring cells to the appropriate buffer solutions. For each sample, at least 10 000 cells were evaluated. Cell population fractions in various quadrants and gates were determined using quadrant and gate statistical analysis, with settings optimized based on comparison to the untreated control group.

### 2.4 Molecular Docking

Molecular docking studies were performed to investigate the binding interactions between the synthesized compounds and VEGFR-2, aiming to elucidate their potential mechanism of anticancer activity. Compounds **5c**, **5h** and **5j** found to be the most promising in activity studies and were further examined for their binding affinity within enzyme active sites using in silico docking studies. These studies employed the active site of the VEGFR-2 enzyme, retrieved from the Protein Data Bank (PDB ID: 4ASD).^64^ The docking simulations were carried out using Glide 7.1 software.^65–67^

### 2.5 Molecular Dynamics Simulations

Molecular dynamics simulations (MDS) use groups of the Schrödinger Suite to determine the stability of selected samples with the target enzyme and the connectivity of the linkages. The detailed protocol and steps taken for simulations using Desmond (Schrödinger’s MD module) and Maestro (Schrödinger’s graphical user interface) have been previously reported by our system^66,68–76^

## 3 Results and Discussion

### 3.1 Chemistry

Compounds **5a**–**5j** were synthesized following the synthetic pathway illustrated in Scheme 1. **Compound 1** was prepared by reacting morpholine with 4-Fluorobenzaldehyde in DMF under microwave irradiation. Subsequently, Methyl 2-(4-morpholinophenyl)-1*H*-benzo­[*d*]­imidazole-6-carboxylate (**compound 2**) was obtained by reacting methyl 3,4-diaminobenzoate with 4-morpholinobenzaldehyde in the presence of Na_2_S_2_O_5_ in DMF, using microwave irradiation for 1 h. The next step involved the synthesis of **compound 3** (2-(4-Morpholinophenyl)-1*H*-benzo­[*d*]­imidazole-6-carbohydrazide), achieved by the gradual addition of hydrazine hydrate in ethanol to **compound 2**. The oxadiazole ring was subsequently formed by refluxing **compound 3** with CS_2_ under basic conditions in ethanol for 8 h, yielding the intermediate 5-(2-(4-morpholinophenyl)-1*H*-benzo­[*d*]­imidazol-6-yl)-1,3,4-oxadiazole-2-thiol. Finally, the target compounds (**5a**–**5j**) were synthesized through the reaction of 5-(2-(4-Morpholinophenyl)-1*H*-benzo­[*d*]­imidazol-6-yl)-1,3,4-oxadiazole-2-thiol with the corresponding 4-Substituted phenacyl bromides in acetone, using potassium carbonate as a catalysator.

**Scheme 1:**
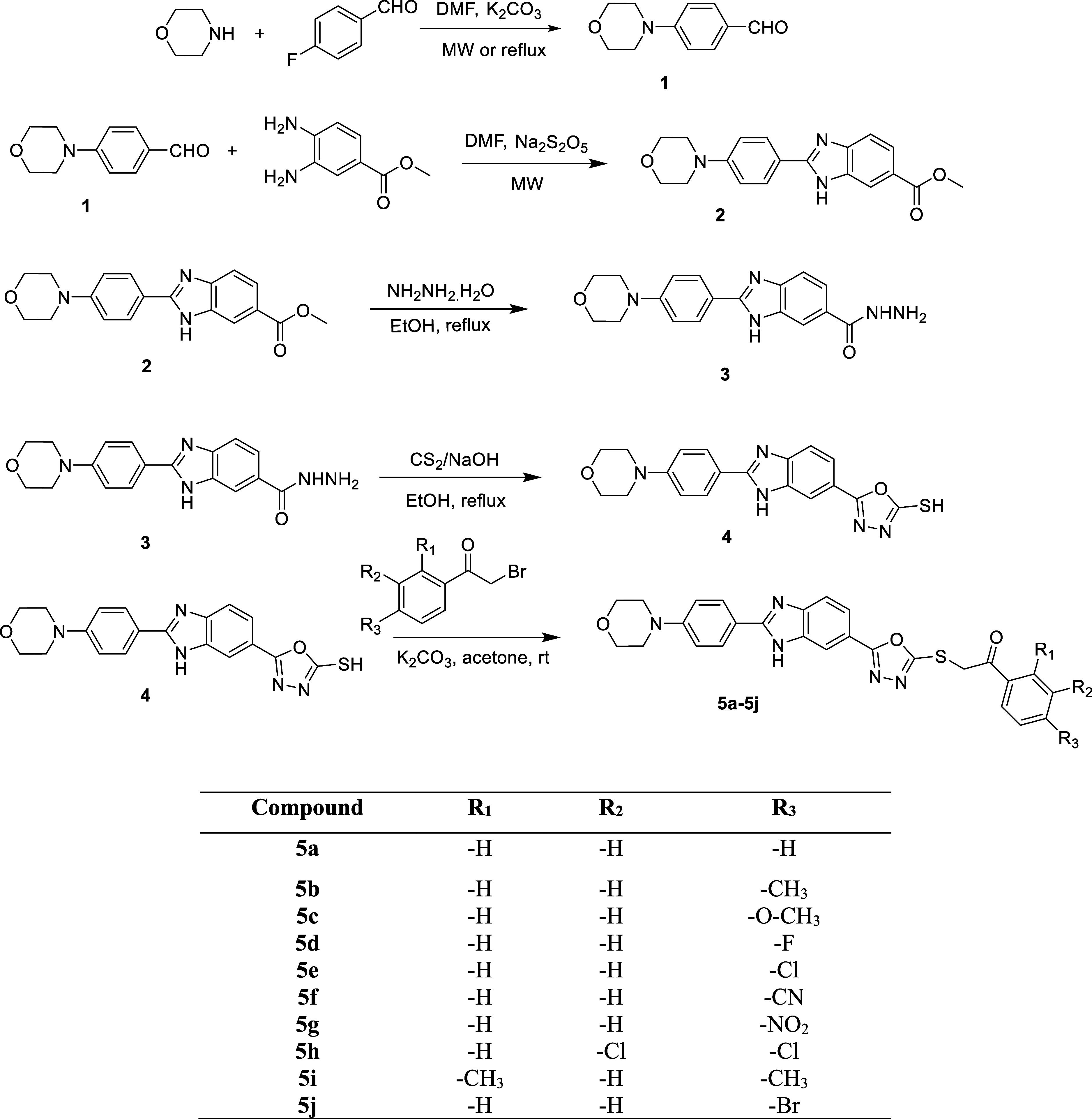
Method Employed for the Synthesis of Compounds **5a**–**5j**

The structures of the synthesized compounds (**5a**–**5j**) were confirmed using NMR analyses (^1^H NMR, ^13^C NMR). The results confirmed the presence of key functional groups, including morpholine, benzimidazole, oxadiazole, and phenyl rings, as well as carbonyl groups and substituted benzene moieties within the molecular framework.

^1^H NMR spectral analysis revealed consistent proton signal patterns across all compounds (**5a**–**5j**). The morpholine ring protons exhibited two distinct singlets in the range of 3.26–3.27 and 3.76 ppm. The aliphatic methylene protons, positioned adjacent to sulfur and the carbonyl group, appeared as a singlet between 5.06 and 5.24 ppm. Aromatic protons appeared in 7.10–8.41 ppm, whereas the NH proton of the benzimidazole ring was characterized by a broad singlet at 13.01–13.03 ppm.

Similarly, ^13^C NMR analysis demonstrated characteristic carbon shifts across all derivatives. The methylene carbons bonded to the carbonyl and sulfur groups resonated between 40.61 and 42.82 ppm. The morpholine ring carbons consistently appeared at 47.79–47.82 ppm and 66.41–66.42 ppm. The aromatic carbon signals appeared between 112.38 and 114.81 ppm, while the carbon of the carbonyl functional group resonance in all compounds was detected between 191.55 and 195.67 ppm.

These spectroscopic findings confirm the successful synthesis of compounds **5a**–**5j**, with structural assignments further supported by NMR data. Detailed spectroscopic data for each compound are provided in Supporting Information, Figures S1–S20.

### 3.2 In Silico ADME Studies

During the drug design process, various molecular properties are considered to assess the pharmacokinetic suitability of candidate molecules. In this context, current ADMET criteria, particularly Lipinski’s Rule of Five, are guiding in predicting the drug-like properties of compounds. The number of rotatable bonds (RB), which is related to the flexibility of the molecule, is generally preferred to be less than 10, as a high RB value can negatively impact bioavailability and membrane permeability. Similarly, it is recommended that the number of hydrogen bond acceptors (HBA) and donors (HBD) not exceed 10 and 5, respectively. Exceeding these limits may reduce the molecule’s permeation through the cell membrane and oral bioavailability.

Topological polar surface area (TPSA) is an important parameter reflecting the total polarity of the molecule. Compounds with a TPSA value below 140 Å^2^ are known to exhibit good oral bioavailability, while compounds below 90 Å^2^ have the potential to cross the blood–brain barrier. The CLogP value, which measures the molecule’s lipophilicity, should generally be between 0 and 5. This range represents an optimal balance between solubility and membrane permeability. Furthermore, the Log *S* value indicates the compound’s water solubility; values of −4 and above are associated with good solubility, while values below −6 carry the risk of low solubility.

The gastrointestinal absorption (GIA) parameter is important for drugs administered orally, and high GI absorption is preferred. The blood–brain barrier permeability (BBBp) parameter should be positive for drugs whose therapeutic target is the central nervous system, but a negative value may be advantageous for peripherally acting drug candidates. According to Lipinski’s Rule of Five, the molecular weight should be less than 500 Da, the CLog *P* should be below 5, the HBA number should be less than 10, and the HBD number should be less than 5. Violation of at least one of these four rules generally does not preclude a molecule from being a drug candidate. Finally, the synthetic accessibility (SA) value expresses the synthesizability of the compound and is rated from 1 to 10. Values below 6 are considered suitable for practical synthesis.

The physicochemical and pharmacokinetic properties, including ADME parameters, of compounds **5a**–**5j** were evaluated using SwissADME, a freely available online tool. The results are summarized in Table 1. The solubility assessment indicated that none of the compounds are expected to exhibit significant solubility issues, as their Log S values remain below 0 and do not exceed the threshold of 6. Most of the synthesized compounds exhibit high molecular flexibility, characterized by eight or fewer rotatable bonds. Furthermore, the analysis suggests that none of the compounds are likely to penetrate the blood–brain barrier (BBB), thereby minimizing the risk for side effects in the central nervous system (CNS). However, gastrointestinal (GI) absorption is predicted to be low across the series. Regarding drug-likeness, all compounds generally adhere to Lipinski’s Rule of Five, except for their molecular weights, which exceed 500 Da in most cases.

### 3.3 Anticancer Activity Studies

#### 3.3.1 Cytotoxicity Studies

The cytotoxic effects of the compounds on colon cancer (HT29, ATCC HTB-38) and normal healthy fibroblast (NIH3T3, ATCC CRL-1658) cell lines were evaluated using the MTT assay. The results obtained are shown in Table 2. Based on the cytotoxicity data **5c**, **5h** and **5j** stand out in terms of activity. However, compounds **5c** and **5j** were found to be cytotoxic. The selectivity indices (SI) of the compounds are shown in Figure 4.

**Figure 4:**
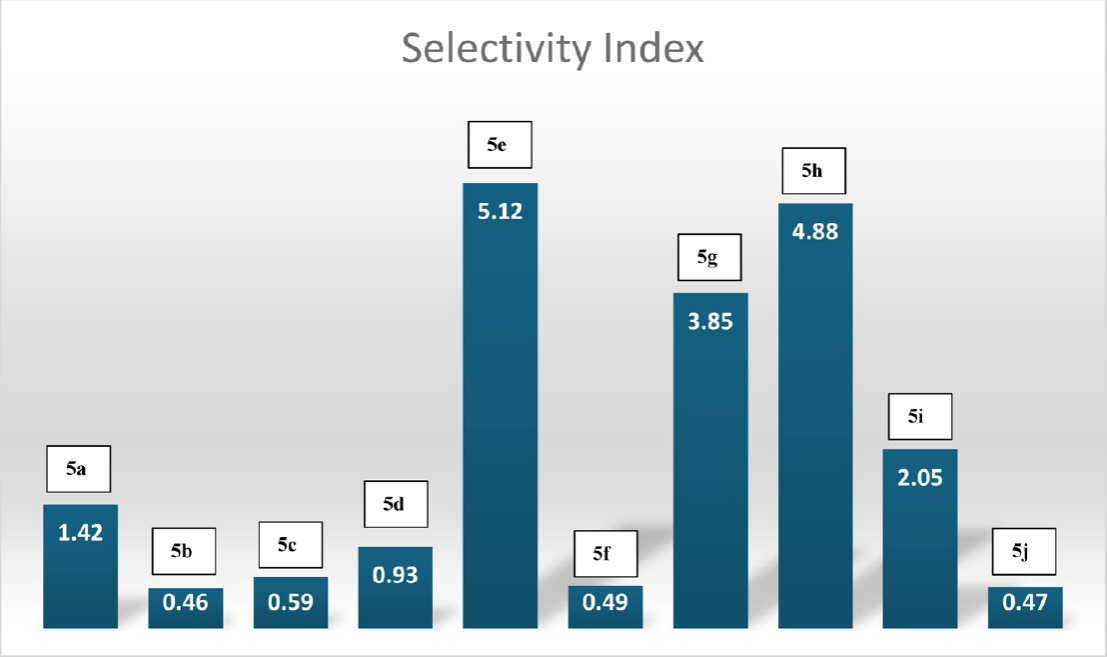
Selectivity index (SI) of compounds **5a**–**5j**.

Within the series, **5h** showed the greatest activity (IC_50_ = 3.103 ± 0.979 μM). Moreover, the selectivity index of 4.88 supports the hypothesis that the compound may be a safe therapeutic candidate as it does not show toxicity on healthy cells at the dose that is cytotoxic to cancer cells.

#### 3.3.2 In Vitro Analysis of VEGFR-2 Inhibition

The inhibitory effects of the synthesized compounds on the VEGFR-2 enzyme were assessed using an in vitro assay kit (Table 3). Sorafenib was employed as the reference standard in these evaluations. Compound **5h** exhibited a strong inhibitory effect, with an IC_50_ value of 0.049 ± 0.002 μM, closely matching the activity of sorafenib (IC_50_ = 0.037 ± 0.001 μM). This demonstrates that compound **5h** has significant VEGFR-2 inhibitory activity and could be a promising lead compound for further research.

**Table 3:** IC_50_ Values (μM) of Compounds **5c**, **5h**, **5j** and Sorafenib for VEGFR-2 Inhibition

compounds	IC_50_ values (μM)
**5c**	0.918 ± 0.027
**5h**	0.049 ± 0.002
**5j**	0.098 ± 0.011
**Sorafenib**	0.037 ± 0.001

These findings suggest that the substitution pattern found in compound **5h** may play a critical role in its potent VEGFR-2 inhibitory activity. The close similarity of the IC_50_ value to that of sorafenib, a clinically approved VEGFR-2 inhibitor, highlights its therapeutic potential. Notably, compound **5h** contains chlorine atoms at the 3 and 4 positions of the phenyl ring, a structural feature that may contribute to improved binding affinity within the ATP-binding site of the VEGFR-2 kinase domain.

#### 3.3.3 Flow Cytometry Analysis

Demonstration of anticancer activity via induction of apoptosis is considered a desirable and reliable mechanism of action, as apoptosis represents a genetically regulated and noninflammatory form of cell death. In contrast, it is an uncontrolled and unprogrammed process that can cause serious damage to surrounding tissues due to necrosis, inflammation, and cellular leakage. Therefore, apoptotic cell death is generally preferred for anticancer strategies in terms of safety and selectivity.

Based on the cytotoxicity and VEGFR-2 inhibition results, compound **5h** was determined as the most potent and least cytotoxic member of the series. To further investigate the mechanism underlying its anticancer effect, the ability of compound **5h** to induce apoptosis was assessed using the Annexin V-FITC/PI dual staining method. The results are presented in Figure 5, where the data are shown in four quadrants corresponding to different cell populations: live (living) cells correspond to Q1-LL, early apoptotic cells to Q1-LR, late apoptotic cells to Q1-UR, and necrotic cells to Q1-UL.

**Figure 5:**
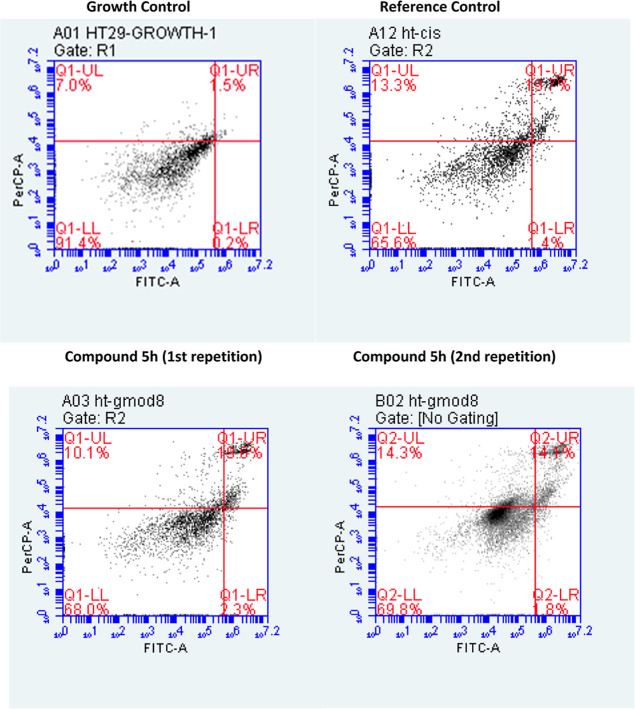
Flow cytometry analysis diagram of compound **5h** on the HT29 cell line.

A growth control group was included, in which only culture medium was applied on the second day instead of any inhibitor, to account for necrosis possibly induced by the cell treatment process, particularly through agents such as trypsin–EDTA. This group showed approximately 7.0% necrosis, indicating that some of the necrotic cells in the treatment groups could be attributed to procedural factors rather than compound-specific toxicity.

With the compound at IC_50_ concentration, HT29 cells showed 2.05 ± 0.25% early apoptosis, 16.95 ± 2.85% late apoptosis, and 12.2 ± 2.1% necrosis. After correcting for background necrosis, actual compound-induced necrosis appears minimal. These findings indicate that the anticancer activity of compound **5h** primarily occurs via the induction of apoptosis rather than through necrosis. This supports its potential as a selective and safer anticancer agent and supports the findings from cytotoxicity and enzyme inhibition assays.

### 3.4 Computational in Silico Studies

#### 3.4.1 Molecular Docking

The interaction between sorafenib and the most active compounds with the enzyme VEGFR-2 are illustrated in Figures 6–13. Interaction types for compounds **5c**, **5h**, **5j** and sorafenib are shown in Table 4.

**Figure 6:**
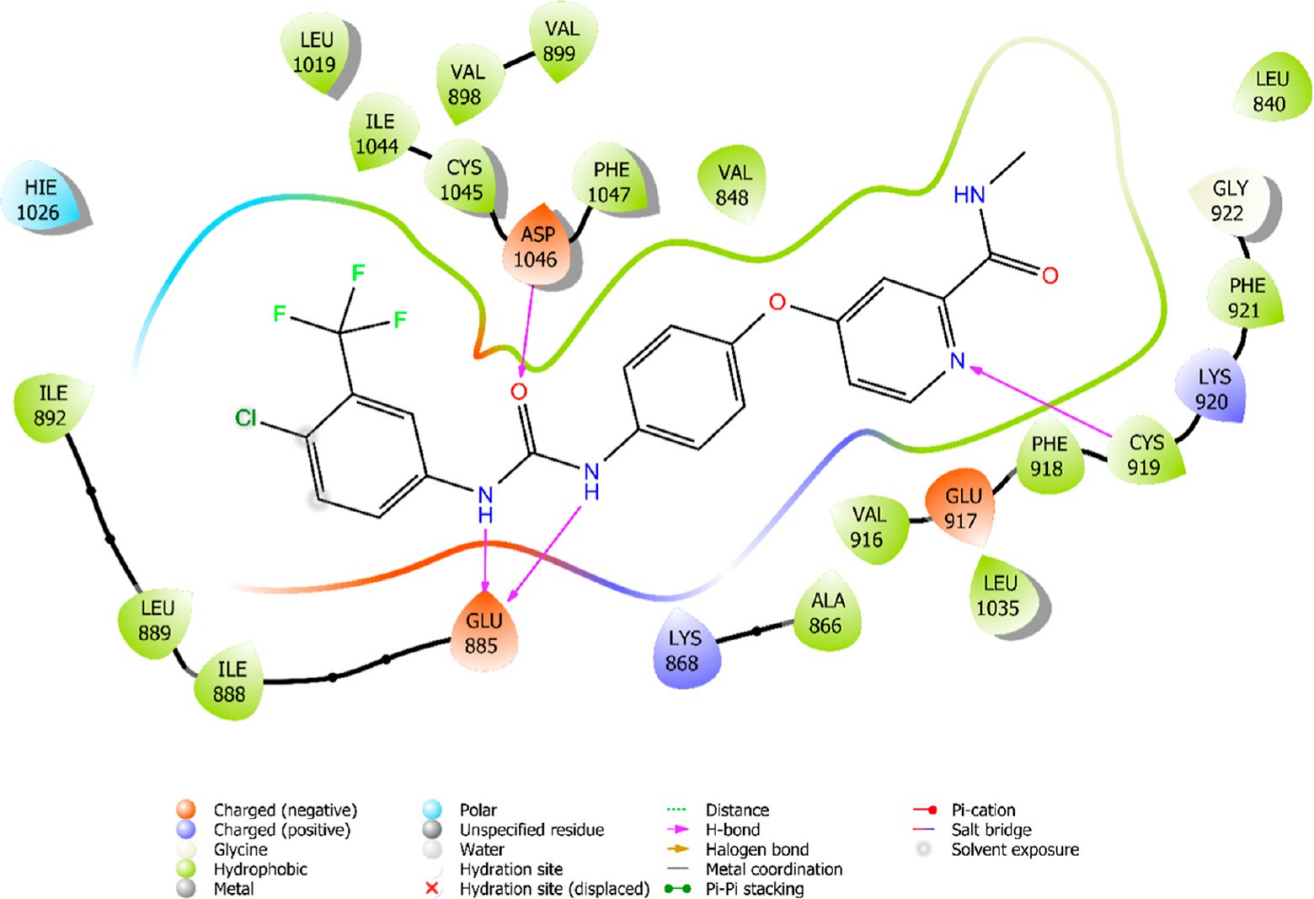
2D interaction diagram of sorafenib within the VEGFR-2 enzyme’s active site (PDB ID: 4ASD).

**7:**
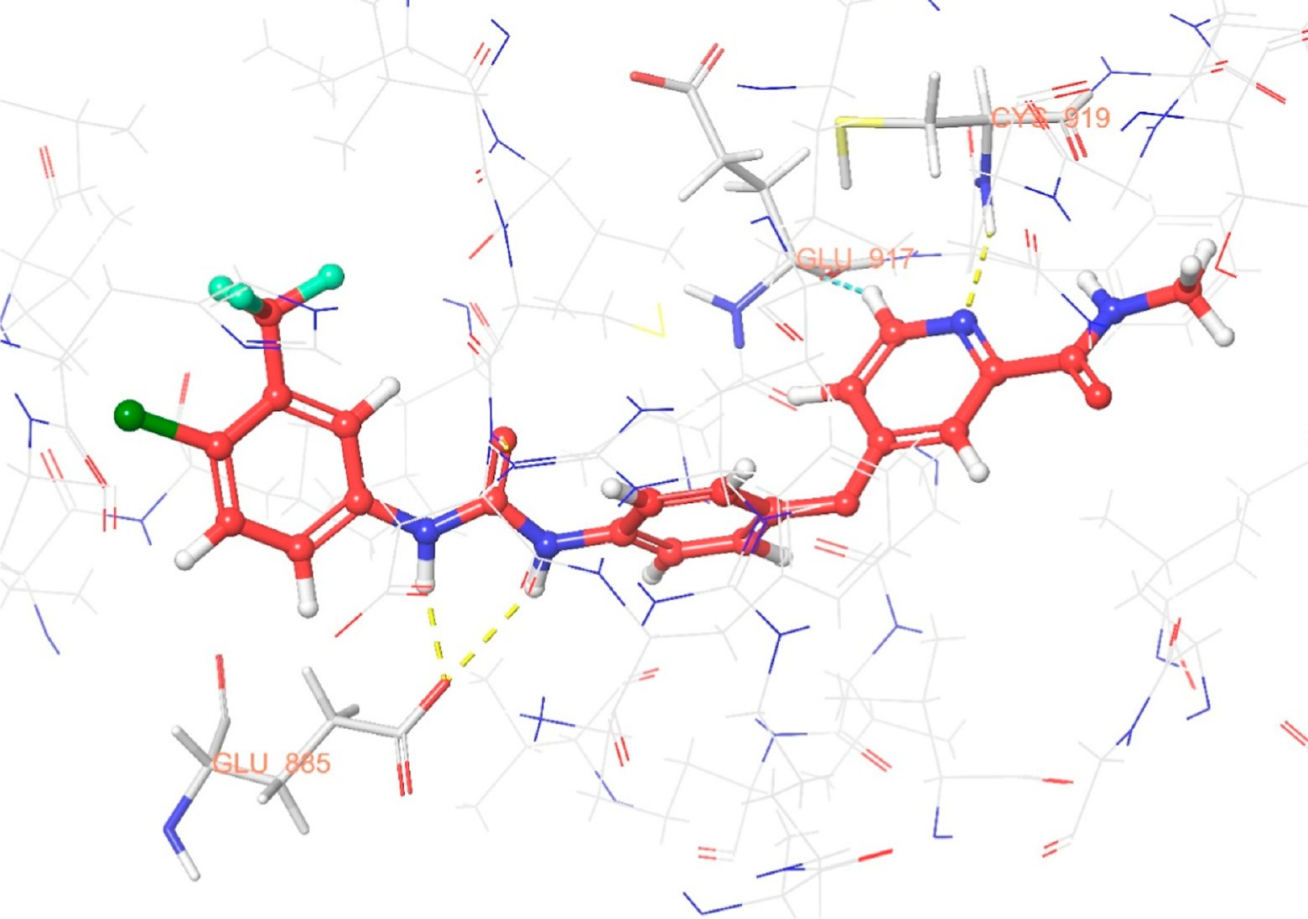
3D interaction of sorafenib within the VEGFR-2 enzyme’s active site (PDB ID: 4ASD).

**8:**
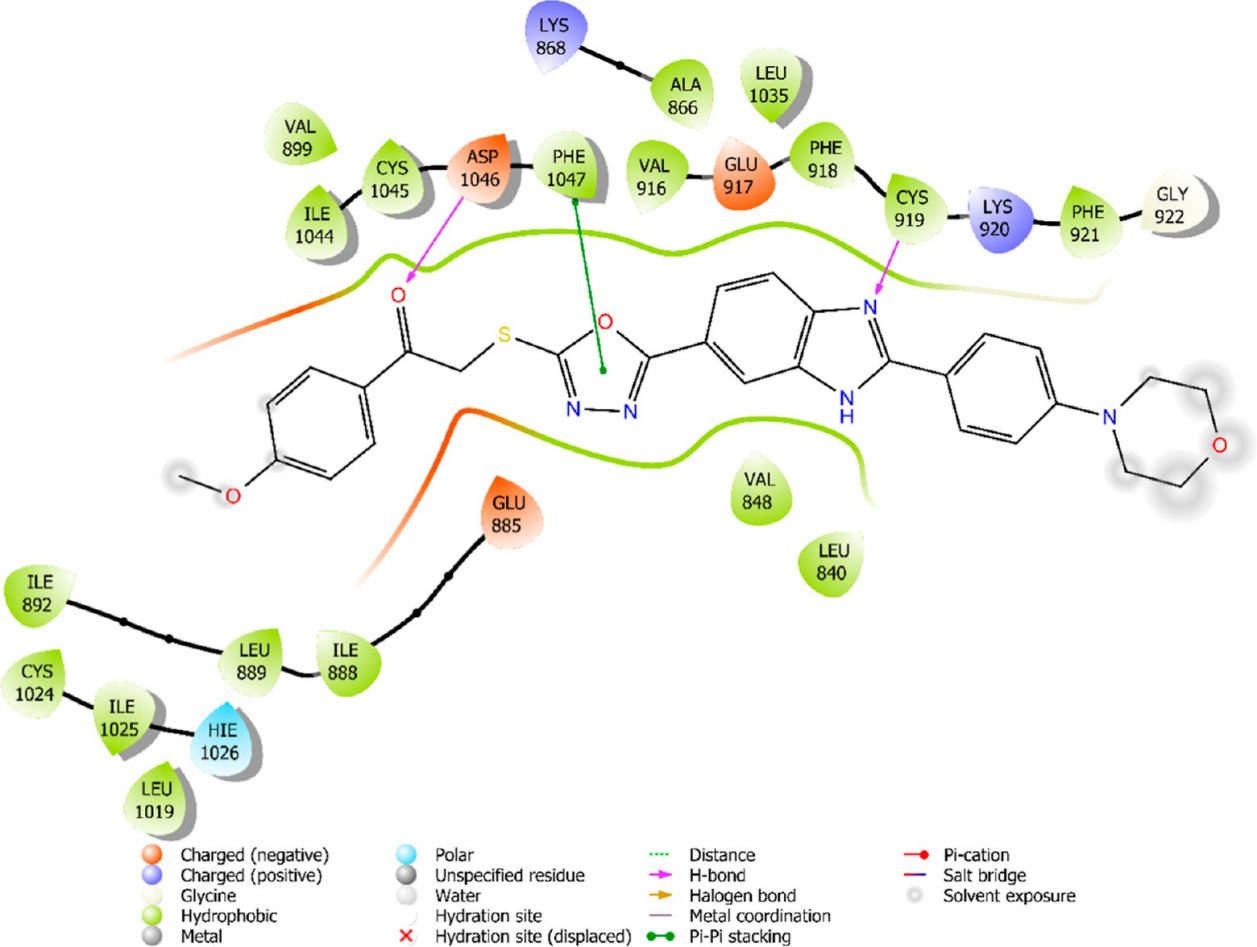
2D interaction diagram of compound **5c** within the VEGFR-2 enzyme’s active site (PDB ID: 4ASD).

**9:**
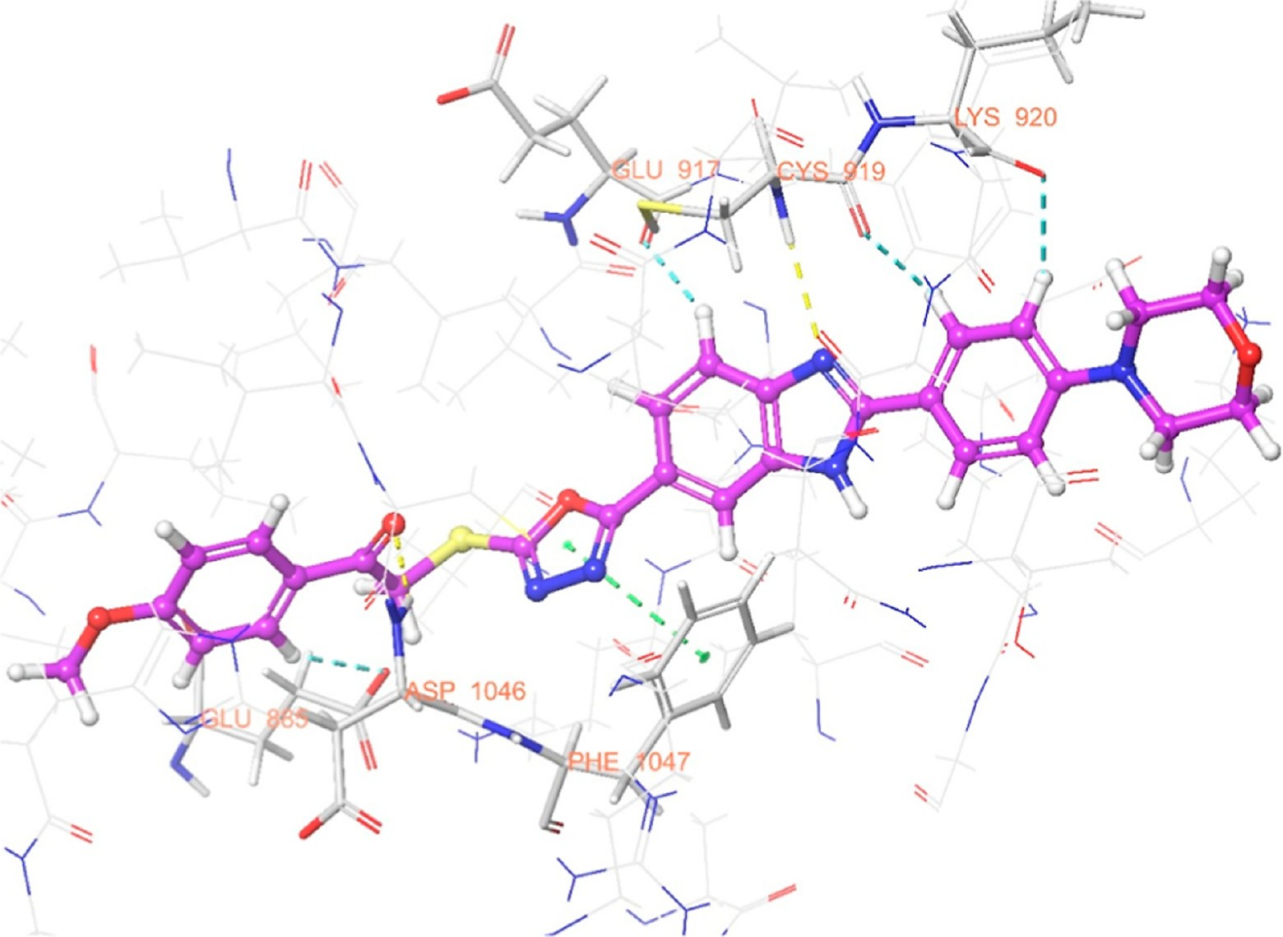
3D interaction diagram of compound **5c** within the VEGFR-2 enzyme’s active site (PDB ID: 4ASD).

**10:**
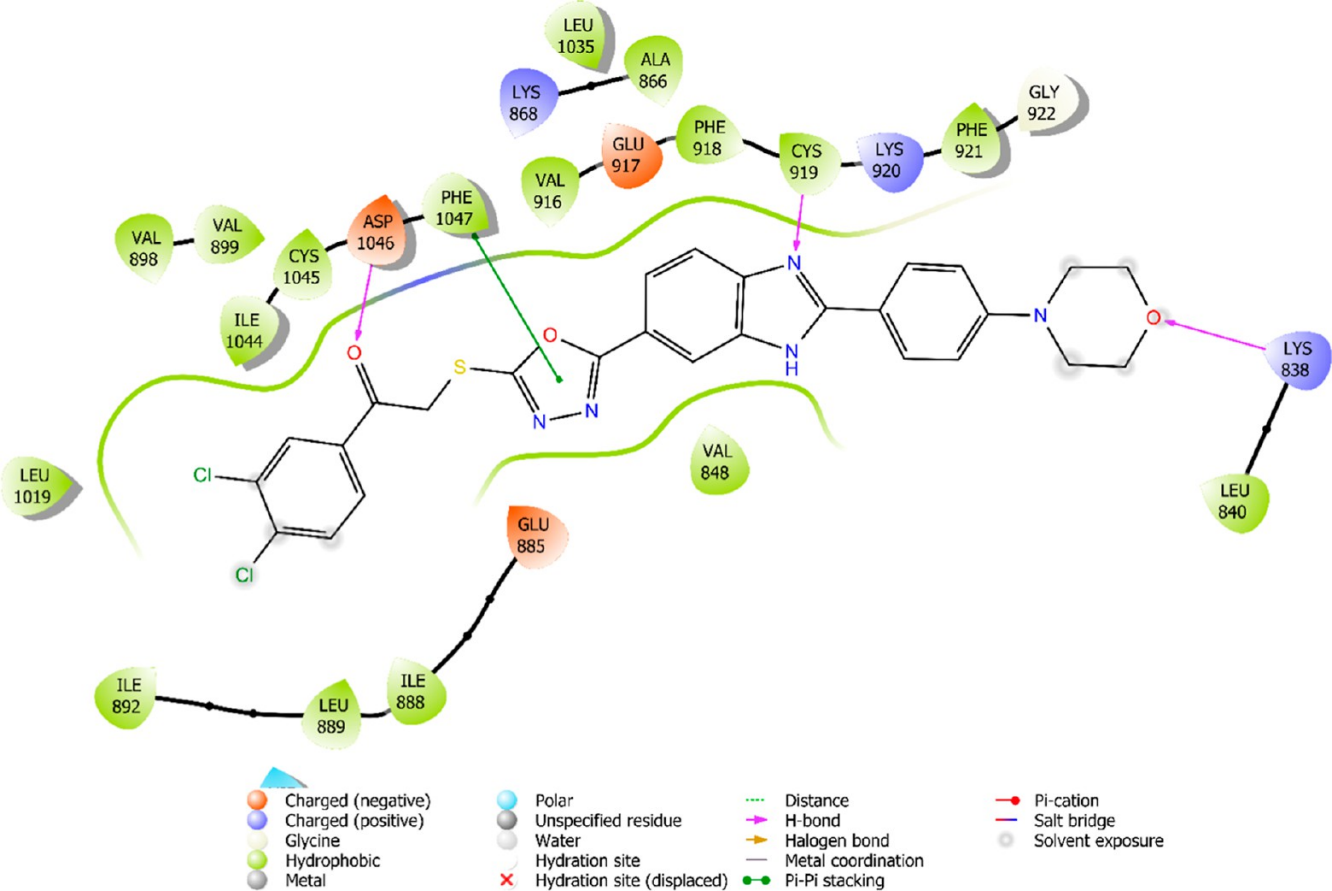
2D interaction diagram of compound **5h** within the VEGFR-2 enzyme’s active site (PDB ID: 4ASD).

**11:**
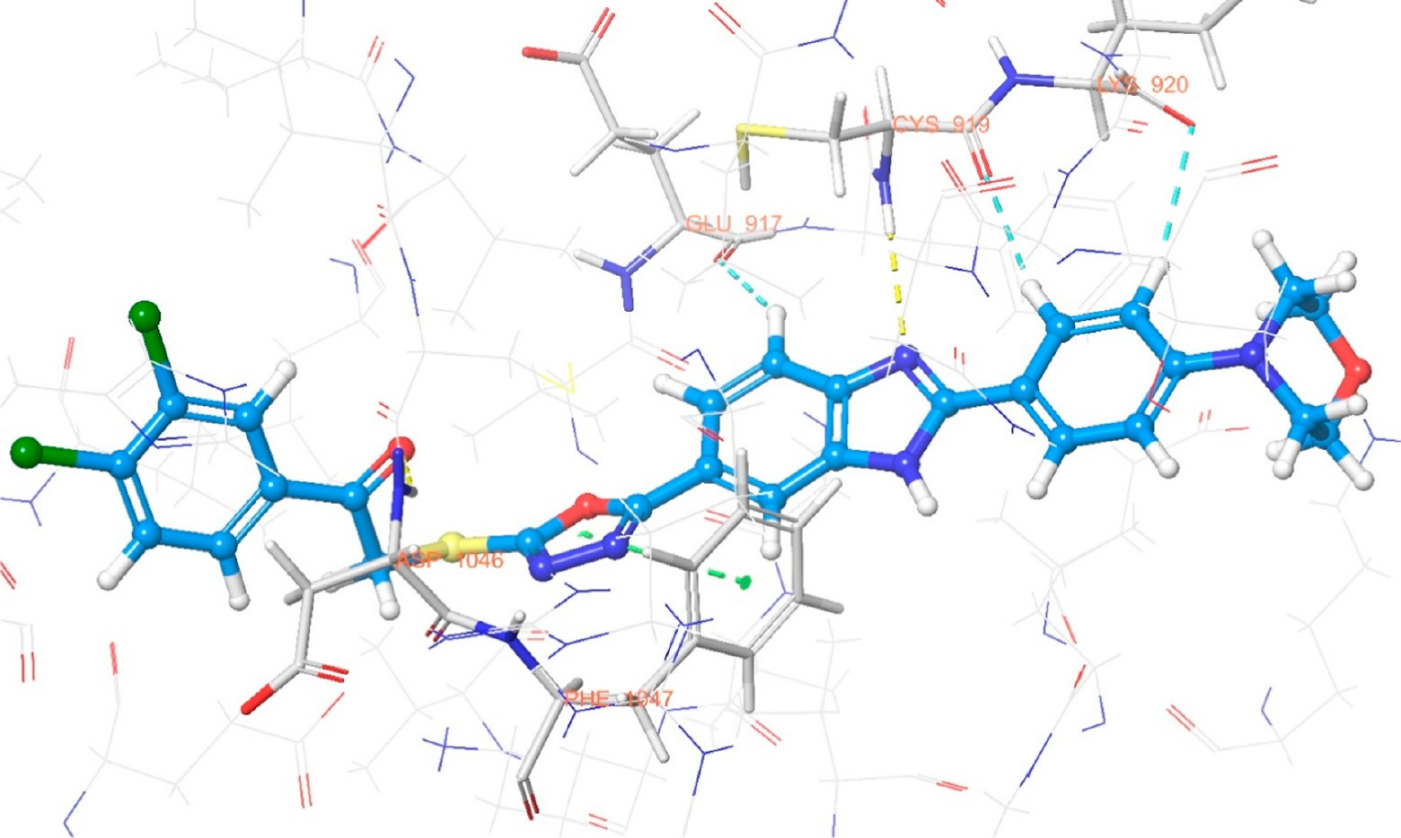
3D interaction diagram of compound **5h** within the VEGFR-2 enzyme’s active site (PDB ID: 4ASD).

**12:**
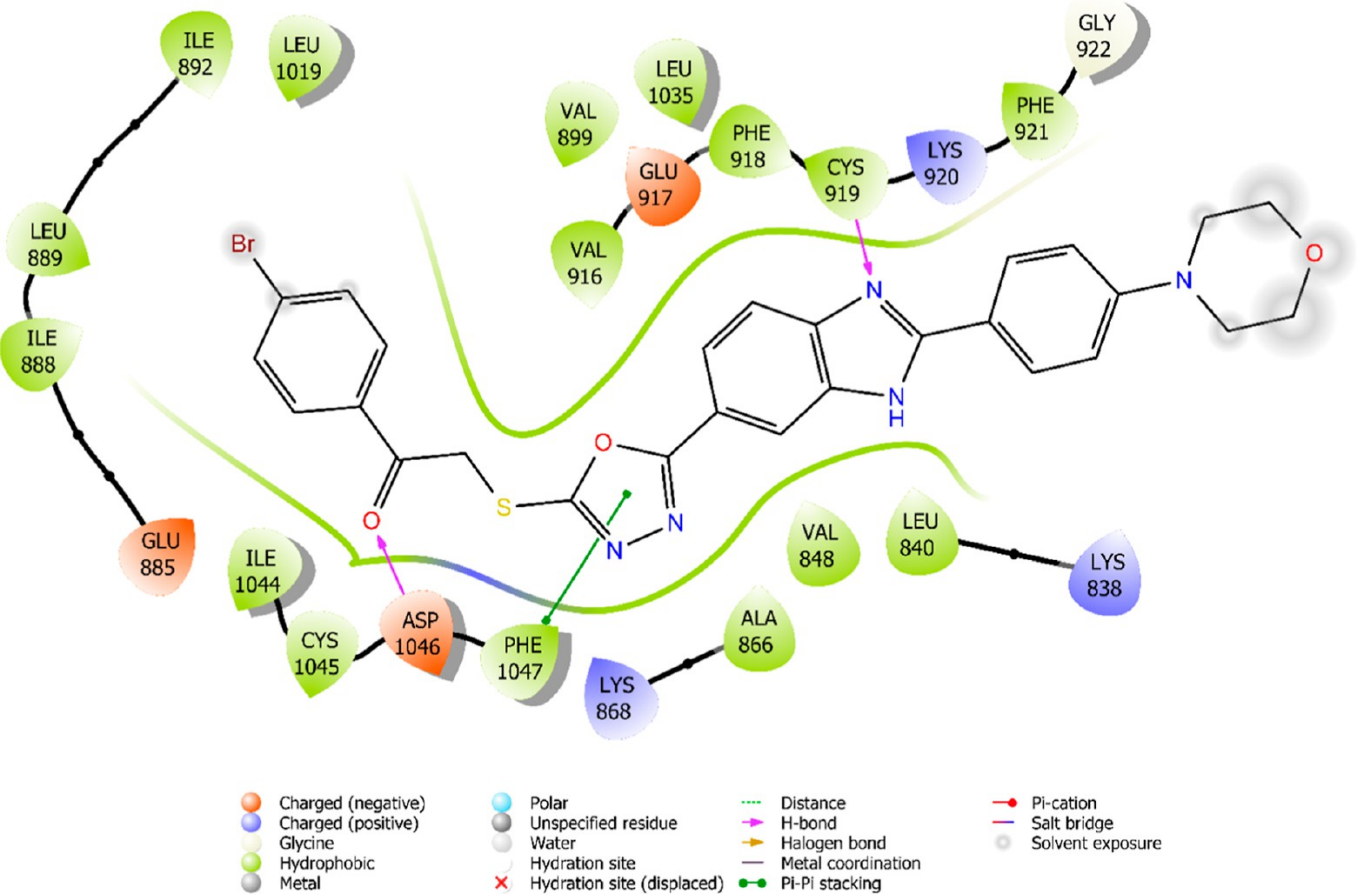
2D interaction diagram of compound **5j** within the VEGFR-2 enzyme’s active site (PDB ID: 4ASD).

**Figure 13:**
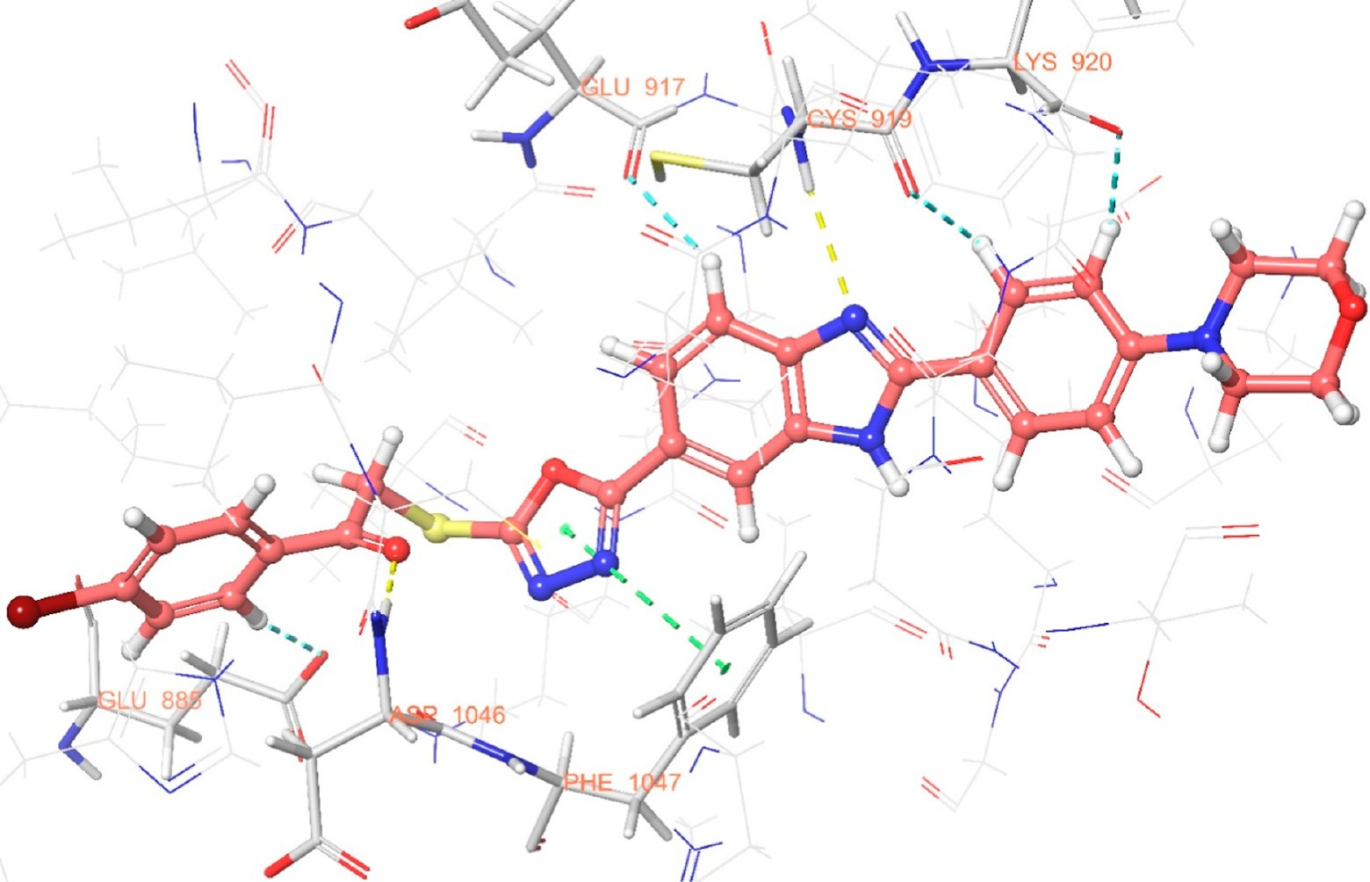
3D interaction diagram of compound **5j** within the VEGFR-2 enzyme’s active site (PDB ID: 4ASD).

**Table 4:** Interaction Types with VEGFR-2 Enzyme of Compounds **5c**, **5h**, **5j** and Sorafenib

comp	docking score (kcal/mol)	RMSD (max) (Å)	Pi–pi interactions	hydrogen bonds	aromatic hydrogen bonds
**5c**	–10.350	2.7	phenyl ring of Phe1047	amine gr. of Cys919	carbonyl gr. of Glu917
				amine gr. of Asp1046	carbonyl gr. of Cys919
					carbonyl gr. of Lys920
					hydroxy group of Glu885
**5h**	–10.400	2.7	phenyl ring of Phe1047	amine gr. of Cys919	carbonyl gr. of Glu917
				amine gr. of Asp1046	carbonyl gr. of Cys919
				amine gr. of Lys838	carbonyl gr. of Lys920
**5j**	–10.953	2.7	phenyl ring of Phe1047	amine gr. of Cys919	carbonyl gr. of Glu917
				amine gr. of Asp1046	carbonyl gr. of Cys919
					carbonyl gr. of Lys920
					hydroxy gr. of Glu885
**Sorafenib**	–12.011	2.0		amine gr. of Cys919	carbonyl gr. of Glu917
				hydroxy group of Glu885	

Results revealed that compounds **5c**, **5h**, and **5j** exhibit similar binding interactions as sorafenib at the VEGFR-2 enzyme’s active site. Specifically, their carbonyl groups form hydrogen bonds with the amino group of Asp1046, stabilizing the interaction. Additionally, a π–π stacking interaction occurs between the oxadiazole rings of compounds and phenyl rings of Phe1047, contributing to the overall binding affinity. Furthermore, the nitrogen atom of the benzimidazole ring establishes a hydrogen bond with the amino group of Cys919, further reinforcing molecular interaction. Notably, compound **5h** forms an additional hydrogen bond between the oxygen of its morpholine ring and Lys838, which may further enhance its binding stability. These interactions suggest a strong binding mode, which may enhance the inhibitory potential of these compounds.

#### 3.4.2 Molecular Dynamics Simulations

Molecular docking studies provide important information about the binding positions and interactions of ligands in the active site of an enzyme. These studies produce static snapshots that reveal potential interaction points that are important for understanding ligand-enzyme recognition. However, while these docking results are useful, they only represent the initial interaction and do not account for the dynamics of the system over time. This is important because the true activity of a ligand is determined not only by how well it fits into the active site at a given time, but also by its ability to maintain stable binding and interactions throughout the inhibition process.

To address this, dynamic simulations are essential because they reflect the true behavior of the ligand-enzyme complex over a longer period. These simulations reveal how the ligand behaves in the active site, its stability, binding time, and interactions with key amino acids. Therefore, dynamic studies are a more accurate representation of how a ligand will perform in a biological environment and highlight its potential for sustained inhibition.

Root mean square deviation (RMSD) and root mean square fluctuation (RMSF) are important parameters for evaluating the stability and flexibility of the ligand-enzyme complex during dynamic simulations.

RMSD measures the overall structural deviation of the ligand-enzyme complex over time and provides an indication of the stability of the binding position. A higher RMSD value (usually above 3 Å) indicates that the ligand has moved significantly from its initial docking position and is not stably bound in the active site of the enzyme. In contrast, a lower RMSD indicates that the ligand is well positioned and demonstrates stable binding. Therefore, RMSD is an important metric to determine whether the initial docking position has evolved into a stable, long–term interaction.

RMSF measures the fluctuations of individual atoms or residues during the simulation and provides insight into the flexibility of the ligand and the enzyme. For a ligand to be considered stable in the active site, the RMSF values of the interacting residues should be low, typically below 1 Å. Higher RMSF values may indicate that the ligand is not tightly bound or that it causes significant movement in the active site of the enzyme, which may compromise its inhibitory potential.

The RMSD, RMSF, Rg and SASA values (Figures 14–18) for compounds **5c**, **5h**, and **5j** are summarized in Table 5. The RMSD values for these compounds were found to be within an acceptable range, indicating that they remained stably bound within the enzyme’s active site throughout the simulation. Notably, none of the compounds showed RMSD values above the 3 Å threshold, indicating that the binding positions did not undergo significant fluctuations.

**Figure 14:**
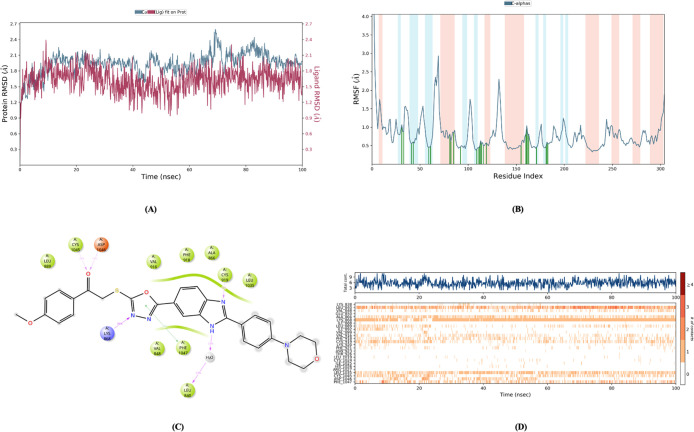
Molecular dynamic results of compound **5c**.

**Figure 15:**
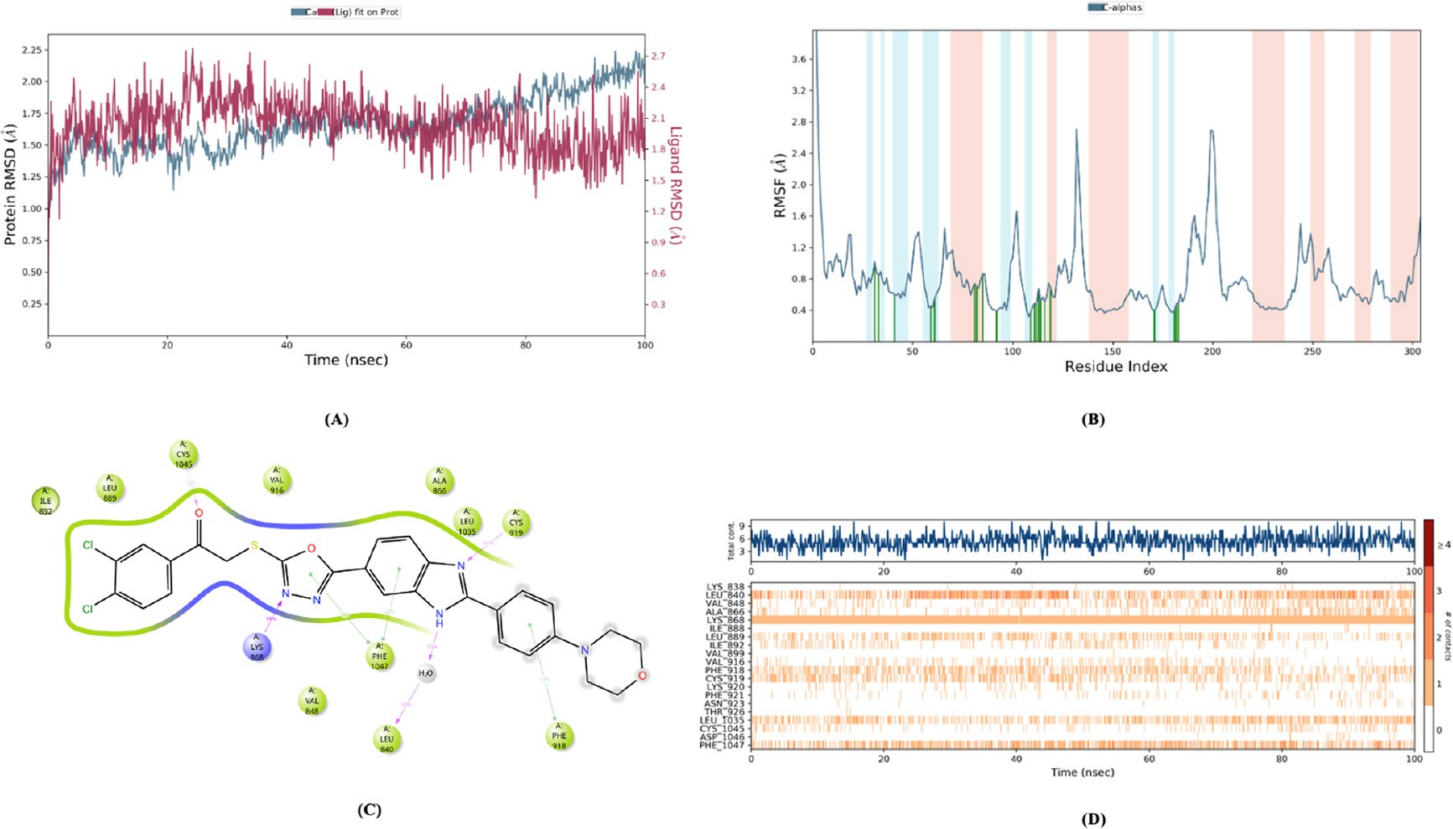
Molecular dynamic results of compound **5h**.

**Figure 16:**
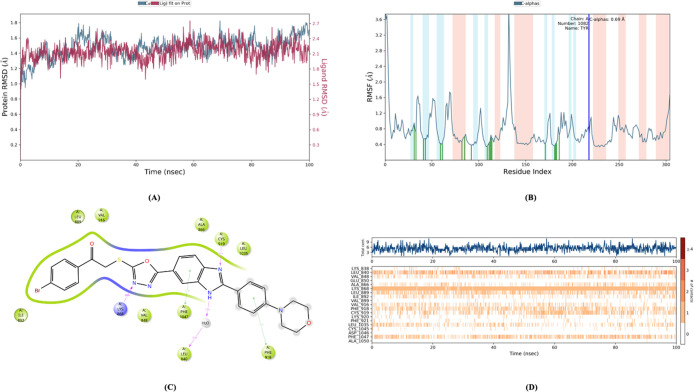
Molecular dynamic results of compound **5j**.

**Figure 17:**
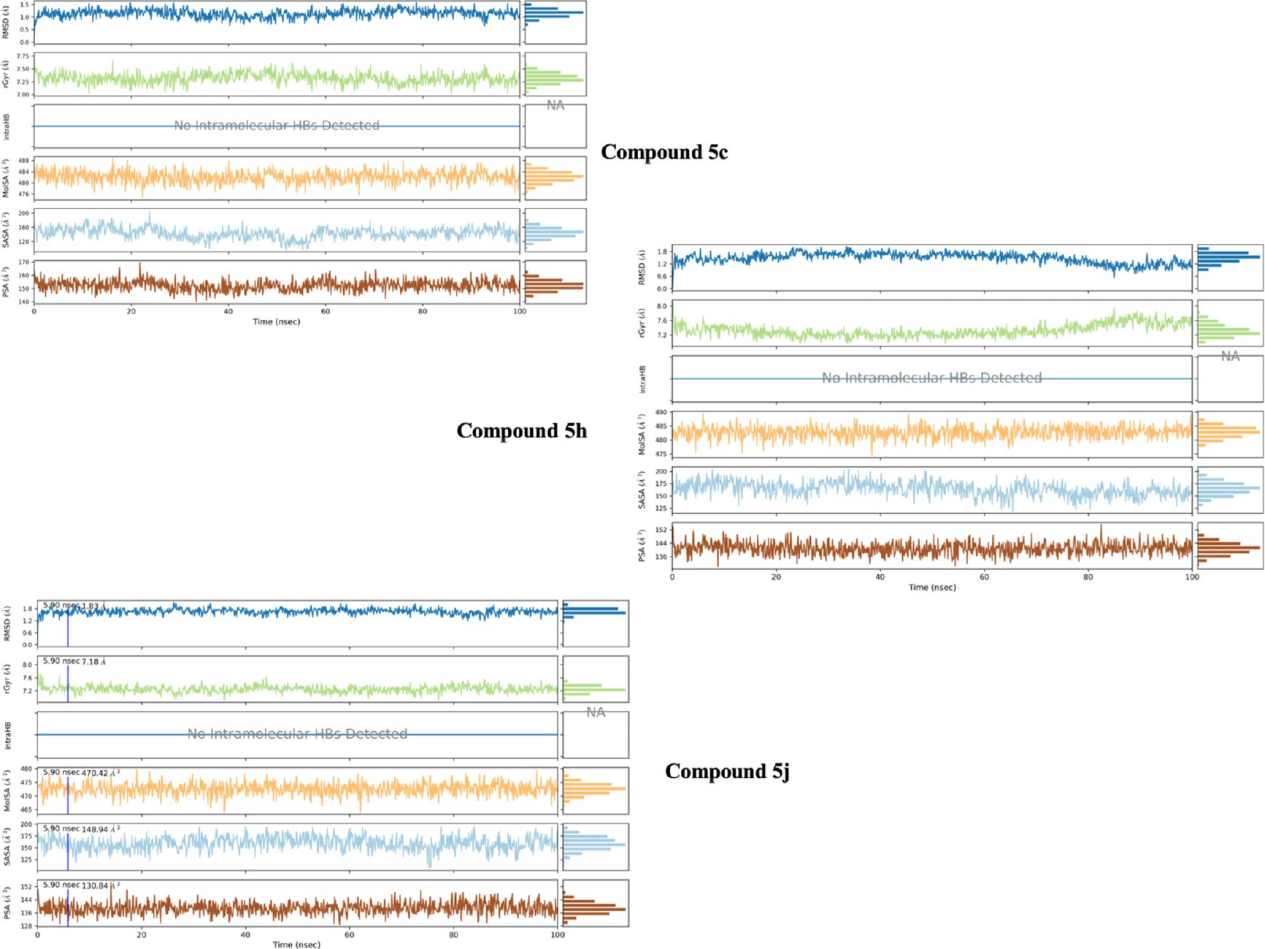
Metric radius of gyration (Rg) & (SASA) of compounds **5c**, **5h** and **5j**.

**Figure 18:**
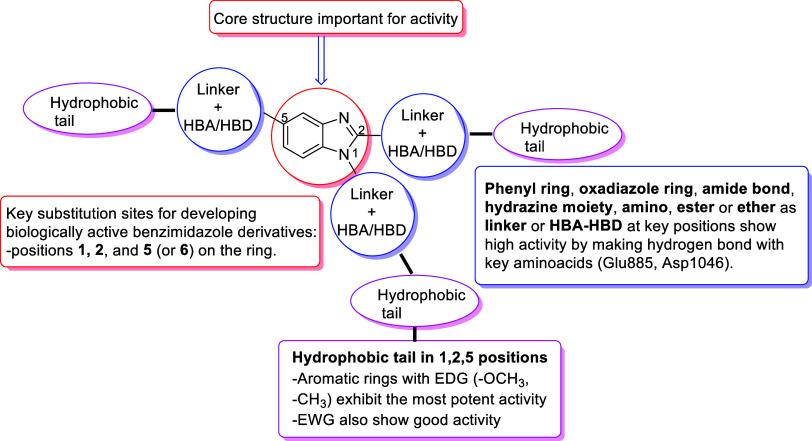
Structure–activity relationship of benzimidazole derivatives as effective VEGFR-2 inhibitors.

**Table 5:** RMSD, RMSF, Rg and SASA Parameters and Aromatic Hydrogen Bonds for Compounds **5c**, **5h** and **5j**

Comp	RMSD	Rg (Å)	SASA (Å^2^)	RMSF
**5c**	2.7 Å	7.00–7.75	120–200	Lys838 (1.06 Å), Leu840 (0.88 Å), Val848 (0.55 Å), Ala851 (0.57 Å), Ala866 (0.46 Å), Lys868 (0.48 Å), Ile888 (0.81 Å), Leu889 (0.62 Å), Ile892 (0.85 Å), Val899 (0.46 Å), Val916 (0.40 Å), Phe918 (0.44 Å), Cys919 (0.48 Å), Lys920 (0.63 Å), Gly922 (0.58 Å), Leu924 (0.50 Å), Thr926 (0.57 Å), Ala1020 (0.63 Å), Lys1023 (0.75 Å), Ile1025 (1.04 Å), Arg1027 (0.81 Å), Leu1036 (0.48 Å), Cys1045 (0.49 Å), Asp1046 (0.60 Å), Phe1047 (0.55 Å)
**5h**	2.25 Å	7.20–7.80	125–200	Gly837 (0.86 Å), Leu840 (0.83 Å), Val848 (0.59 Å), Ala866 (0.46 Å), Lys868 (0.55 Å), Ile888 (0.74 Å), Leu889 (0.70 Å), Ile892 (0.86 Å), Asn900 (0.42 Å), Ile915 (0.32 Å), Phe918 (0.49 Å), Cys919 (0.48 Å), Lys920 (0.67 Å), Phe921 (0.51 Å), Leu924 (0.58 Å), Thr926 (0.68 Å), Leu1035 (0.40 Å), Cys1045 (0.39 Å), Asp1046 (0.49 Å), Phe1047 (0.48 Å), Gly1048 (0.61 Å)
**5j**	1.8 Å	7.20–7.80	120–200	Gly837 (0.84 Å), Leu840 (0.71 Å), Val848 (0.53 Å), Glu850 (0.55 Å), Ala866 (0.43 Å), Lys868 (0.43 Å), Leu889 (0.47 Å), Ile892 (0.60 Å), Val899 (0.37 Å), Val916 (0.36 Å), Phe918 (0.44 Å), Cys919 (0.47 Å), Lys920 (0.64 Å), Gly922 (0.55 Å), Leu1035 (0.42 Å), Cys1045 (0.36 Å), Asp1046 (0.41 Å), Phe1047 (0.45 Å), Ala1050 (0.63 Å)

Regarding RMSF, most of the key amino acid interaction sites for all three compounds had values below 1 Å. This indicates that these compounds maintained low levels of fluctuations in the active site of the enzyme, further strengthening the idea that their binding is stable and consistent.

These dynamic simulation results provide strong evidence that compounds **5c**, **5h**, and **5j** not only bind well to the active site of the enzyme initially but also exhibit stability and minimal fluctuations throughout the simulation period. This suggests that these compounds likely have sustained inhibitory effects and are promising candidates for further development in the context of enzyme inhibition.

Figures 14–17 display the outcomes of 100 ns molecular dynamics simulations for the compounds and the complexes formed by PDB ID: 4ASD. Specifically, Figures 14A, 15A, and 16A display the RMSD (Root Mean Square Deviation) plots, while Figures 14B, 15B, and 16B show the RMSF (Root Mean Square Fluctuation) plots. Figures 14C, 15C, and 16C provide two-dimensional visual representations of amino acid interactions, and Figures 14D, 15D, and 16D depict the temporal continuity of amino acid interactions throughout the simulation.

Among the key amino acids involved in VEGFR-2 inhibition, Glu885, Cys919, and Asp1046 were identified as important interaction sites. As is known, none of the compounds interacted with Glu885. However, the amine group of Cys919 formed hydrogen bonds with the benzimidazole nitrogen of compounds **5c**, **5h**, and **5j**. For compound **5h**, an additional aromatic hydrogen bond was observed between the carbonyl functional group of Cys919 and the phenyl ring attached to the benzimidazole (as seen in the video). This aromatic hydrogen bond is only visible in the video demonstration.

Furthermore, compounds **5h** and **5j** form continuous aromatic hydrogen bonds with Asp1046 through their substituted phenyl rings. Compound **5c** also forms a similar aromatic hydrogen bond, but this interaction occurs less frequently during the simulation.

Considering the additional aromatic hydrogen bonds with Cys919 and the continuous aromatic hydrogen bond with Asp1046, it can be concluded that compound **5h** may exhibit higher VEGFR-2 enzyme inhibition potential compared to the other compounds in the series.

### 3.5 Structure–Activity Relationship

Structural analysis showed that the presence of chlorine substituents at both positions 3 and 4 of the phenyl ring in compound **5h** significantly increased its binding affinity within the ATP-binding pocket of VEGFR-2, contributing to its potent inhibitory activity. Notably, compound **5h** exhibited approximately 33-fold greater cytotoxic activity compared to compound **5e**, which contains only one chlorine atom at position 4. This significant difference highlights the critical role of the additional chlorine substituent at position 3 in enhancing biological activity.

Furthermore, compounds **5j** and **5c** also showed significant cytotoxicity against HT-29 cells, with IC_50_ values of 9.657 ± 0.149 μM and 17.750 ± 1.768 μM, respectively. Compound **5c**, which bears a methoxy functional group at position 4 of the phenyl ring, may exhibit enhanced cytotoxicity either due to the intrinsic electron-donating effect of the methoxy group or via *O*-demethylation, potentially forming a biologically active hydroxylated metabolite. Compound **5j**, which bears a bromine atom at position 4, also showed enhanced activity, suggesting that halogen substitution at this position positively contributes to cytotoxic effects. On the other hand, with respect to VEGFR-2 inhibition, the bromine-substituted derivative exhibits greater activity. Even though bromine and methoxy groups do not directly participate in specific interactions with the protein, they influence the overall ligand conformation and, consequently, the strength of interactions within the binding pocket. In particular, the bromine-containing compound adopts a configuration that places the benzimidazole ring in a more stable and closer proximity to Cys919, resulting in a stronger interaction with this key residue compared to the methoxy-substituted analog. This interaction is almost continuous in the 3,4-dichlorinated derivative. This means that derivatives containing chlorine in both the third and fourth positions provide this stability better.

Overall, the SAR findings highlight the importance of halogen substituents, particularly chlorine at both positions 3 and 4, in improving the biological activity of the synthesized derivatives, most likely through enhanced interactions within the VEGFR-2 enzyme’s active site.

## 4 Conclusions

In the present study, a new series of morpholine-benzimidazole-oxadiazole derivatives was successfully designed, synthesized and thoroughly characterized. Compounds were systematically evaluated for their anticancer properties, aiming to identify promising candidates with potent activity. Among the synthesized compounds, compound **5h** exhibited the highest cytotoxicity against HT-29 colon cancer cells (IC_50_ = 3.103 ± 0.979 μM) while maintaining selectivity over NIH3T3 normal cells (IC_50_ = 15.158 ± 0.987 μM, SI = 4.88). Notably, compound **5h** demonstrated approximately 33-fold higher activity than compound **5e**, suggesting that the additional chlorine substituent at position 4 of the phenyl ring plays a crucial role in enhancing biological activity.

The VEGFR-2 enzyme inhibition assay offered further validation of these derivatives’ promise as potent VEGFR-2 inhibitors. The most potent compound, **5h**, exhibited the strongest inhibition (IC_50_ = 0.049 ± 0.002 μM), comparable to the reference drug sorafenib (IC_50_ = 0.037 ± 0.001 μM). Additionally, compound **5j** (IC_50_ = 0.098 ± 0.011 μM) and compound **5c** (IC_50_ = 0.915 ± 0.027 μM) also displayed notable inhibitory effects. The structural analysis suggests that the chlorine substituents at both the 3- and 4-positions in the phenyl ring of the compound **5h** enhances its binding affinity within the ATP-binding pocket of VEGFR-2, contributing to its potent inhibitory activity.

Structural modifications also influenced cytotoxicity. Compound **5c**, containing a methoxy substituent at the phenyl ring’s 4-position, exhibited increased toxicity, likely due to its *O*-demethylation into a hydroxylated metabolite. Similarly, compound **5j**, with a bromine substituent at the 4-position, showed elevated toxicity. These findings highlight the impact of specific substituent effects on biological activity and toxicity profiles.

To further explore the mechanism of action, the apoptotic potential of compound **5h** was assessed using the Annexin V-FITC/PI dual staining method, revealing that 22.1% of HT-29 cells underwent apoptosis following treatment. This suggests that the anticancer effect of compound **5h** is primarily mediated through the apoptotic pathway rather than necrosis.

Additionally, in silico studies (molecular docking and molecular dynamics simulations) confirmed that compounds **5c**, **5h**, and **5j** not only bind effectively to the VEGFR-2 active site but also exhibit stability and minimal fluctuations throughout the simulation period. This suggests sustained inhibitory effects, reinforcing their potential as promising VEGFR-2 inhibitors for further development.

Overall, these findings indicate that the synthesized morpholine-benzimidazole-oxadiazole derivatives, particularly compound **5h**, hold significant promise as VEGFR-2 inhibitors with anticancer potential. Further preclinical studies are warranted to validate their therapeutic potential and optimize their pharmacological properties.

## Supplementary Material



## Notes

LC–MS spectra of compounds **1**, **2**, **4**. HRMS spectrum of compound **3**. ^1^H NMR, ^13^C NMR, LC–MS spectra of compounds **5a**-**5j**. IC_50_ graph of compounds **5c**, **5h**, **5j** for HT29, NIH3T3 cell lines and VEGFR-2 enzyme Anadolu University Project coded BGT-2023–2359 was used as a funding source for the supply of materials used in the cytotoxicity part of the study (PDF)
