# Supplementary material for: Targeting Cancer with New Morpholine-Benzimidazole-Oxadiazole Derivatives: Synthesis, Biological Evaluation, and Computational Insights
Source: ACS Omega. 2025 Jul 30;10(32):36134–53. doi: 10.1021/acsomega.5c03795 (PMC12368815; doi:10.1021/acsomega.5c03795)
Supplement: Supplementary file 1 [file ao5c03795_si_001.pdf]

Targeting Cancer with New Morpholine-Benzimidazole-Oxadiazole Derivatives: Synthesis, Biological Evaluation, and Computational Insights

Gresa Halimi Syla[1,2], Derya Osmaniye[1,3*], Büşra Korkut Çelikateş[4], Yusuf Özkay[1,3], Zafer Asım Kaplancıklı[1,5]

(1) Department of Pharmaceutical Chemistry, Faculty of Pharmacy, Anadolu University

(2) Institute of Graduate Education, Anadolu University

(3) Central Analysis Laboratory, Faculty of Pharmacy, Anadolu University

(4) Department of Pharmaceutical Toxicology, Faculty of Pharmacy, Anadolu University

(5) *Department of Pharmacy Services, Vocational School of Health Services, Bilecik Seyh Edebali University*

* Correspondence: dosmaniye@anadolu.edu.tr
Tel: +90- *Fax*: +90-222 335 05 80-3778

Address: Anadolu University, Faculty of Pharmacy, Department of Pharmaceutical Chemistry, 26470, Eskişehir, Turkey.

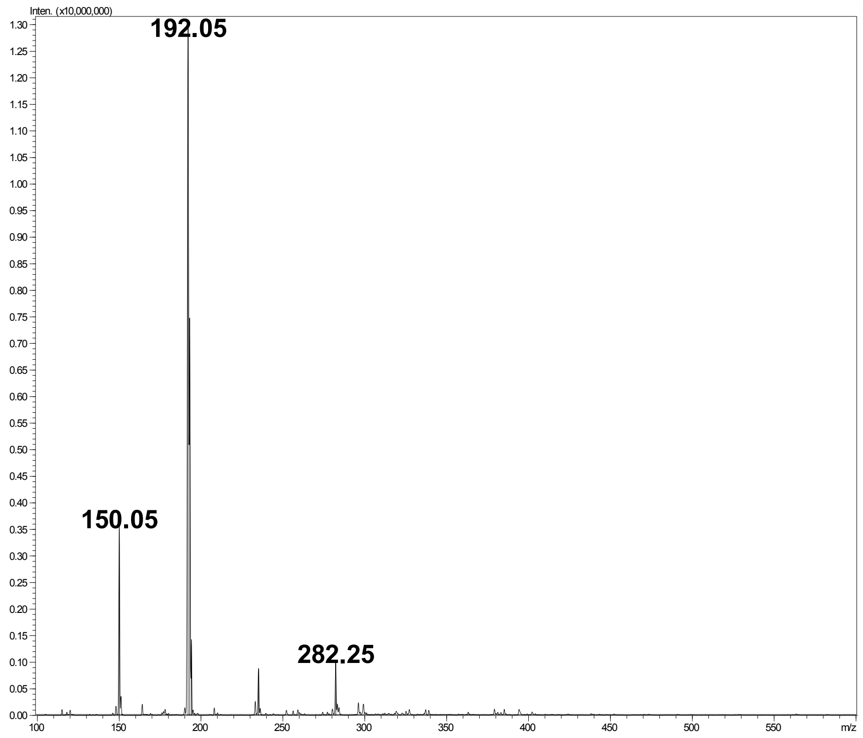

Figure S1. LC-MS spectral analysis of compound **1.**

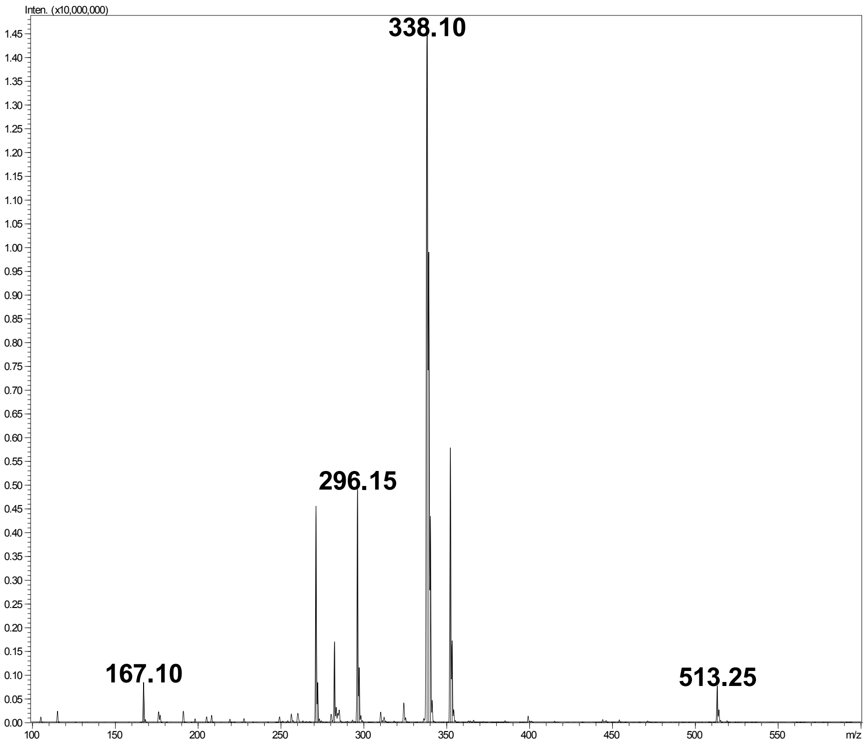

Figure S2. LC-MS spectral analysis of compound **2.**

Formula Predictor Report - GT-2_574.lcd

Data File: C:\LabSolutions\Data\Analiz\derya\GT-2_574.lcd

Elmt	Val.	Min	Max	Elmt	Val.	Min	Max	Elmt	Val.	Min	Max	Elmt	Val.	Min	Max	Use Adduct
H	1	10	33	O	2	0	5	S	2	0	0	Ru	2	0	0	H
C	4	10	32	F	1	0	0	Cl	1	0	0	Pd	2	0	0	Na
N	3	0	6	P	3	0	0	Br	1	0	0	I	3	0	0	

Error Margin (ppm):	5	DBE Range:	0.0 - 30.0	Electron Ions:	both
HC Ratio:	unlimited	Apply N Rule:	no	Use MSn Info:	yes
Max Isotopes:	3	Isotope RI (%):	1.00	Isotope Res:	9000
MSn Iso RI (%):	10.00	MSn Logic Mode:	AND	Max Results:	50

Event#: 1 MS(E+) Ret. Time : 2.907 Scan# : 437

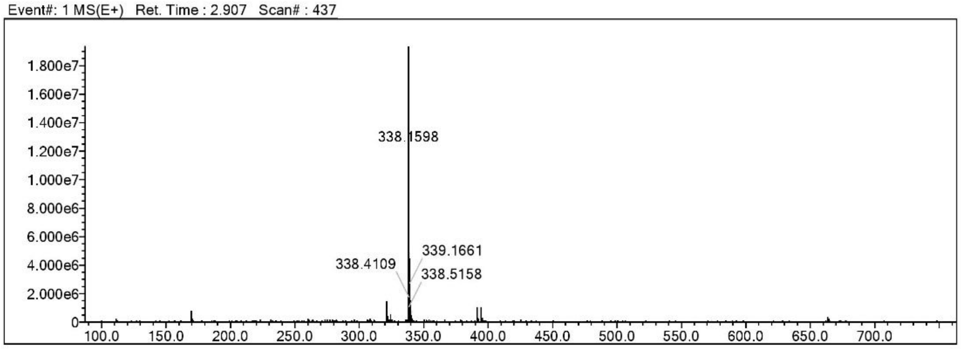

Measured region for 338.1598 m/z

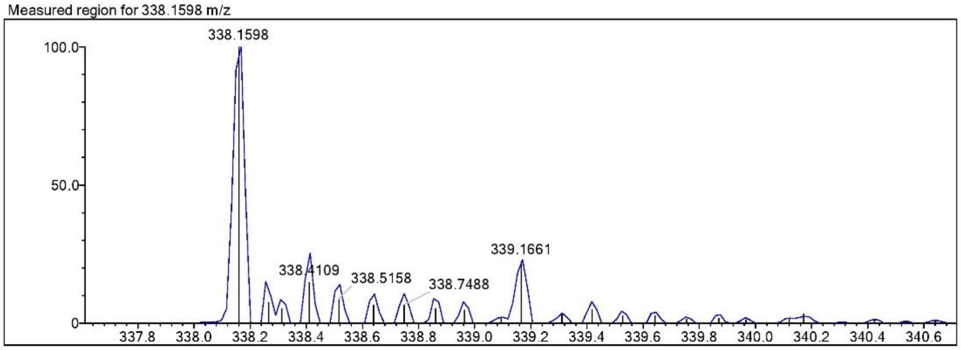

C18 H19 N5 O2 [M+H]+ : Predicted region for 338.1612 m/z

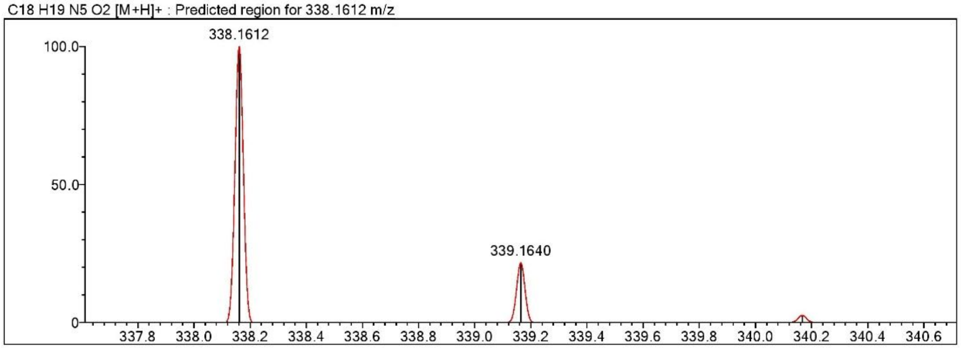

Rank	Score	Formula (M)	Ion	Meas. m/z	Pred. m/z	Df. (mDa)	Df. (ppm)	Iso	DBE
2	58.89	C18 H19 N5 O2	[M+H]+	338.1598	338.1612	-1.4	-4.14	63.91	12.0

Figure S3. HRMS spectral analysis of compound **3.**

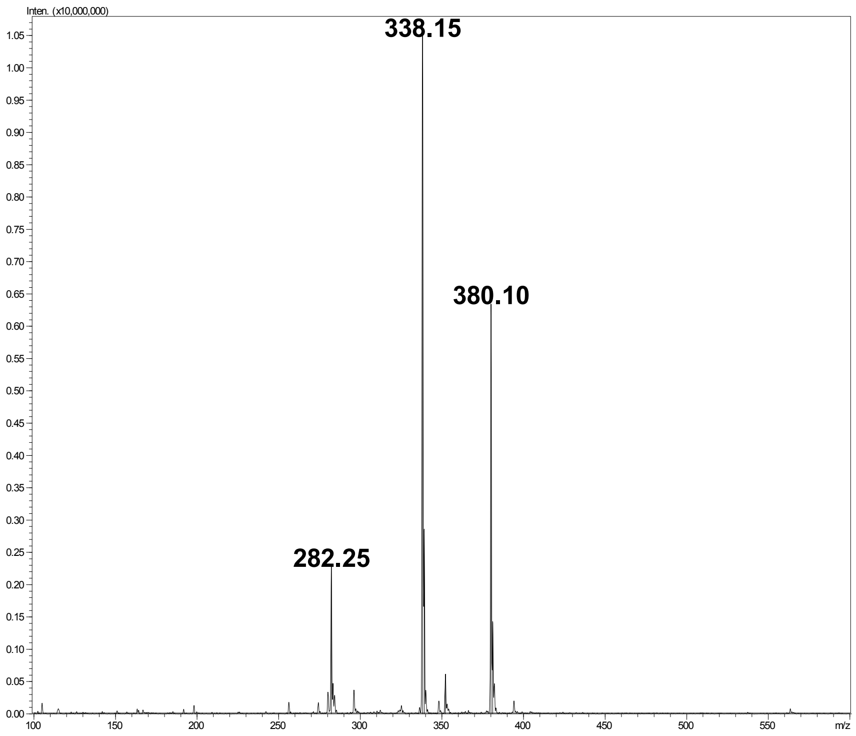

Figure S4. LC-MS spectral analysis of compound **4.**

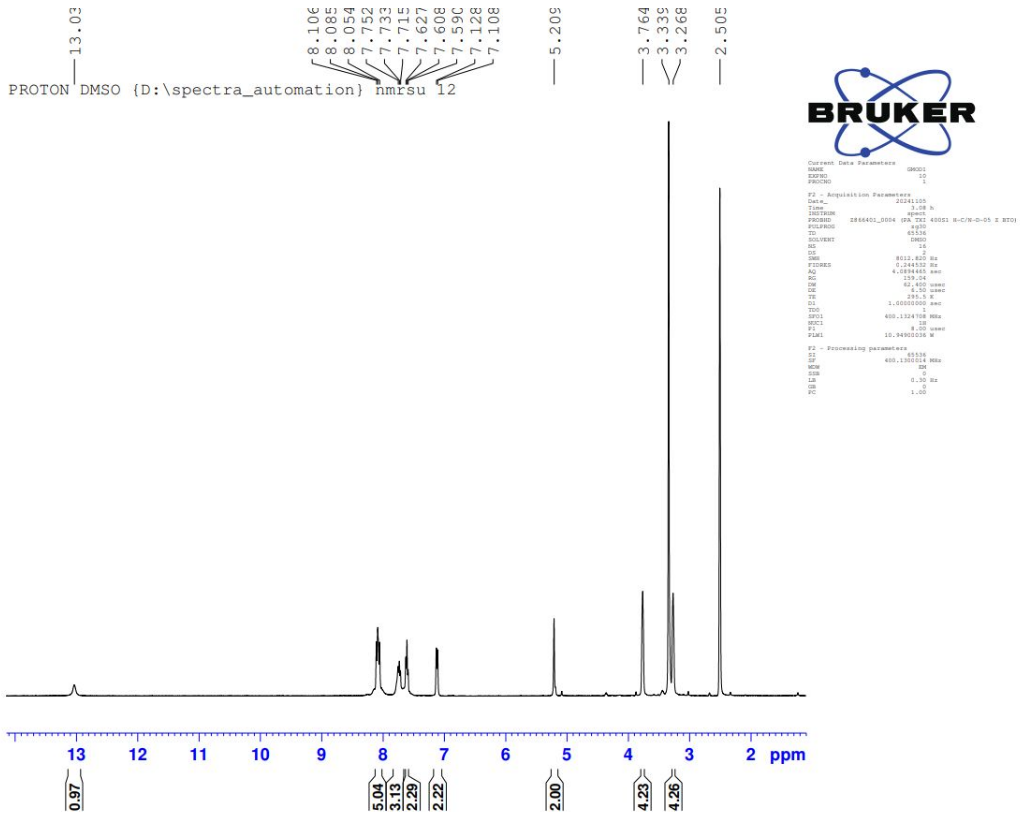

Figure S5. ^{1}H-NMR spectral analysis of compound **5a**.

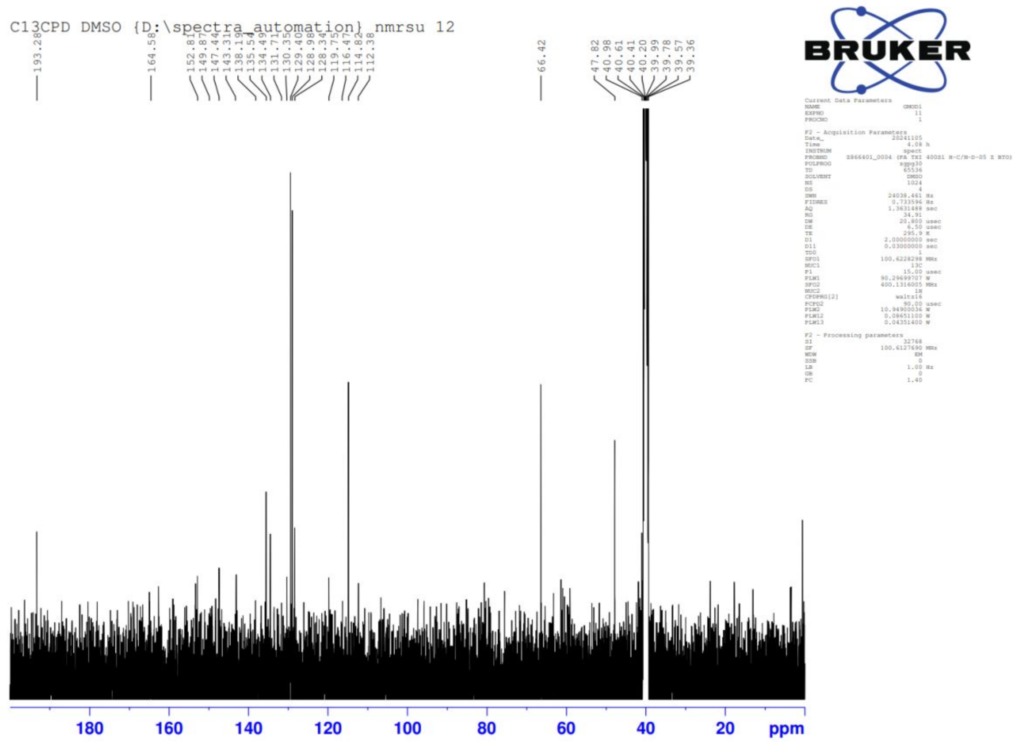

Figure S6. ^{13}C-NMR spectral analysis of compound **5a**.

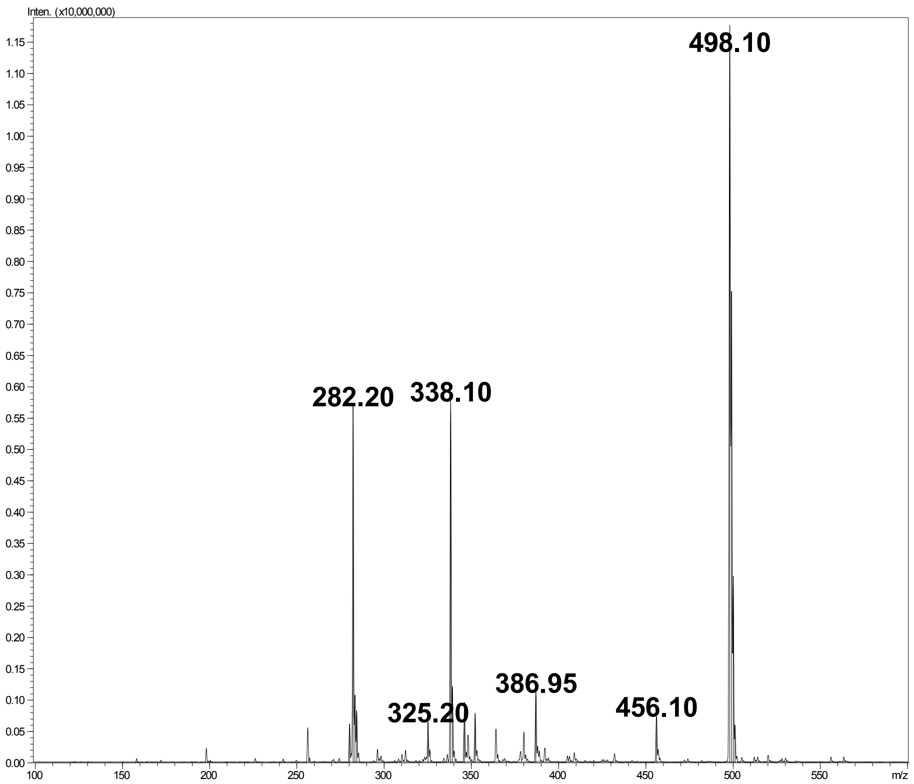

Figure S7. LC-MS spectral analysis of compound **5a**.

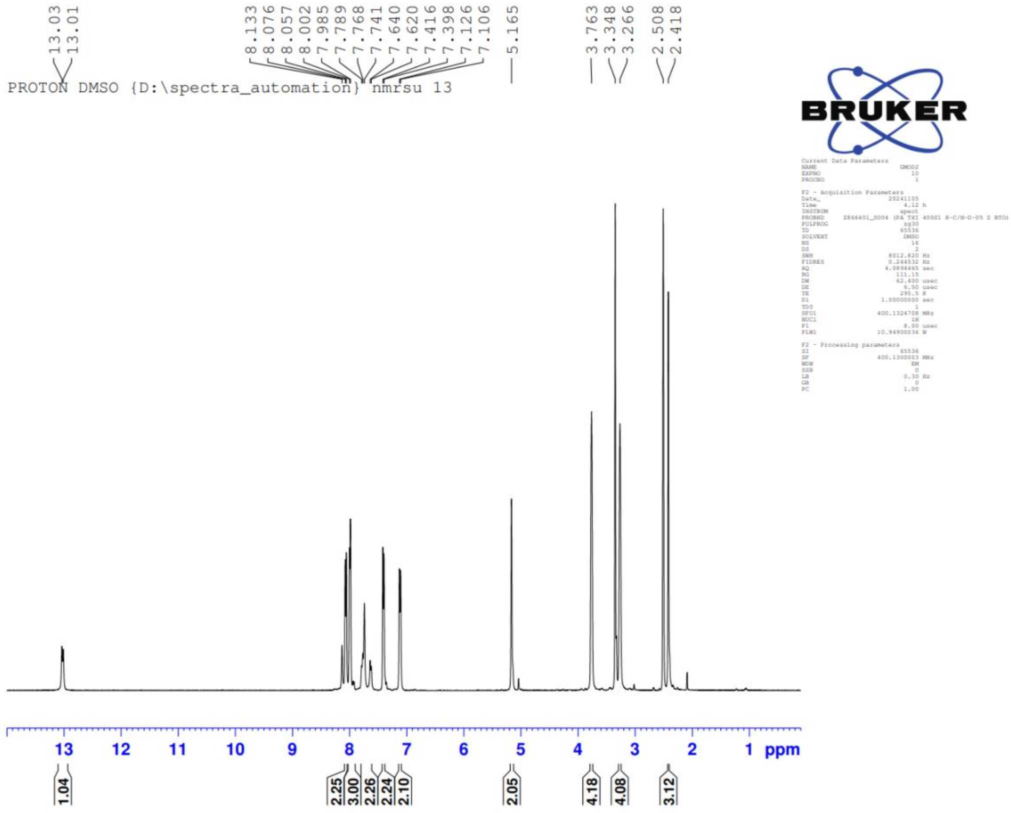

Figure S8. ^{1}H-NMR spectral analysis of compound **5b**.

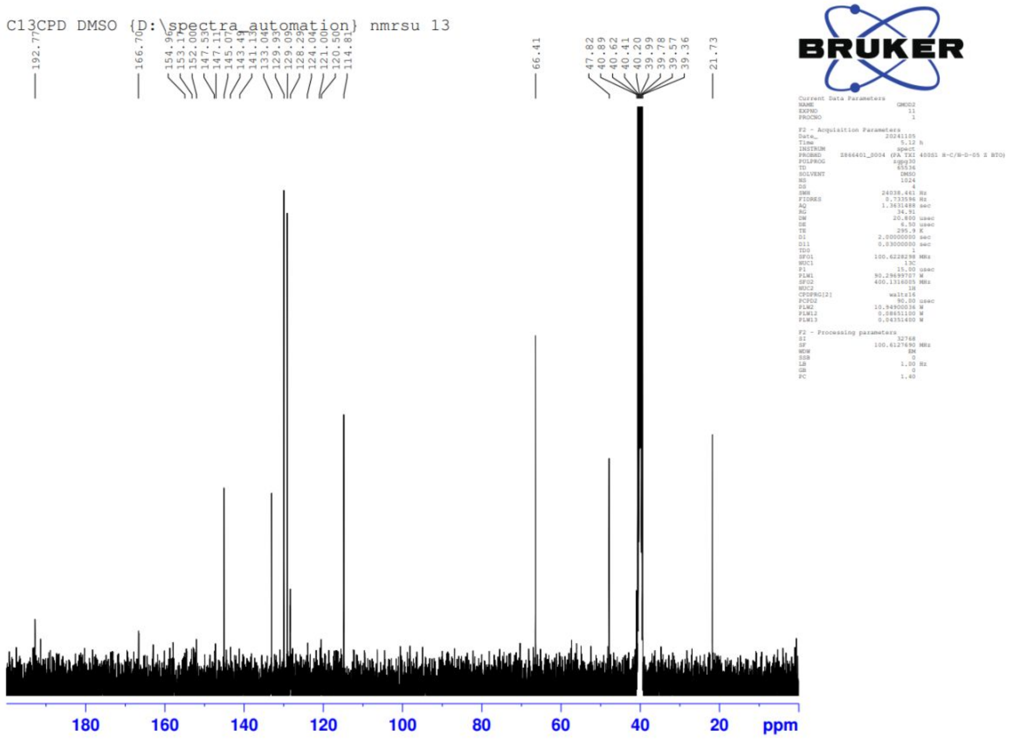

Figure S9. ^{13}C-NMR spectral analysis of compound **5b**.

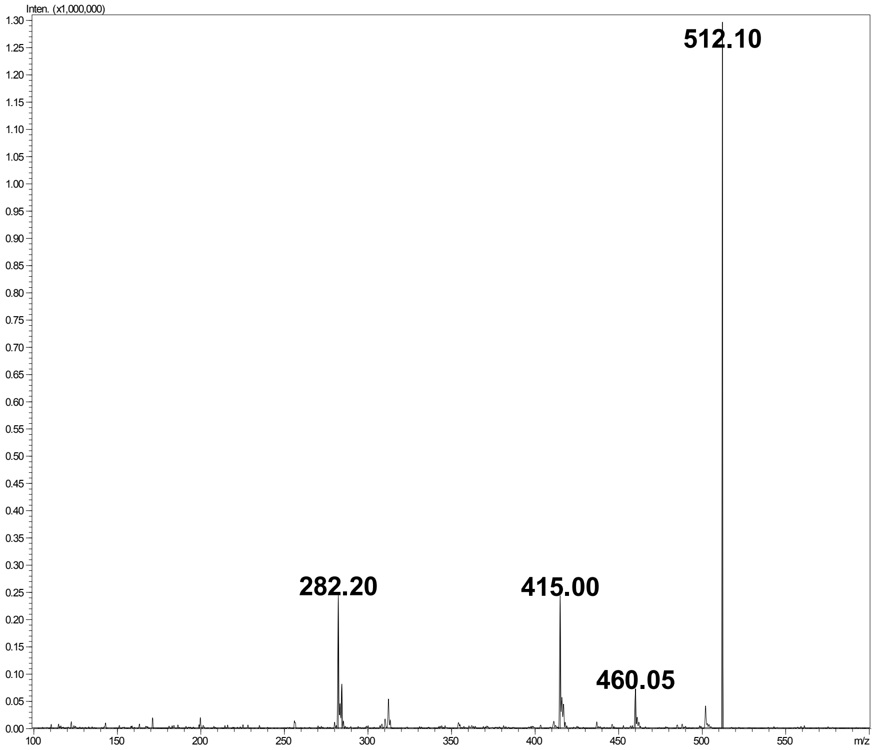

Figure S10. LC-MS spectral analysis of compound **5b**.

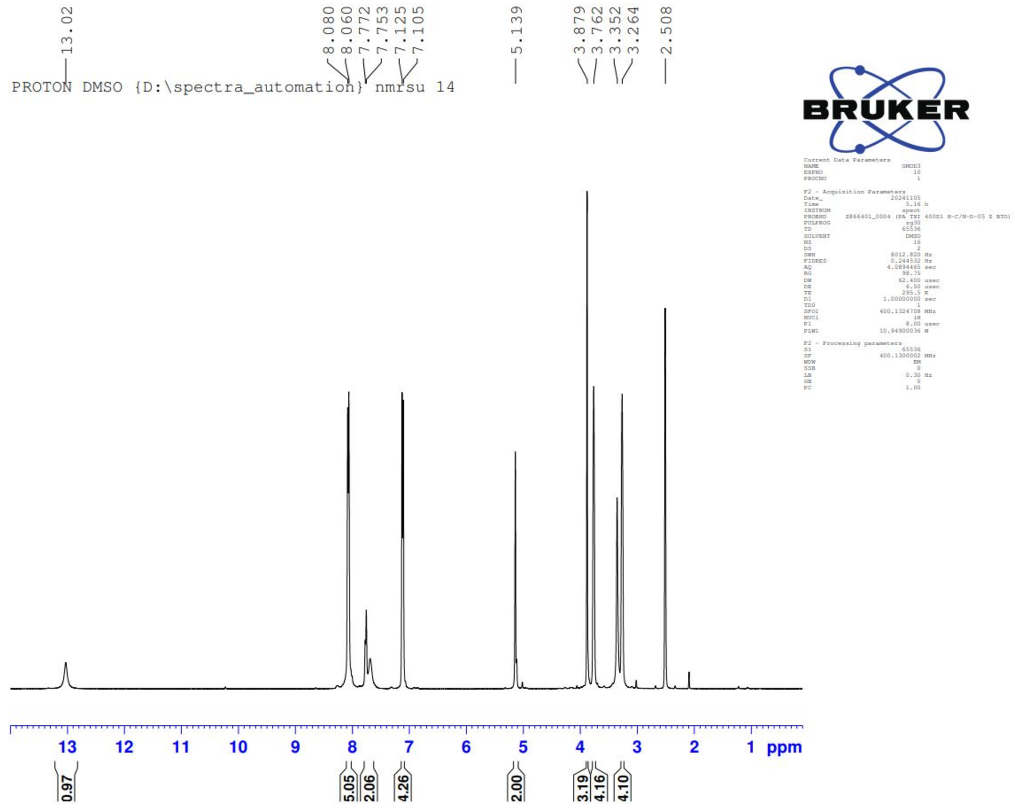

Figure S11. ^{1}H-NMR spectral analysis of compound **5c**.

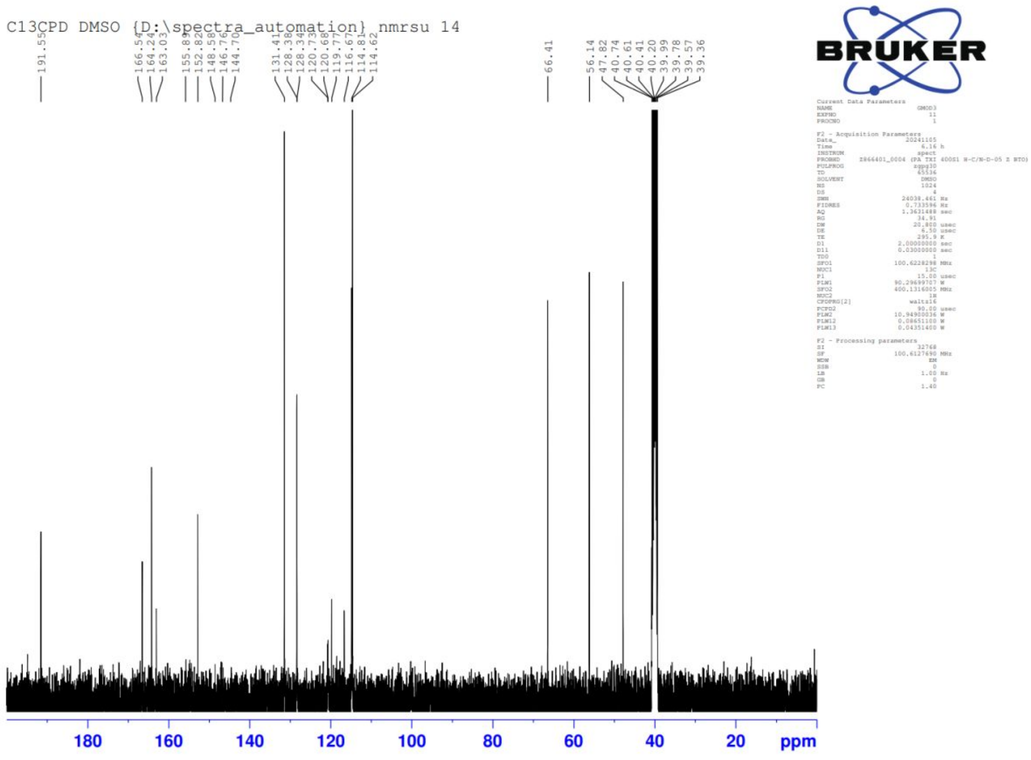

Figure S12. ^{13}C-NMR spectral analysis of compound **5c**.

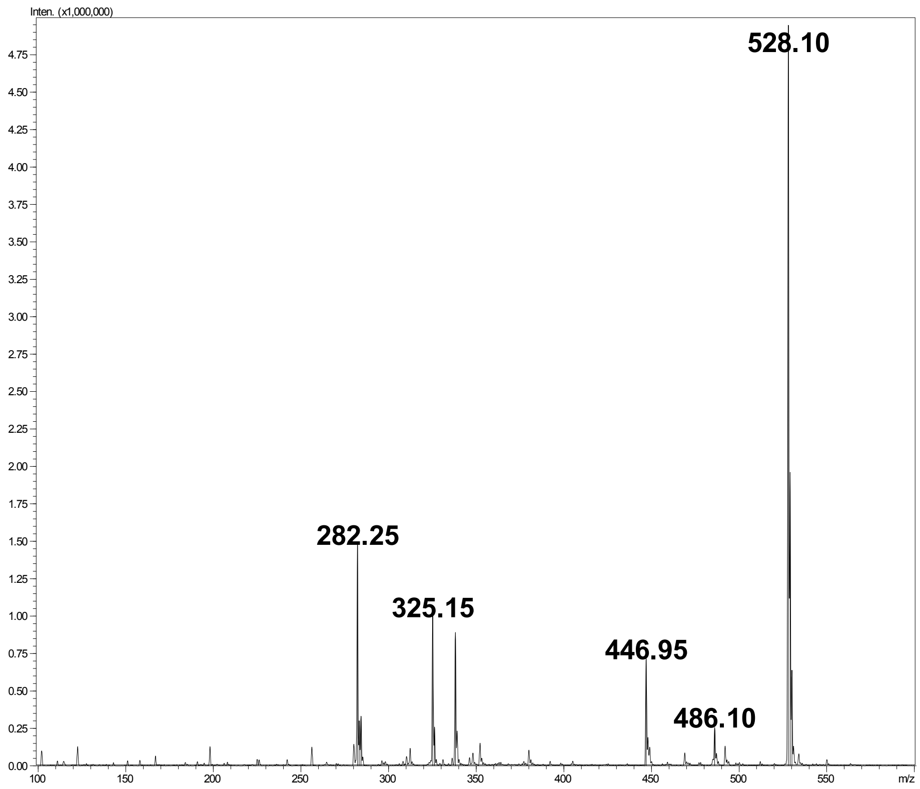

Figure S13. LC-MS spectral analysis of compound **5c**.

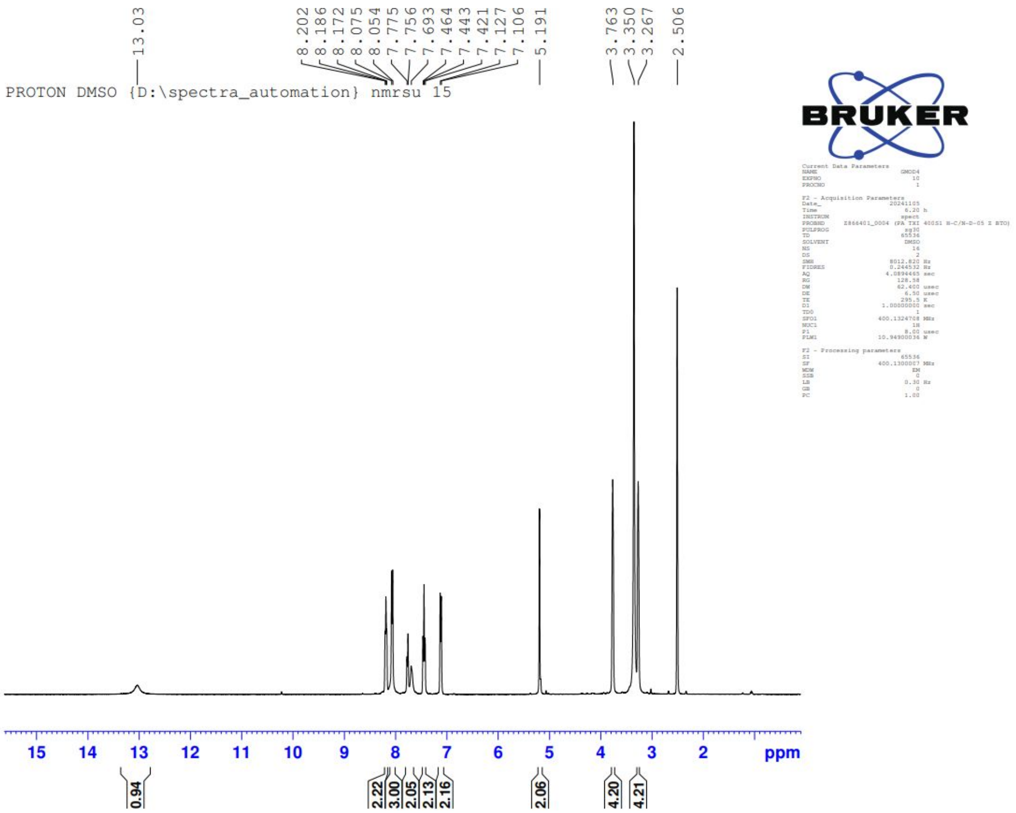

Figure S14. ^{1}H-NMR spectral analysis of compound **5d**.

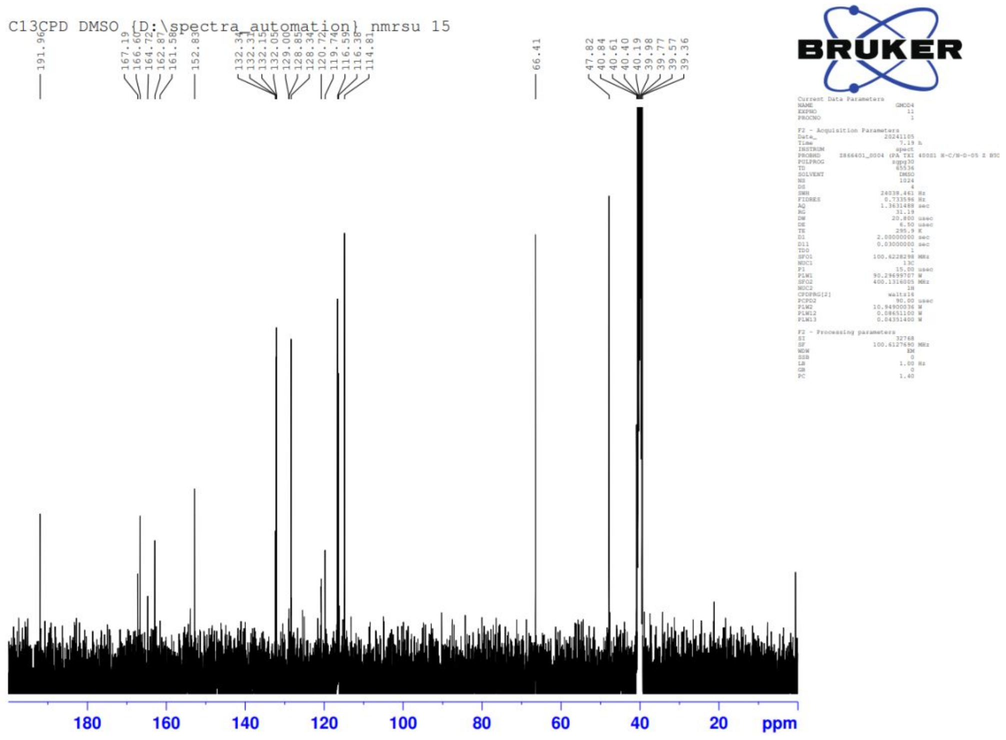

Figure S15. ^{13}C-NMR spectral analysis of compound **5d**.

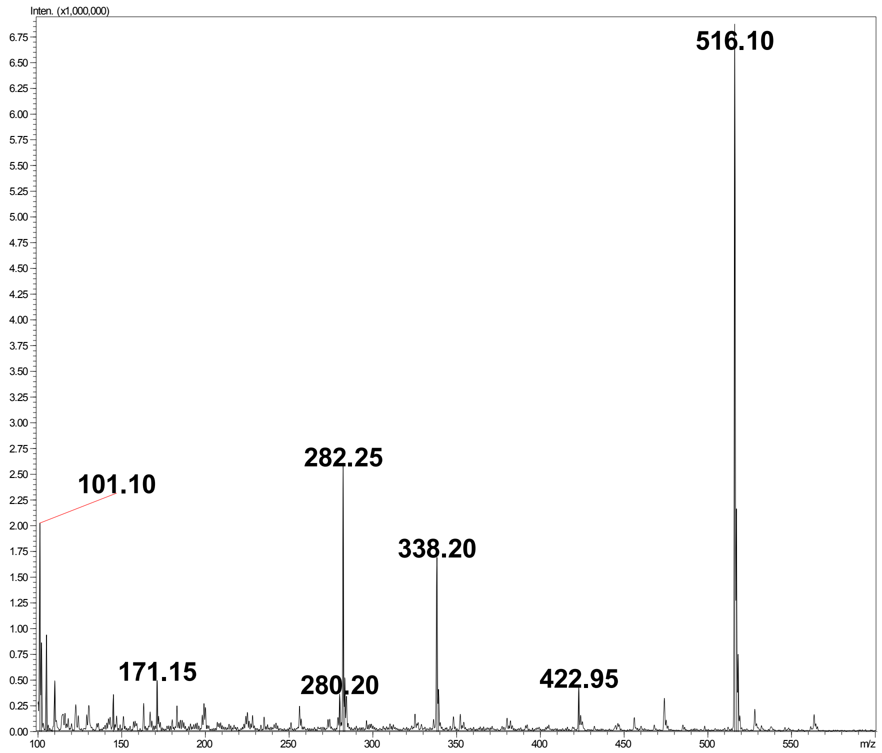

Figure S16. LC-MS spectral analysis of compound **5d**.

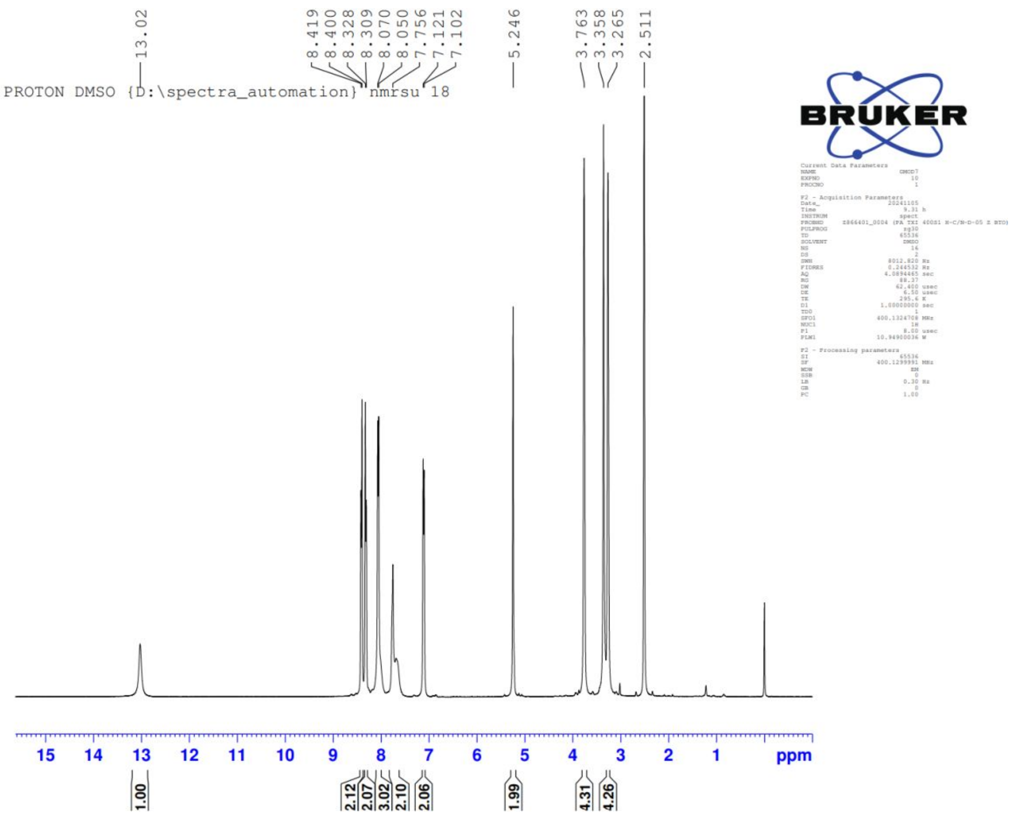

Figure S17. ^{1}H-NMR spectral analysis of compound **5e**.

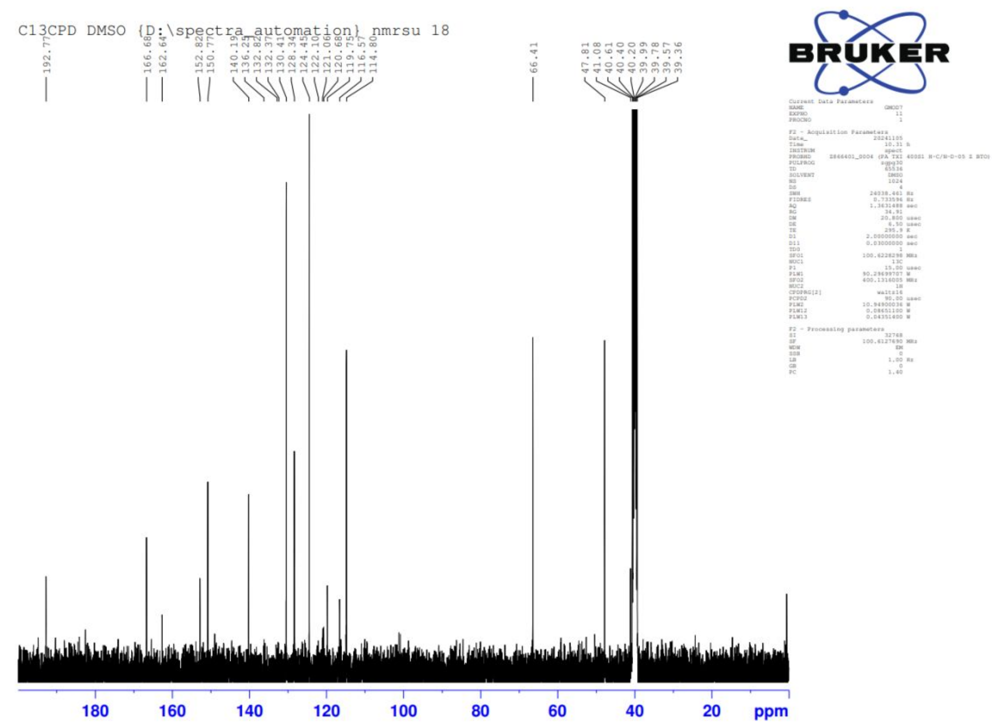

Figure S18. ^{13}C-NMR spectral analysis of compound **5e**.

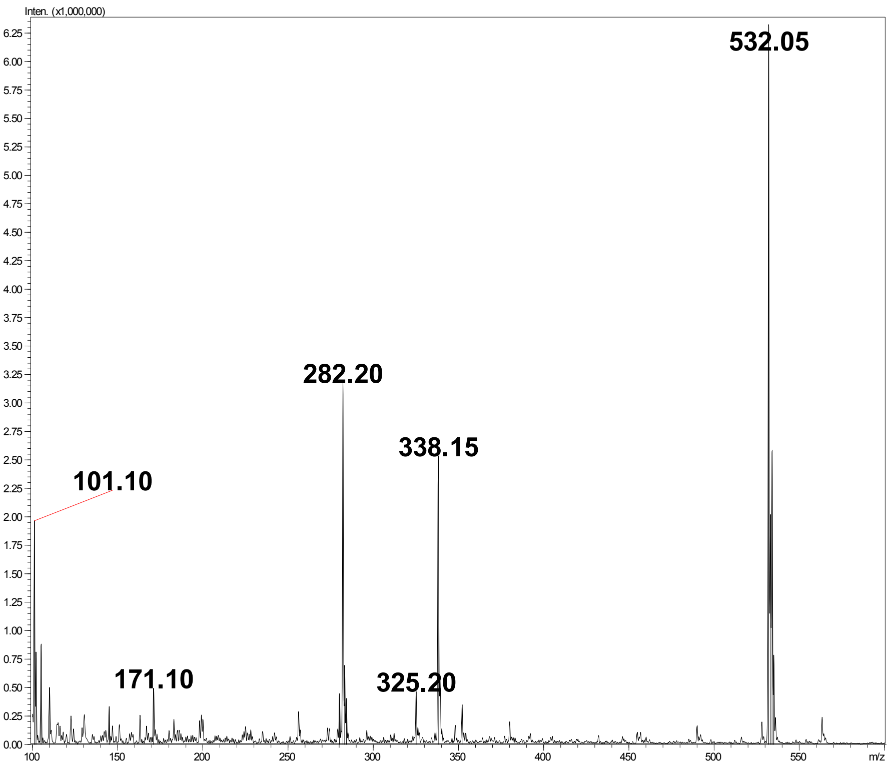

Figure S19. LC-MS spectral analysis of compound **5e**.

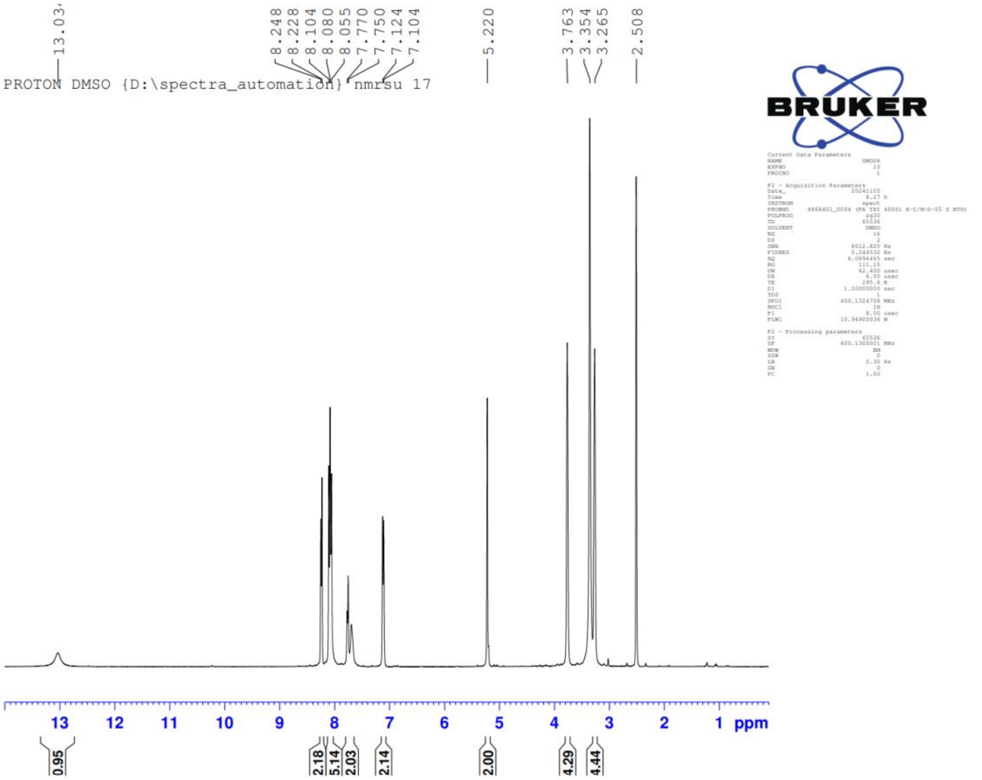

Figure S20. ^{1}H-NMR spectral analysis of compound **5f**.

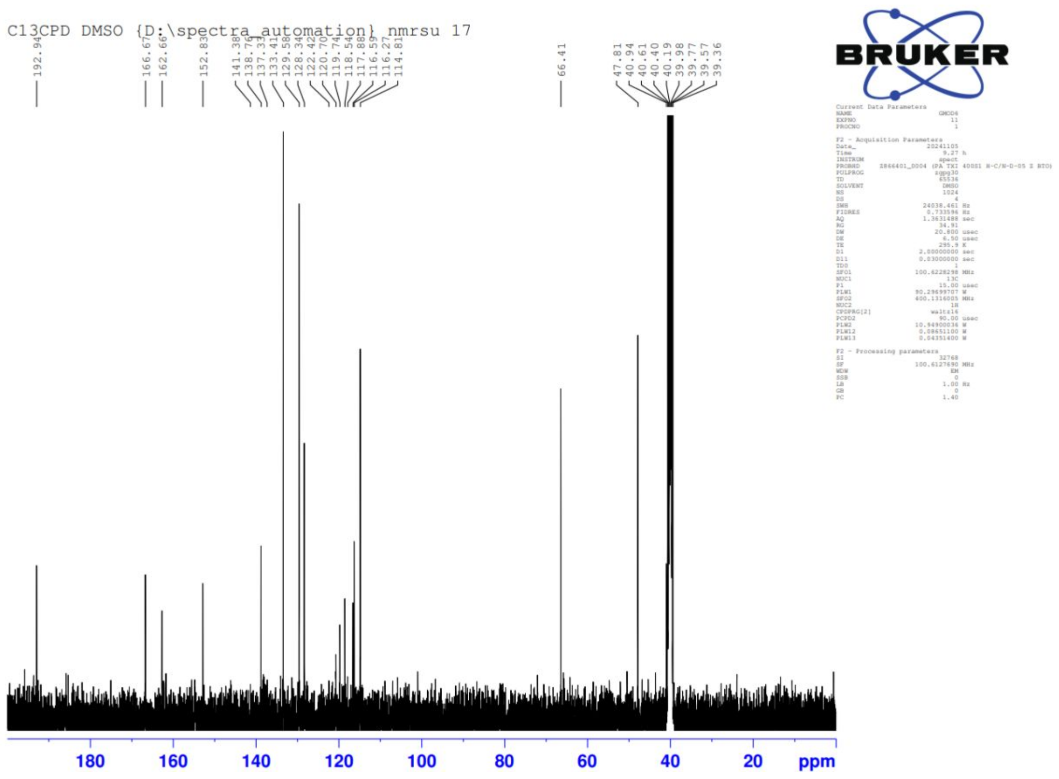

Figure S21. ^{13}C-NMR spectral analysis of compound **5f**.

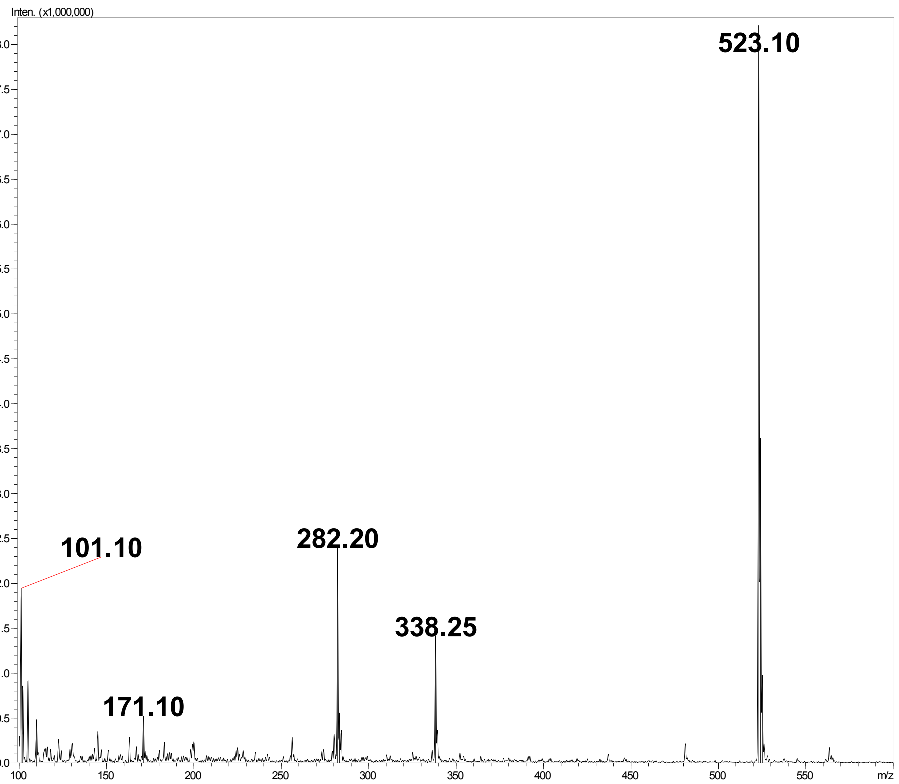

Figure S22. LC-MS spectral analysis of compound **5f**.

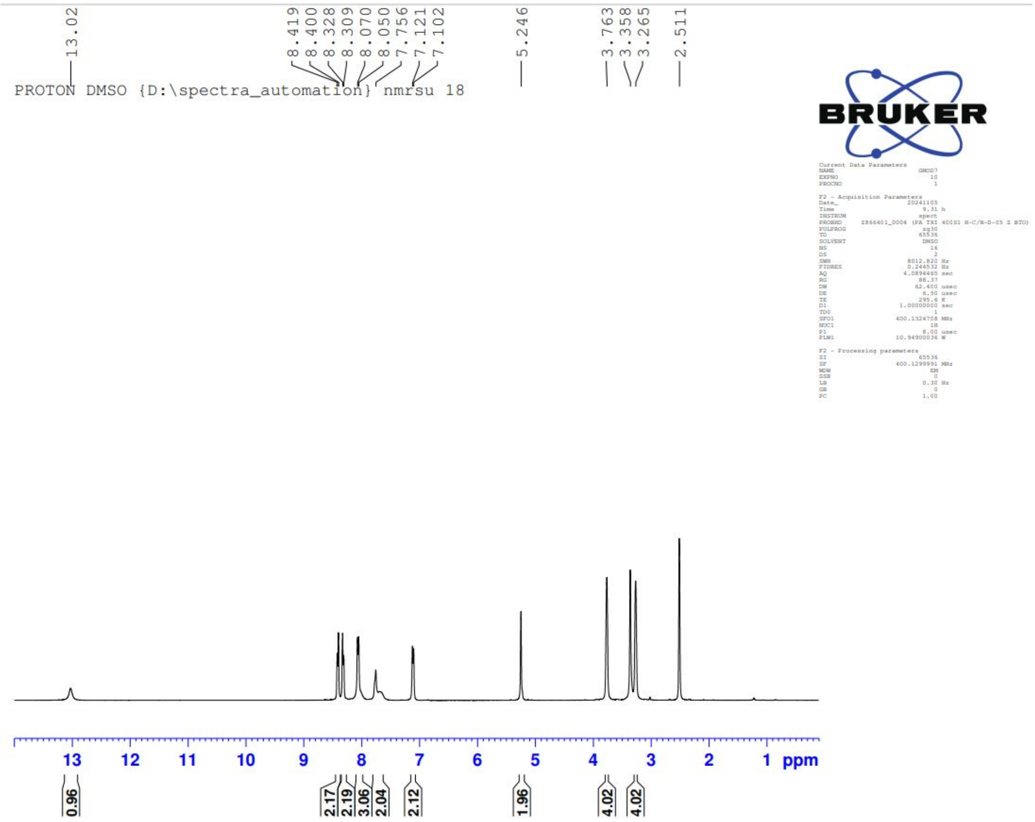

Figure S23. ^{1}H-NMR spectral analysis of compound **5g**.

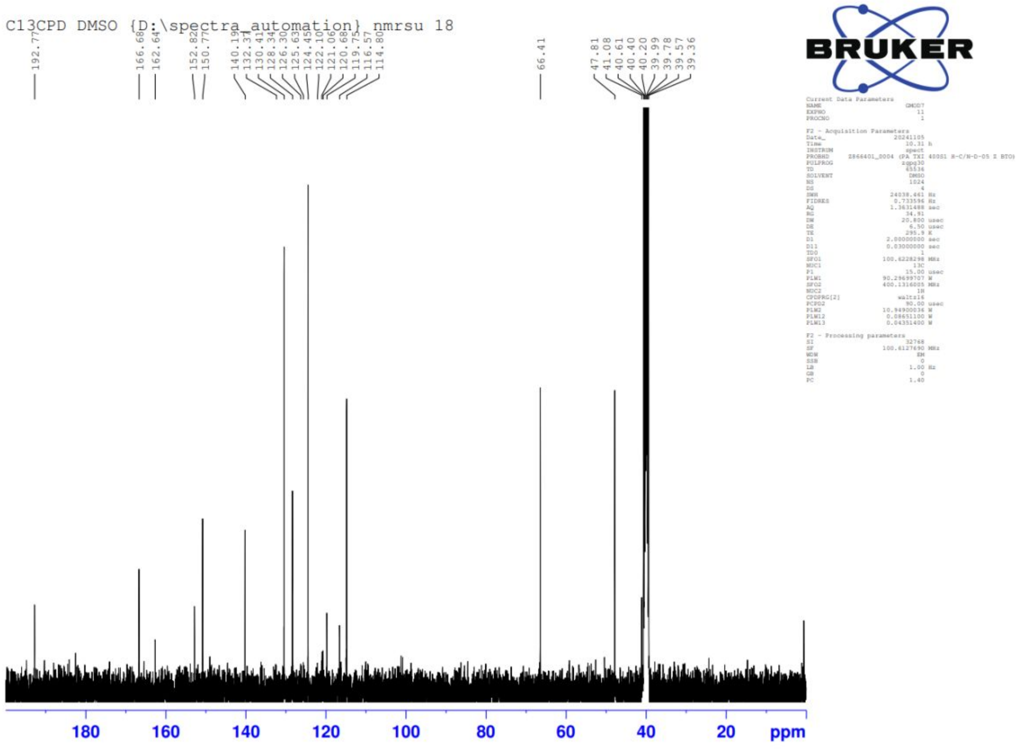

Figure S24. ^{13}C-NMR spectral analysis of compound **5g**.

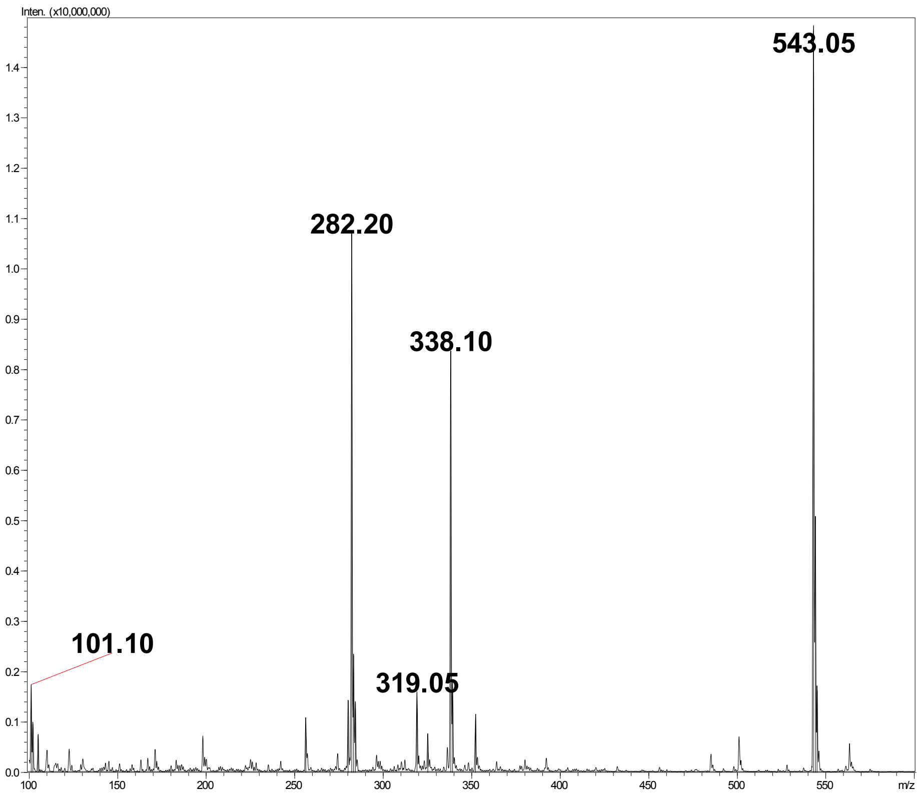

Figure S25. LC-MS spectral analysis of compound **5g**.

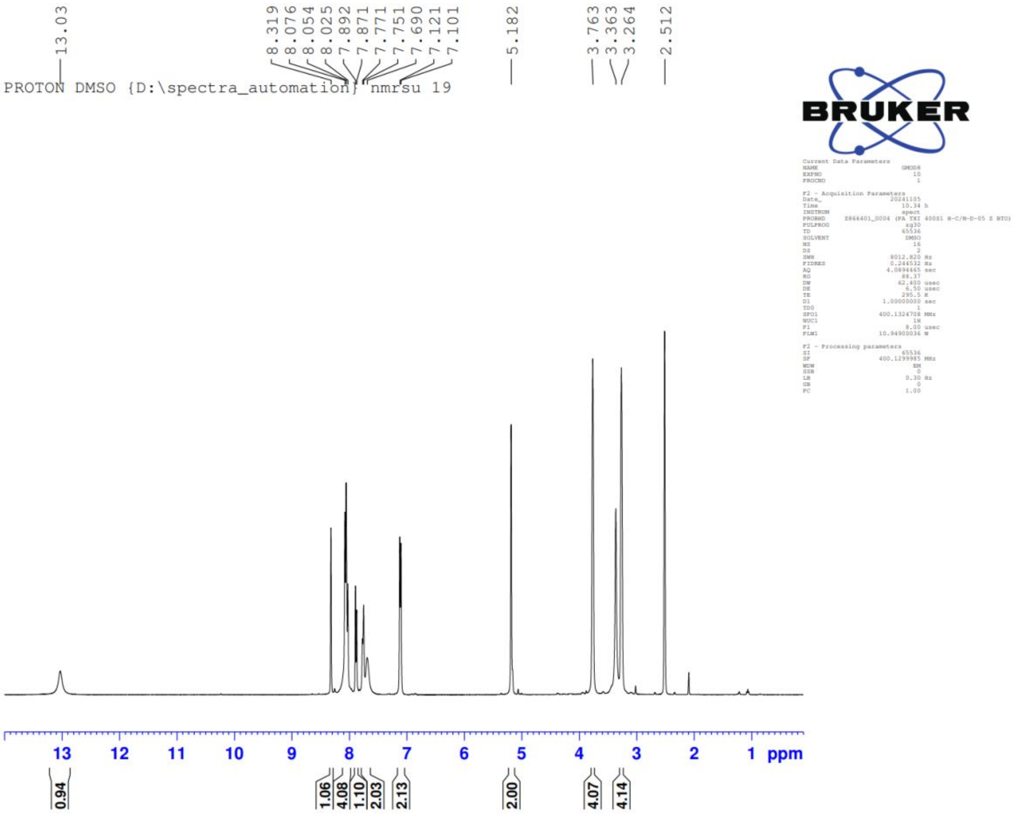

Figure S26. ^{1}H-NMR spectral analysis of compound **5h**.

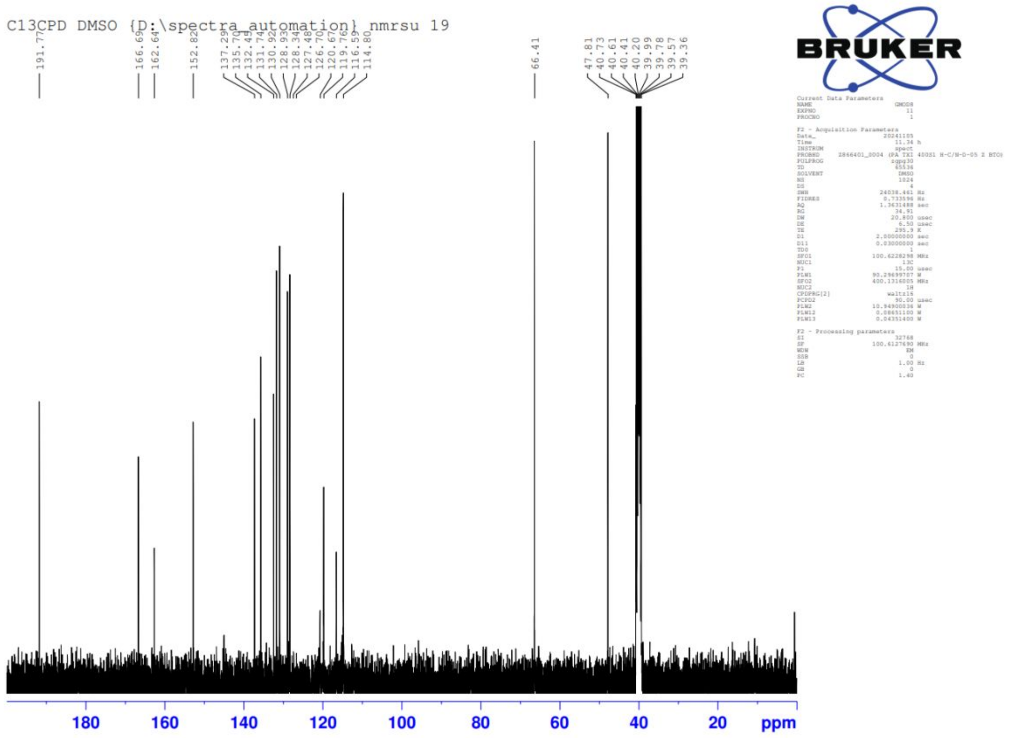

Figure S27. ^{13}C-NMR spectral analysis of compound **5h**.

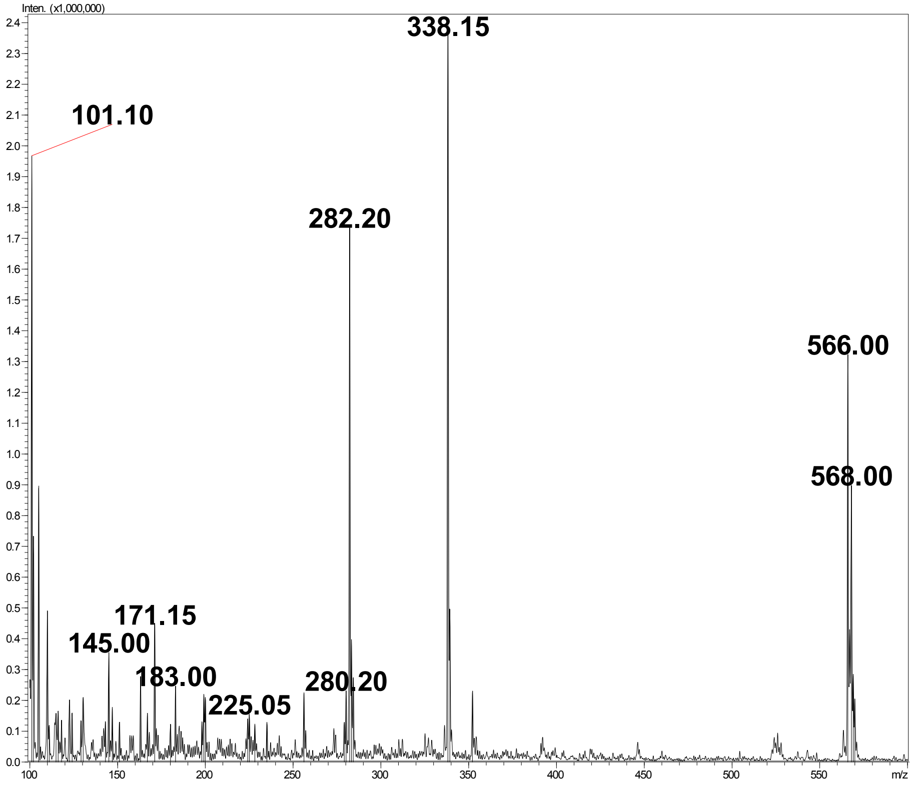

Figure S28. LC-MS spectral analysis of compound **5h**.

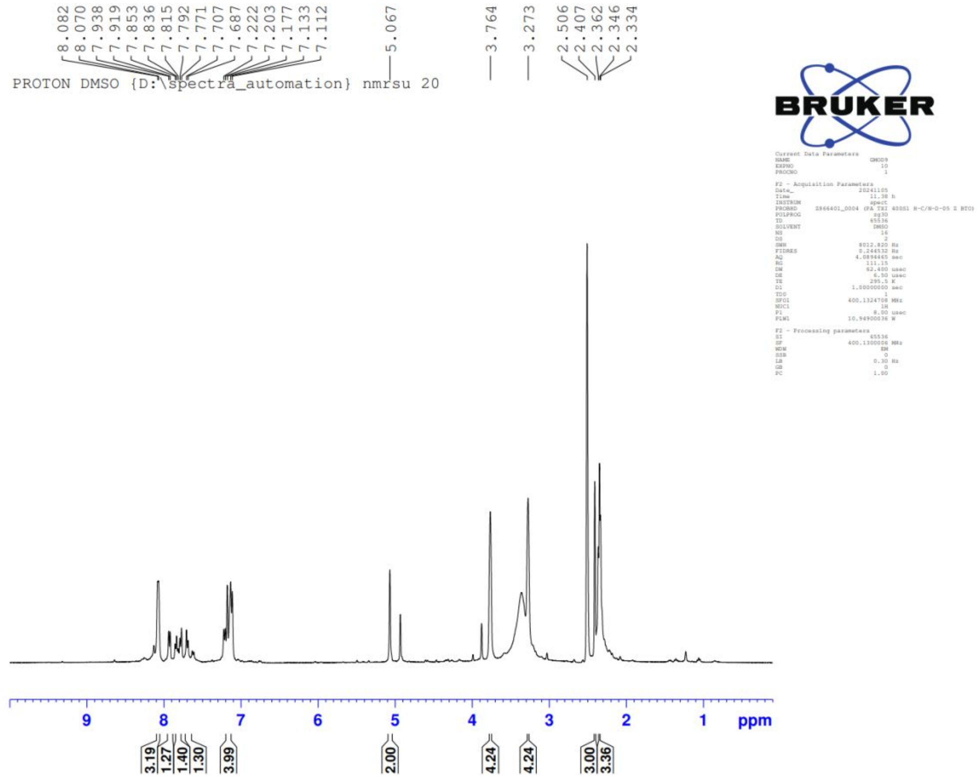

Figure S29. ^{1}H-NMR spectral analysis of compound **5i**.

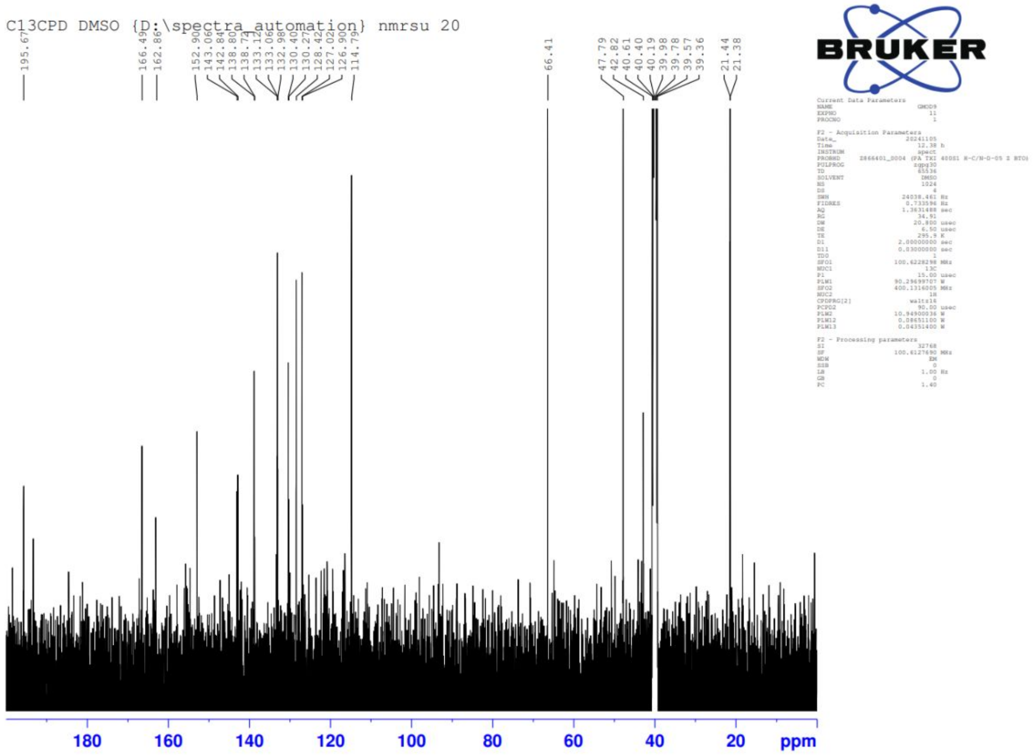

Figure S30. ^{13}C-NMR spectral analysis of compound **5i**.

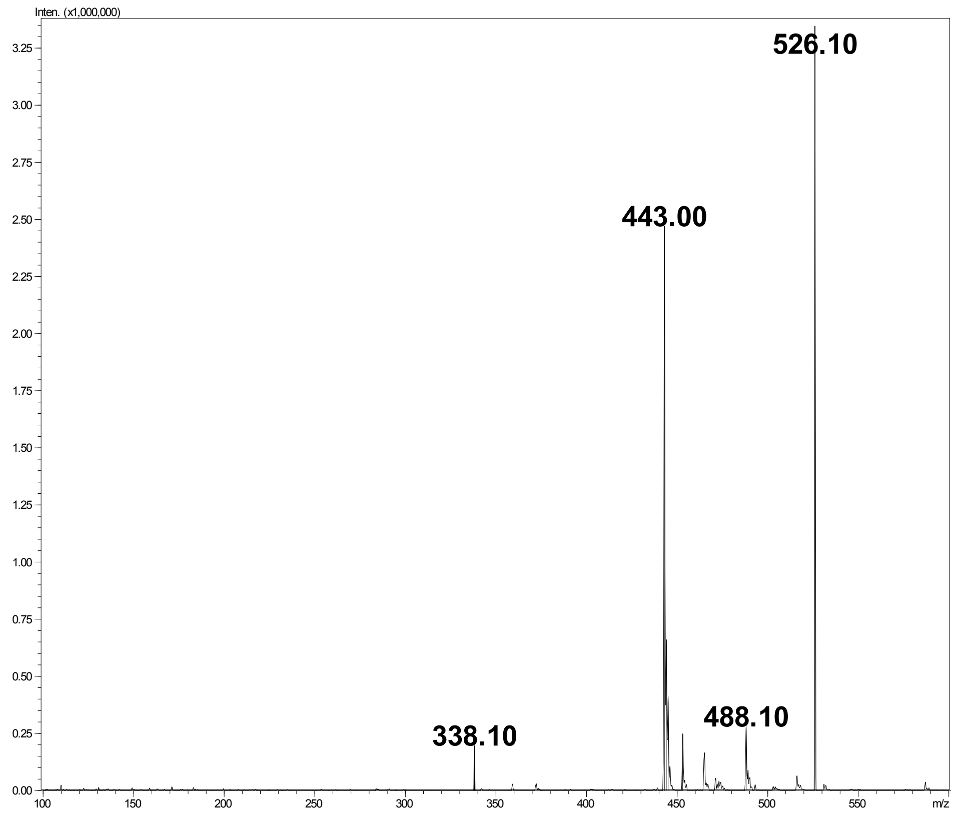

Figure S31. LC-MS spectral analysis of compound **5i**.

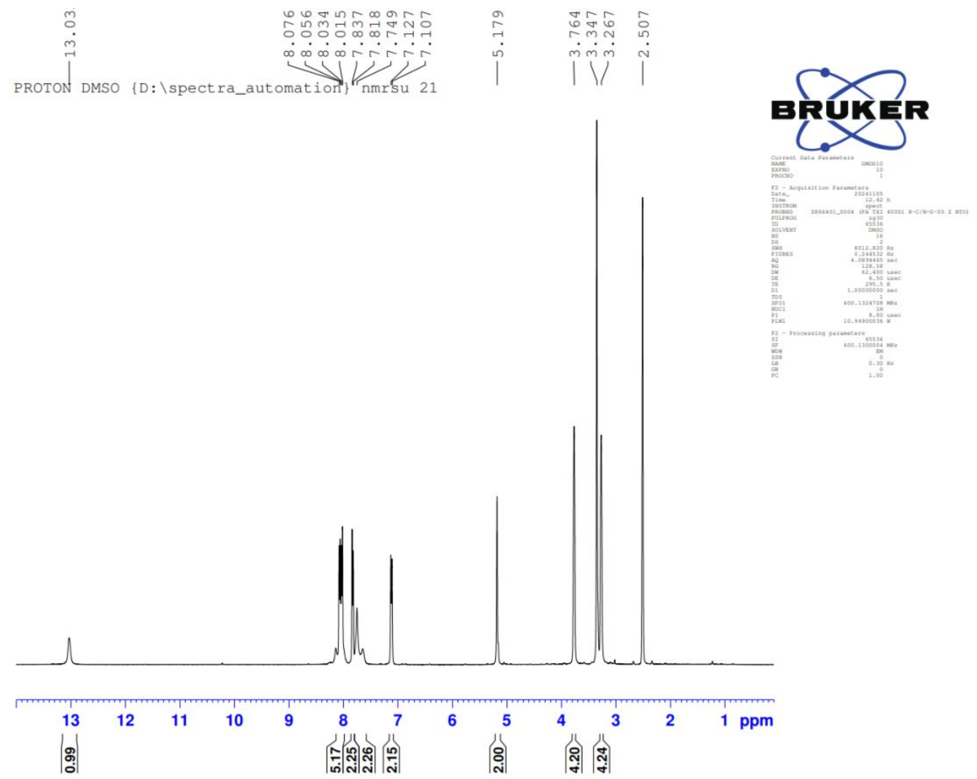

Figure S32. ^{1}H-NMR spectral analysis of compound **5j**.

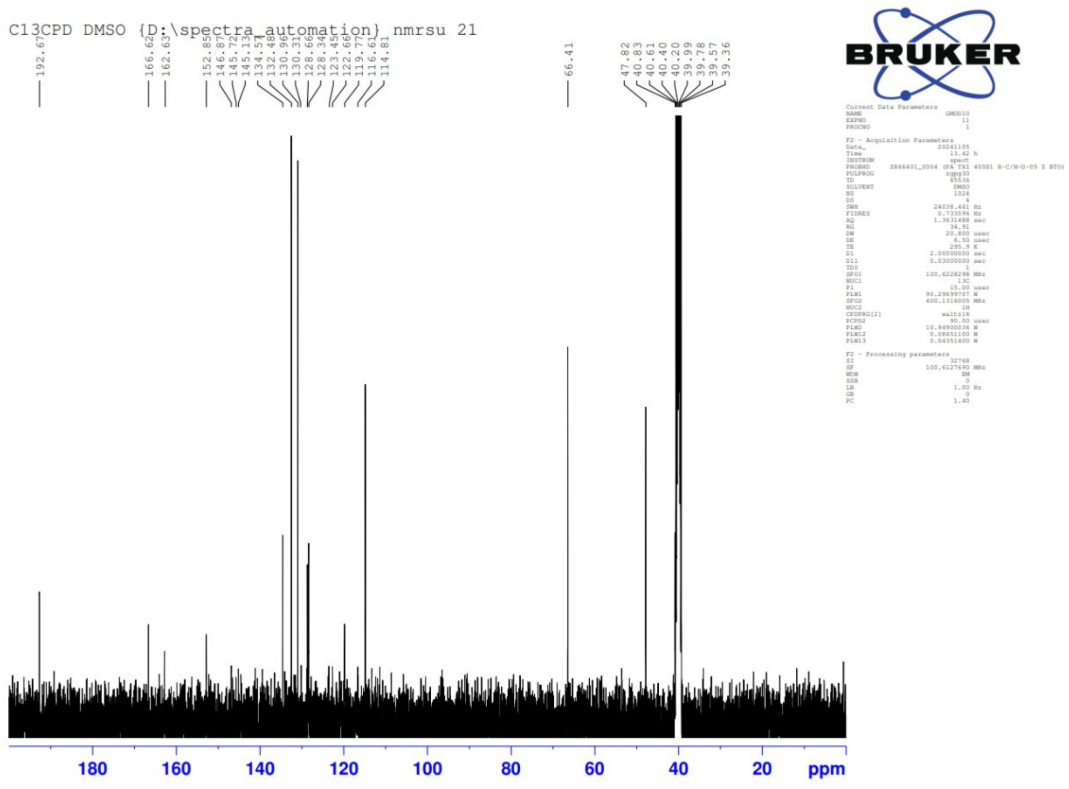

Figure S33. ^{13}C-NMR spectral analysis of compound **5j**.

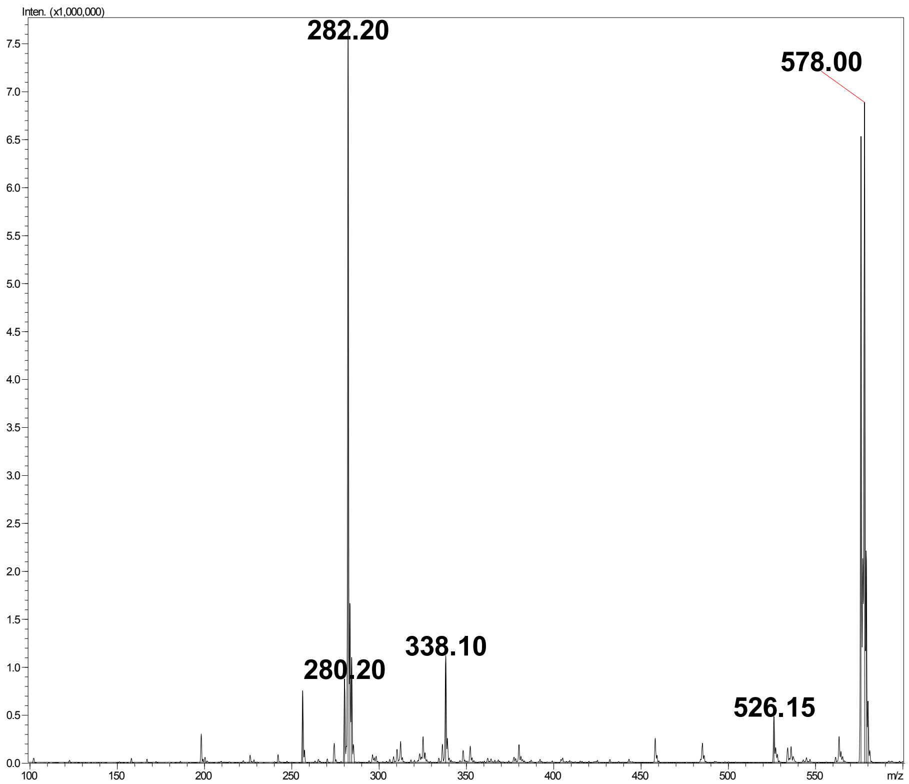

Figure S34. LC-MS spectral analysis of compound **5j**.

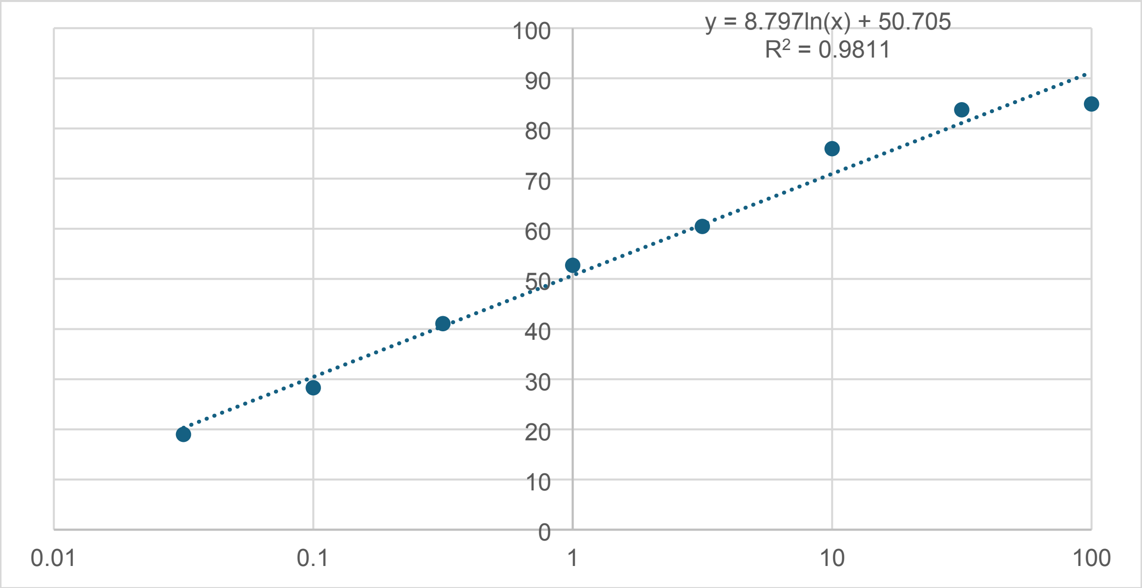

Figure S35. IC_{50} graph of compound **5c** versus for VEGFR

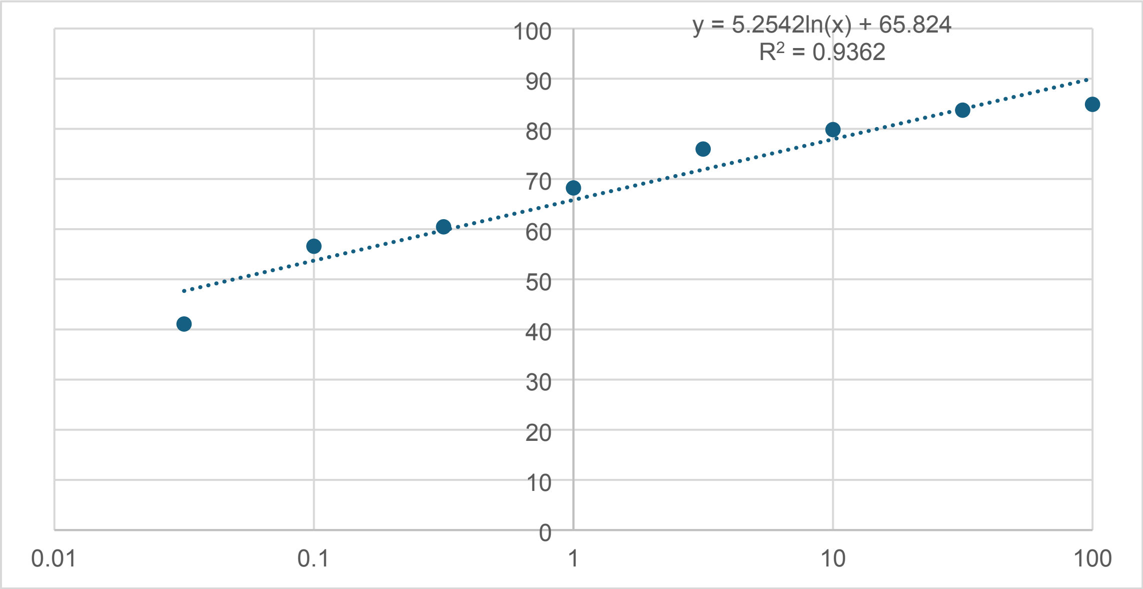

Figure S36. IC_{50} graph of compound **5h** versus for VEGFR

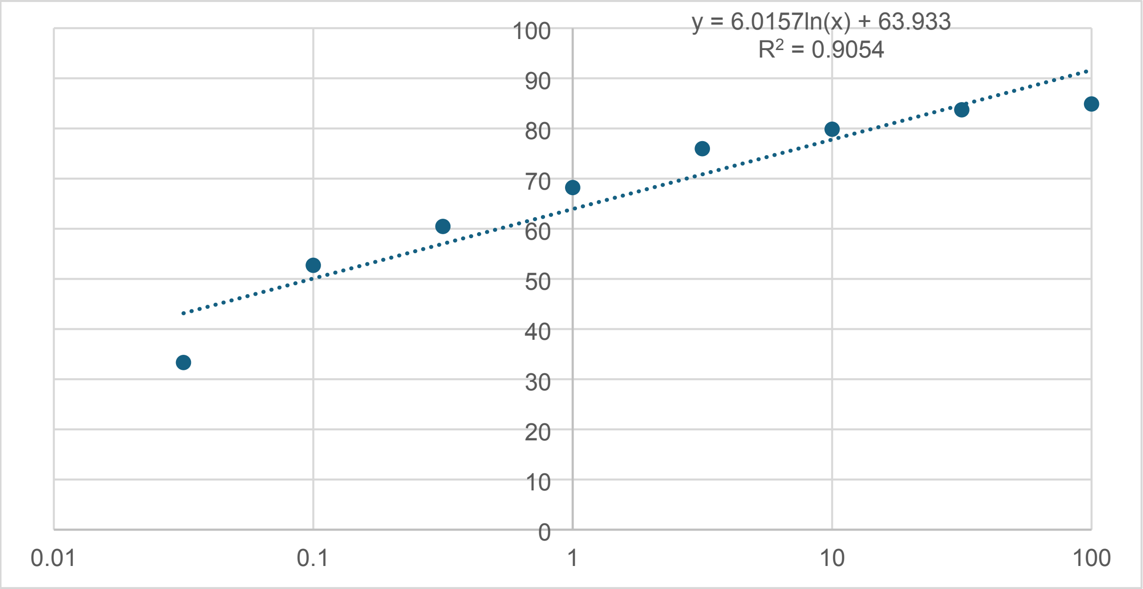

Figure S37. IC_{50} graph of compound **5j** versus for VEGFR

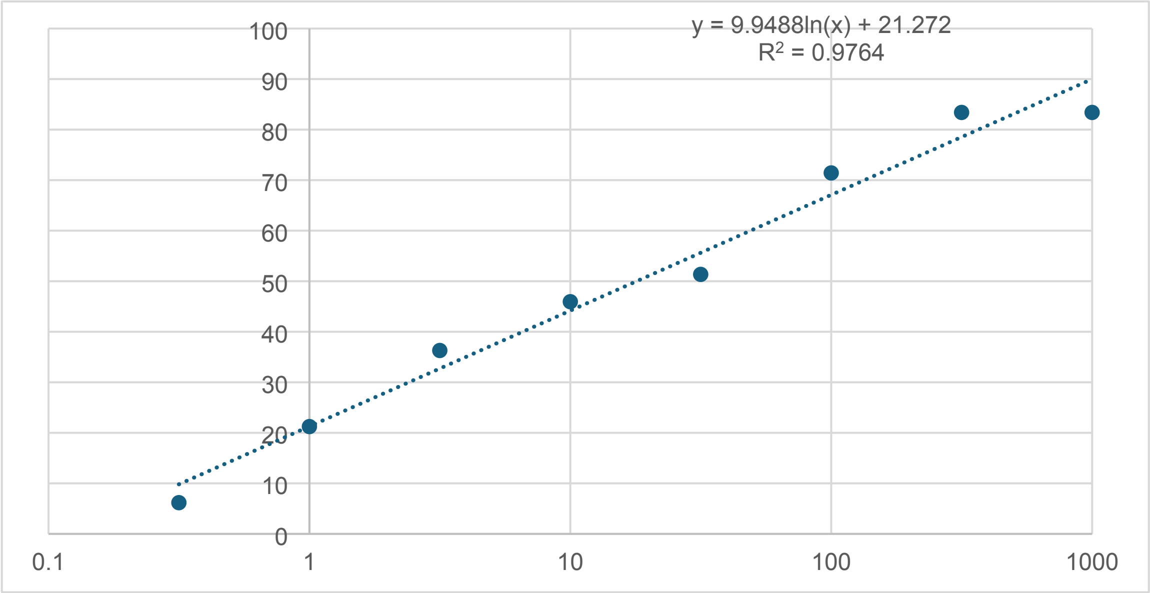

Figure S38. IC_{50} graph of compound **5c** versus HT29 cell line

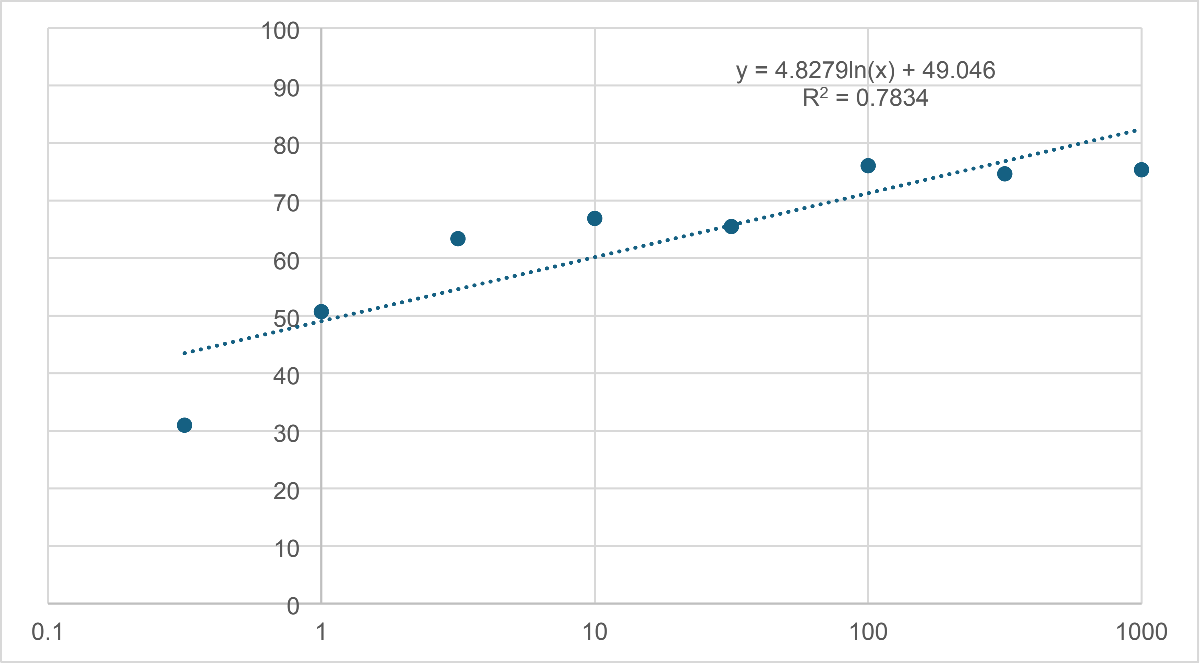

Figure S39. IC_{50} graph of compound **5c** versus NIH3T3 cell line

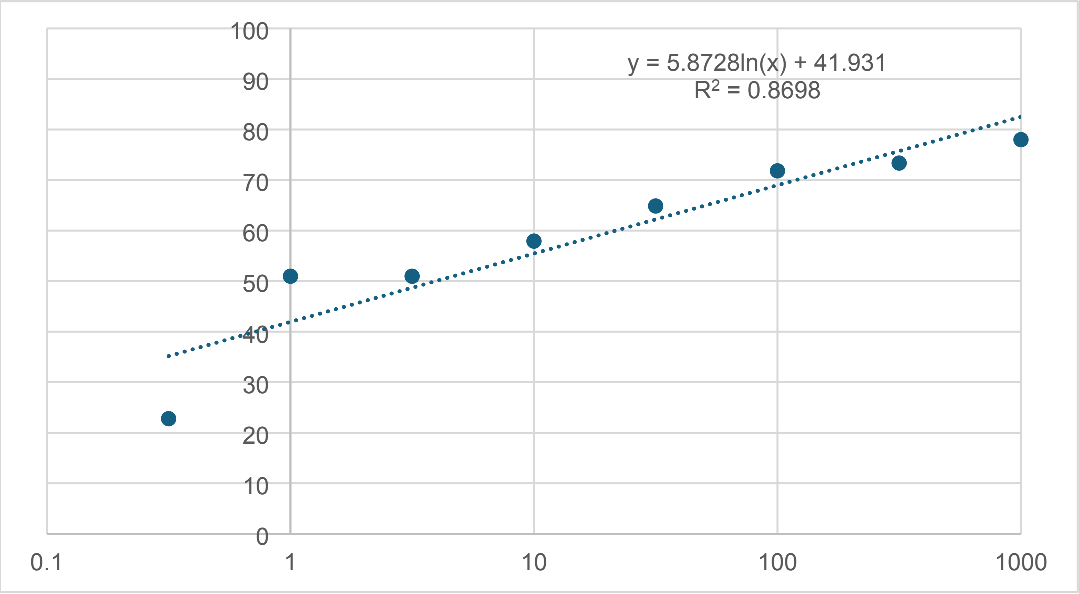

Figure S40. IC_{50} graph of compound **5h** versus HT29 cell line

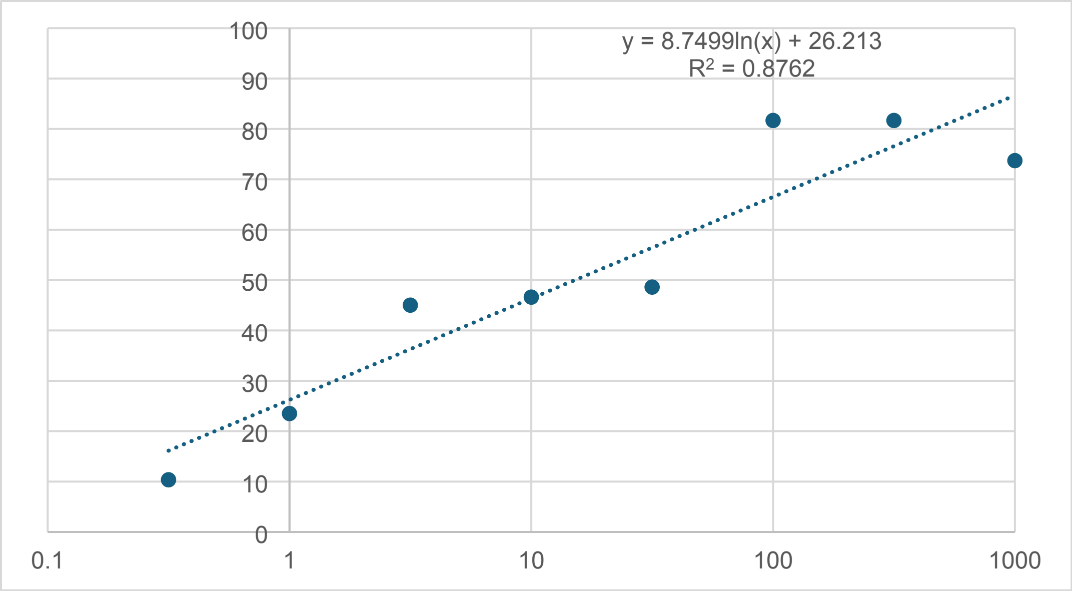

Figure S41. IC_{50} graph of compound **5h** versus NIH3T3 cell line

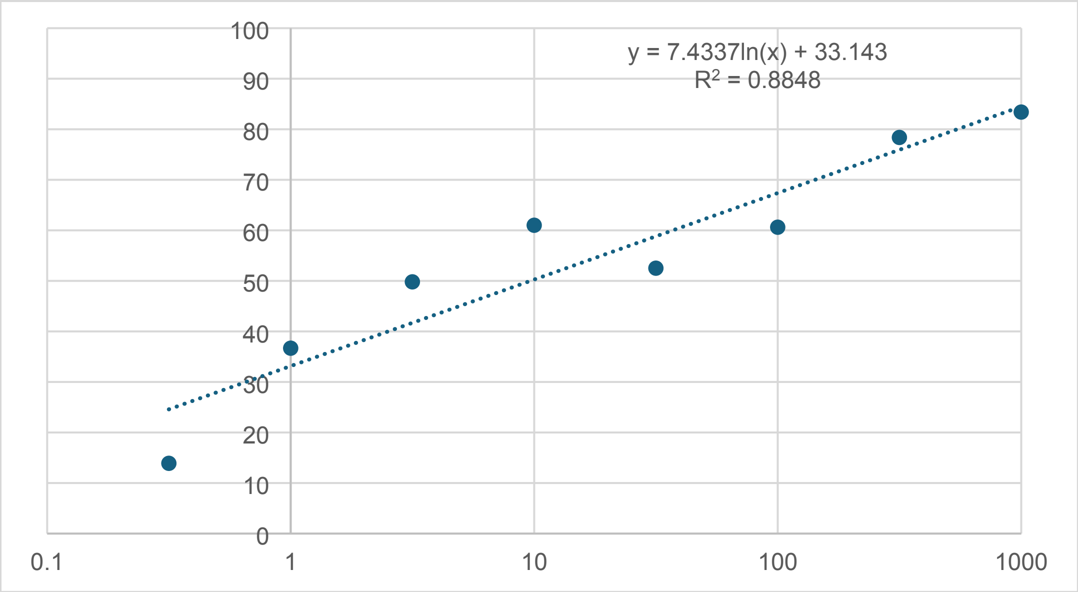

Figure S42. IC_{50} graph of compound **5j** versus HT29 cell line

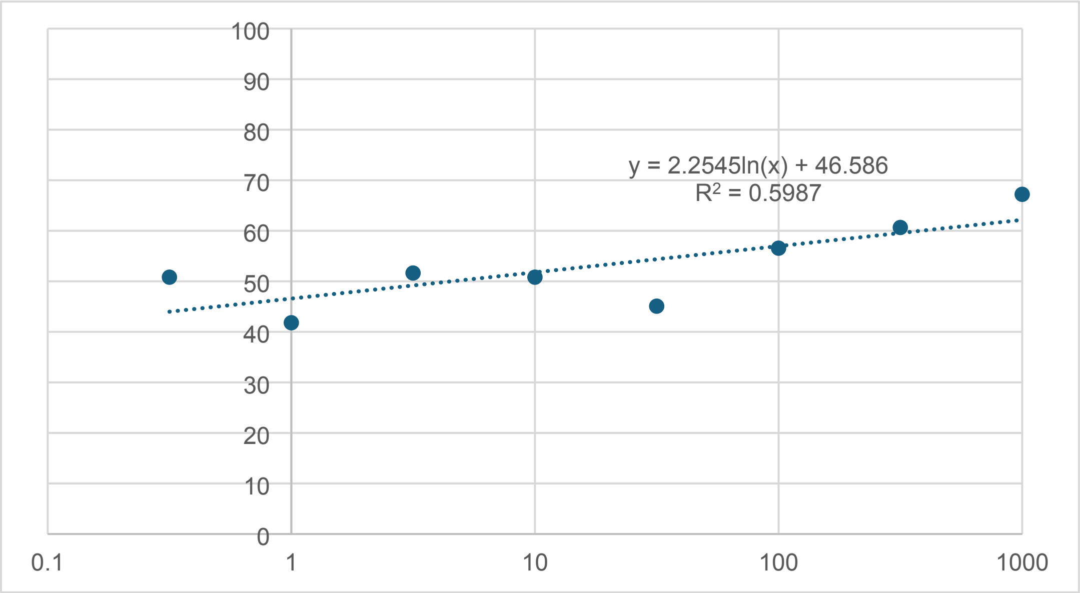

Figure S43. IC_{50} graph of compound **5j** versus NIH3T3 cell line
